# Selective Oxidation Reactions of Natural Compounds with Hydrogen Peroxide Mediated by Methyltrioxorhenium

**DOI:** 10.3390/molecules181113754

**Published:** 2013-11-07

**Authors:** Maria E. Amato, Francesco P. Ballistreri, Andrea Pappalardo, Gaetano A. Tomaselli, Rosa M. Toscano, Giuseppe Trusso Sfrazzetto

**Affiliations:** Dipartimento di Scienze Chimiche, Università di Catania, Viale A. Doria 6, Catania 95125, Italy; E-Mails: eamato@unict.it (M.E.A.); fballistreri@unict.it (F.P.B.); andrea.pappalardo@unict.it (A.P.); gtomaselli@unict.it (G.A.T.); giuseppe.trusso@unict.it (G.T.S.)

**Keywords:** natural compounds oxidation, hydrogen peroxide, methyltrioxorhenium

## Abstract

We have investigated the oxidative behaviour of natural compounds such as methyl abietate (**1**), farnesyl acetate (**2**), α-ionone (**3**), β-ionone (**4**), methyl linolelaidate (**5**), methyl linolenate (**6**) and bergamottin (**7**) with the oxidant system methyltrioxo-rhenium/H_2_O_2_/pyridine. The reactions, performed in CH_2_Cl_2_/H_2_O at 25 °C, have shown good regio- and stereoselectivity. The oxidation products were isolated by HPLC or silica gel chromatography and characterized by MS(EI), ^1^H-, ^13^C-NMR, APT, gCOSY, HSQC, TOCSY and NOESY measurements. The selectivity seems to be controlled by the nucleophilicity of double bonds and by stereoelectronic and steric effects.

## 1. Introduction

Oxyfunctionalization of cheap natural compounds is a useful protocol to obtain molecules widely employed in the fine chemicals-based industries as fragrances, flavors, and therapeutically active substances [1]. The most commonly employed stoichiometric oxidants are organic peroxy- acids, particularly *m*-chloroperbenzoic acid (MCPBA). However, these oxidants are economically unattractive and are not selective for the preparation of acid-sensitive epoxides [2].

Methyltrioxorhenium (CH_3_ReO_3_, MTO) in the presence of H_2_O_2_ has proven itself as an efficient and versatile oxidation catalyst with interesting selectivity towards natural compounds, which can be oxidized under quite mild conditions [3,4,5,6,7,8,9,10,11,12,13,14,15,16,17,18,19,20,21]. In previous work [22] we have observed good regio- and stereoselectivity in the oxidation reactions by MTO/H_2_O_2_ of steroidal compounds such as 7-dehydrocholesteryl or ergosteryl acetate and the vitamins D2 and D3. 

The active species involved in the oxygen transfer to the olefinic double bond are probably a monoperoxo complex [MeRe(O)_2_(O_2_)] and a diperoxo complex [MeRe(O)(O_2_)_2_], obtained respectively by the addition of one or two H_2_O_2_ molecules to MTO [23]. However, depending on the nature of the epoxide, a ring opening catalyzed by the Re(VII) metal center can also occur to give 1,2-diols [3]. Moreover, the epoxide ring opening can be minimized by employing pyridine as a basic ligand. Mechanistic investigations [24], incorporating the positive pyridine effect [16], showed that the added pyridine minimizes also the MTO decomposition to perrhenate (ReO_4_^−^) [25,26,27,28,29]. In this report we have investigated the oxidation reactions of some natural compounds by MTO/H_2_O_2_/pyridine, leading to products of practical interest or of interest as synthons in the synthesis of fine chemicals, with the aim of assessing the parameters controlling the process.

## 2. Results and Discussion

**Figure 1:**
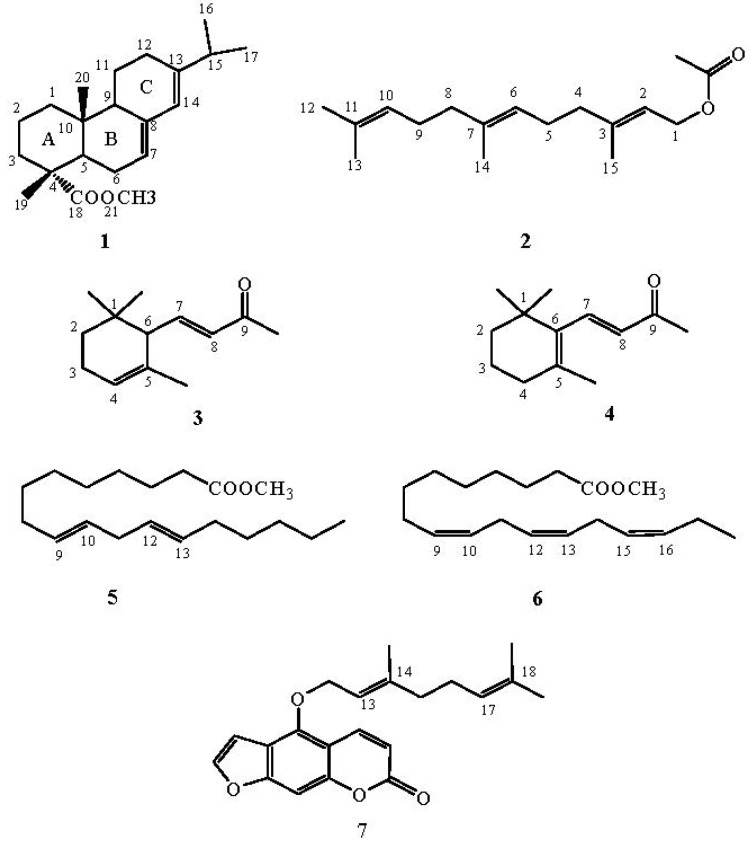
Selected natural compounds which undergo oxidation reactions with MTO/H_2_O_2_/pyridine.

We have investigated the behaviour of methyl abietate (**1**), farnesyl acetate (**2**), α-ionone (**3**), β-ionone (**4**), methyl linolelaidate (**5**), methyl linolenate (**6**) and bergamottin (**7**) (Figure 1) which underwent oxidation reactions with MTO/H_2_O_2_/pyridine in CH_2_Cl_2_ at 25 °C (Table 1).

**Table 1:** Oxidation products of selected natural compounds with MTO/H_2_O_2_/pyridine in CH_2_Cl_2_ at 25 °C ^a^.

Entry	Time (h)	Conv. (%)		Product selectivities (%)	
**1**	0.5	88	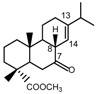	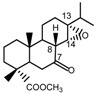	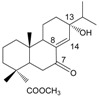
			**8** (53)	**9** (28)	**10** (19)
**2**	0.5	95	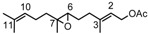	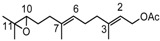	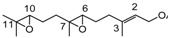
			**11** (32)	**12** (26)	**13** (42)
**3**	18	100	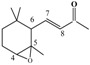	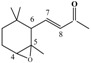	
			**14** *cis* (86)	**15** *trans* (14)	
**4**	20	75	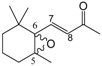		
			**16** (100)		
**5**	8	99	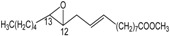	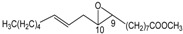	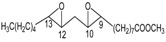
			**17** (22)	**18** (20)	**19** (58)
**6** ^b^	8	98	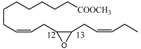	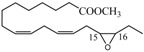	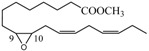
			**20** (18)	**21** (17)	**22** (18)
**7**	4	62	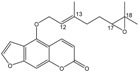		
			**23** (100)		

Entries **1**–**7** referred to the starting natural compounds reported in Figure 1, respectively. ^a^ Substrate/H_2_O_2_/MTO/pyridine (1:1.5:0.05:0.12); ^b^ A mixture of bisepoxides is also obtained (47%).

### 2.1. Methyl Abietate (**1**)

**Scheme 1:**
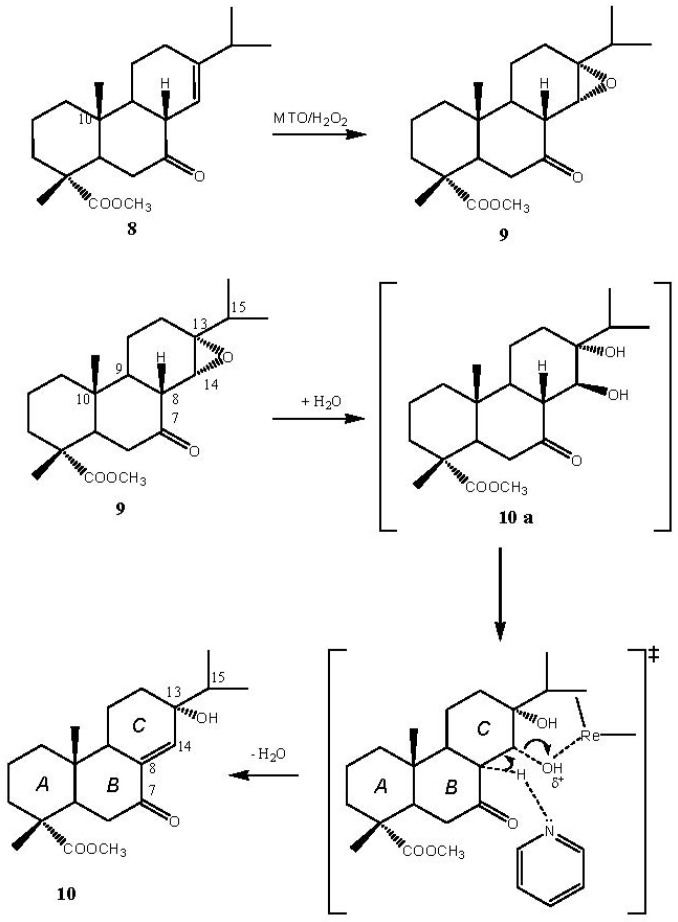
Suggested formation pathway for the oxidation product **8**.

The oxyfunctionalization of methyl abietate (**1**), the diterpene which is the main component of rosin acids, is of interest in the research field on separation of rosin acids from pine oleoresin—based on double bond oxidation processes—and in the low cost synthesis of rosin acid derivatives having multiple functional groups. The oxidation of methyl abietate with MTO/H_2_O_2_/pyridine leads to synthons for the stereoselective syntheses of bioactive natural compounds. Ketone **8**, derived from the oxidation of ring B, is the main product. Formation of **8** has been already observed by Haslinger *et al*. [30]. Probably, the first step of the reaction involves the formation of the epoxide obtained by electrophilic oxygen transfer to the double bond of ring B. Subsequent reorganization of the epoxide mediated by the presence of the rhenium derivative (Lewis acid) leads to the formation of the ketone **8** (Scheme 1).

**Scheme 2:**
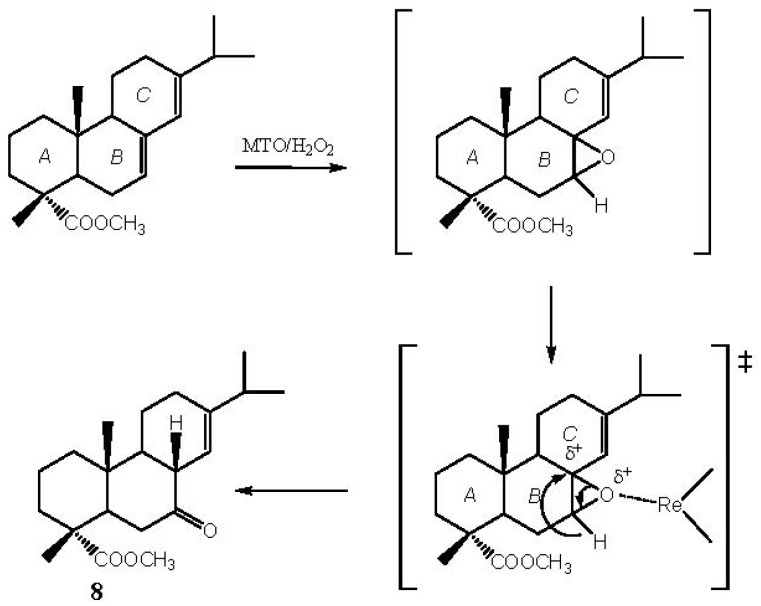
Suggested pathway of formation of γ-hydroxyketone **10**.

Lewis acid promoted rearrangement of epoxides into carbonyl compounds is an important and well known reaction [31,32] utilised in many cases for the synthesis of biologically active compounds. The remaining oxidation products **9** [33,34] and **10** are secondary oxidation products derived from the further oxidation of **8** (Scheme 2).

The diastereoselective formation of compound **9** by oxidation of **8** is probably due to the presence of the methyl group in position 10 which makes the attack of the upper face of the ring *C* by the bulky oxidant reagent (rhenium peroxide) sterically hard. The formation of the α-epoxide is supported by the ^1^H-NMR signal of H-14 which appears as a singlet rather than a doublet indicating that there is no coupling with H-8 because the two protons form a 90° dihedral angle. Hydration of ketoepoxide **9** leads to the formation of diol **10a**, which undergoes a 1,2-elimination assisted by rhenium and pyridine to yield the γ-hydroxyketone **10**. Since it is reported [33,34] that when the OH group of the γ-hydroxyketone **10** linked at the C-13 atom is in axial position the ^1^H-NMR signal of H-14 occurs at 6.75 ppm, we assume that in our case this OH group is in equatorial position because H-14 signal shifts upfield to 5.26 ppm.

### 2.2. All-Trans Farnesyl Acetate (**2**)

Farnesyl epoxides are very useful starting compounds for the biomimetic synthesis of a large variety of natural monocyclic terpenoids [35,36]. Oxidation of farnesyl acetate (**2**) afforded the two monoepoxides **11** [37] and **12** [35,37] and a mixture of stereoisomeric diepoxides **13** [38] (see Table 1). The formation of the oxidation products, in agreement with the electrophilic oxygen transfer mechanism, is controlled by the nucleophilicity of double bonds. Therefore the reaction occurs in a regioselective manner at the double bonds C10-C11 and C6-C7 since the double bond C2-C3 is less reactive because is located nearby an electron withdrawing functional group.

The formation of the mixture of stereoisomeric diepoxides **13** was confirmed by the presence in the ^1^H-NMR spectrum (see ESI, S18) of six singlets assigned to the three methyl groups linked to carbons C-11 and C-7 of the two three-membered rings, respectively.

### *2.3. α-Ionone* (**3**) *and β-Ionone* (**4**)

**Figure 2:**
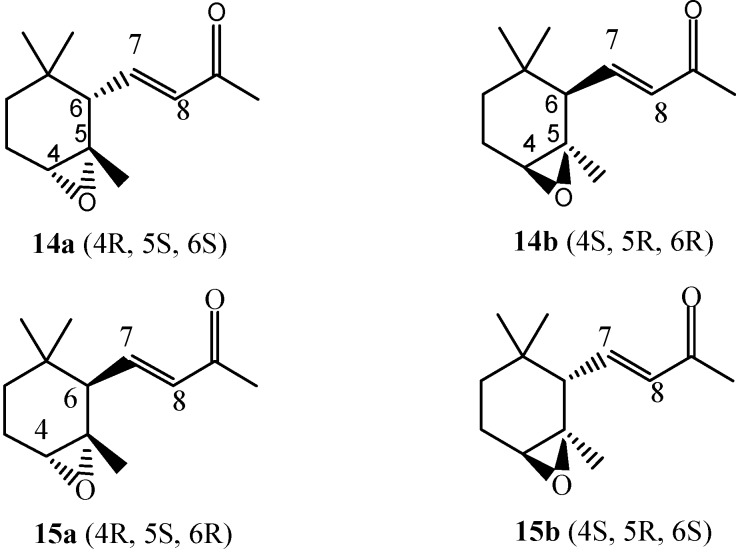
Oxidation products of α-ionone **3**.

α-Ionone (**3**) undergoes the oxidation reaction in a very good selective manner to yield mainly the monoepoxide **14** [39] (racemic *cis*-4,5-epoxy-4,5-dihydro-α-ionone) and small amounts of monoepoxide **15** [39] (racemic *trans-*4,5-epoxy-4,5-dihydro-α-ionone) (*cis*/*trans* ratio ~6), shown in Figure 2.

**Scheme 3:**
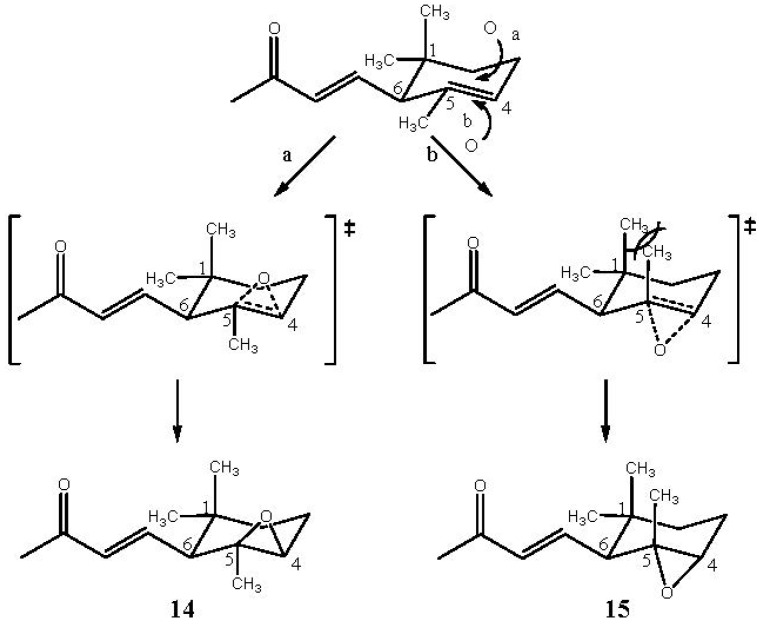
Suggested pathway for the formation of monoepoxides **14** and **15**.

The reaction is regioselective because the double bond C7-C8 is not involved in the reaction due to its lower nucleophilicity due to the presence of the carbonyl group. Similar results have already been observed using *m*-chloroperbenzoic acid as oxidant (*cis*/*trans* ratio = 5) [39]. The high face selectivity (Table 1) is probably controlled by the larger crowding in the transition state leading to the *trans* epoxide which increases the activation energy and makes unfavourable the formation of the corresponding isomer. In fact, as Scheme 3 shows, during the oxygen transfer, the C-5 (as well as the C-4) undergoes a rehybridation from sp^2^ to sp^3^ and the methyl group linked to C-5, which in the transition state forming the *trans* epoxide is going to occupy an opposite position with respect to that of the incoming oxidant, assumes an axial direction parallel to that of one of the methyl groups linked to C-1, developing therefore repulsive interactions (1,3-diaxial interactions).

On the other hand, the oxidation of β-ionone (**4**) is highly regioselective because the double bond C5-C6 is quite more nucleophilic than the C7-C8 double bond (which bears an electron withdrawing carbonyl functionality in the α-position) and therefore the epoxide **16** [40] is obtained as the solae product (see Table 1).

### 2.4. Methyl Linolelaidate (**5**)

Oxidation of methyl linolelaidate (**5**) afforded the two monoepoxides **17** (9-undecenoic acid, 11-(3-pentyloxiranyl) methyl ester) and **18** [41,42] (oxiraneoctanoic acid, 3-(2-octenyl)-methyl ester), and, as main product, a mixture of two diastereoisomer diepoxides **19** [41,42] (methyl 9,10-12,13-diepoxyoctadecenoate) obtained by a further oxidation of both **17** and **18** epoxides.

### 2.5. Methyl Linolenate (**6**)

Oxidation of methyl linolenate leads to the nearly equal formation of three monoepoxides: 9-undecenoic acid,11-[3-(2-pentenyl)oxiranyl]-methyl ester **20** [43,44], 9,12-tetradecadienoic acid,14-(3-ethyloxiranyl)- methyl ester (9*Z*,12Z) **21** [43,44], and oxiraneoctanoic acid, 3-(2,5-octadienyl)methyl ester [2*S*[2α,3α(2*Z*,5*Z*)]] **22** [43,44] according to the similar nucleophilicity of the corresponding double bonds.

### 2.6. Bergamottin (**7**)

**Figure 3:**
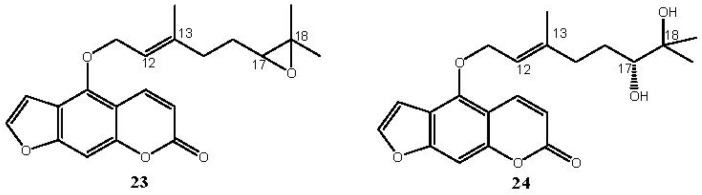
Chemical structures of bergamottin derivatives **23** and **24**.

The bergamottin is a member of the furanocoumarin family and is most commonly found in grapefruit juice. Along with the chemically related compound 17,18-dihydroxybergamottin (**24**, Figure 3), it is believed to be responsible for the inhibitory effects of grapefruit juice on cytochrome P450 enzyme activity interfering therefore on the metabolism of a variety of pharmaceutical drugs [45,46].

Hence the need to provide easy and very selective synthetic routes for **24**. Since the configuration of the C-17 of **24**, isolated from both grapefruit juice and its peel oil [47], is *R*, we have developed a synthetic strategy to obtain **24** (yield 5%) by the highly regioselective oxidation of the C17-C18 double bond of bergamottin (**7**) with MTO/H_2_O_2_/pyridine to yield the racemic epoxide **23** and subsequent hydrolytic kinetic resolution (HKR) catalyzed by chiral (*S*,*S*)(salen)Co(III) complex [48,49,50]. The oxidation of bergamottin (**7**) is regioselective because, of the two double bonds, C17-C18 and C12-C13, present in the molecule, only the first one is involved in the oxidative process, probably because the C12-C13 double bond is nearby an electronegative oxygen atom and, being located in a position sterically hindered by the coumarin ring, undergoes unfavourable steric effects which contribute to make it scarcely reactive. 

## 3. Experimental

### 3.1. General Methods

Dichloromethane was dried by distillation over P_2_O_5_. Fourier transform IR (FTIR) spectra were obtained with a Perkin-Elmer Paragon 500 FT-IR spectrophotometer. ^1^H- and ^13^C-NMR spectra were recorded in CDCl_3_ on a Varian Unity Inova spectrometer at 500 and 125.7 MHz, respectively. The chemical shifts are given in ppm and referenced to residual CHCl_3_ (δ = 7.26) signal for ^1^H experiments and to the solvent signal (CDCl_3_, δ = 77.0) for ^13^C ones. ^1^H- and ^13^C-NMR assignments were supported by 2D (gCOSY, NOESY, HSQC, TOCSY) experiments. Electron impact mass spectra EIMS were recorded on a Kratos-MS 50 mass spectrometer with data system DS-90. High-performance liquid chromatography (HPLC) was performed on a Varian ProStar Solvent Delivery Module 230 apparatus equipped with a Varian ProStar 350 dual cell refractometer, using a semi-preparative Zorbax Sil (250 × 9.4 mm) column. The reactions were monitored by TLC and the components of the plates were visualized after spraying with 5% ammonium molybdate and 0.2% cerium sulfate in 10% sulfuric acid followed by heating. The reagents farnesyl acetate (**2**), α-ionone (**3**), β-ionone (**4**), methyl linolelaidate (**5**), methyl linolenate (**6**) and bergamottin (**7**) (commercial substances) were used as received. The methyl abietate (**1**) was obtained by esterification of abietic acid (commercial product Fluka, Milan, Italy) according to literature method [51]. Hydrogen peroxide (35%), pyridine and methyltrioxorhenium are commercial products (Aldrich, Milan, Italy).

### 3.2. General Oxidation Procedure

Pyridine (0.02 mmol, 12%) and 35% hydrogen peroxide (22 µL, 0.25 mmol, 1.5 equiv) were added to a solution of the MTO (0.008 mmol, 5%) in CH_2_Cl_2_ (2.5 mL) at 25 °C and this yellow mixture was stirred for 1 min. A solution of substrate (0.16 mmol, 1 equiv) in CH_2_Cl_2_ (1 mL) was added to this mixture and the stirring was continued for a suitable reaction time. The reaction mixture was dried over MgSO_4_ and the solvent was evaporated under reduced pressure. HPLC separation of the reaction mixture afforded desired products.

#### 3.2.1. Oxidation of Methyl Abietate (**1**)

The reaction mixture was kept under stirring for 30 min. Separation of the mixture was performed by HPLC, utilizing *n*-hexane/EtOAc 75:25 (v/v), (ɸ = 3.5 mL/min), to afford the three main compounds **8** (t_R_ = 8.1), **9** (t_R_ = 10.1), **10** (t_R_ = 11.6).

*1-Phenanthrenecarboxylic acid, 1,2,3,4,4a,4b,5,6,8a,9,10,10a-dodecahydro-1,4a-dimethyl-7-(1-methylethyl)-9-oxo-, methyl ester, [1R,(1α,4αβ,4βα,8αβ,10αα)]* (**8**). This compound has been identified on the basis of the comparison of its ^1^H- and ^13^C-NMR, EIMS, and IR data with those reported in the literature [30], gCOSY and NOESY confirm the proposed configuration (see ESI, S5-6).

*Phenanthro[1,2-b]oxirene-4-carboxylic acid, tetradecahydro-4,7a-dimethyl-9a-(1-methylethyl)-2-oxo-, methyl ester, [1aR-(1aα,1bα,3aβ,4β,7aα,7b β,9aα)]* (**9**). The structure of this compound was determined by ^1^H-NMR, APT, gCOSY, NOESY, TOCSY, HSQC, IR, EIMS (18eV), data. ^1^H-NMR: δ = 0.98 (d, *J* = 7 Hz, 3H, 16-H or 17-H), 0.94 (d, *J* = 7 Hz, 3H, 16-H or 17-H), 1.04 (s, 3H, 20-H), 1.07 (m, 1H, 1-Hax), 1.22 (s, 3H, 19-H), 1.56 (m, 1H, 11-Hax), 1,09 (m, 1H, 9-H), 1.58–1.67 (m, 3H, 2-Hax, 2-Heq, 3-Heq), 1.68–1.81 (m, 1H, 3-Hax), 1.80 (m, 1H, 1-Heq), 2.02 (m, 1H, 11-Heq), 2.02 (dd, *J* = 14.0, 1H, 3.0 Hz, 6-Heq), 1.08 (m, 1H, 12-Hax), 1.12 (m, 1H, 12-Heq), 2.16 (dd, *J* = 14.0, 3.0 Hz, 1H, 5-H), 1.53 (m, 1H, 15-H), 2.44 (t, *J* = 14.0 Hz, 1H, 6-Hax), 2.58 (d, *J* = 12.5 Hz, 1H, 8-H), 3.68 (s, 3H, 21-H), 3.67 (s, 1H, 14-H) ppm; ^13^C-NMR: δ = 14.1 (C-20), 16.1 (C-19), 18.0 (C-2), 17.9 (C-16 or C-17), 18.6 (C-17 or C-26), 24.3 (C-11), 18.5 (C-12), 34.5 (C-15), 36.6 (C-10), 37.0 (C-3), 37.4 (C-1), 41.5 (C-6), 47.5 (C-4), 49.1 (C-8), 49.8 (C-5), 52.5 (OCH3), 53.5 (C-9), 56.5 (C-14), 64.4 (C-13), 177.8 (C-18), 208.1 (C-7) ppm; IR (neat, cm^−1^): ν = 2952, 2857, 1725, 1715, 1455, 1437, 1385, 1243, 1097; EIMS (18 eV): *m/z* (%) = 348 (9) [M]^+^, 330 (100) [M−H_2_O]^+^, 305 (53) [M–C_3_H_7_]^+^, 270 (63) [330-CH_3_COOH].

*1-Phenanthrenecarboxylic acid, 1,2,3,4,4a,4b,5,6,7,9,10,10a-dodecahydro-7-hydroxy-1,4a-dimethyl-7-(1-methylethyl)-9-oxo-, methyl ester, [1R-([1R-(1α4αβ4βα7β10αα]* (**10**). The structure of this compound was determined by ^1^H-NMR, APT, gCOSY, NOESY, TOCSY, HSQC, IR, EIMS (18eV) data. ^1^H-NMR: δ = 1.01 (d, *J* = 7 Hz, 6H, 16-H e 17-H), 1.10 (s, 3H, 20-H), 1.13 (m, 1H, 1-Hax), 1.22 (s, 3H, 19-H), 1.58–1.67 (m, 4H, 2-Hax, 2-Heq, 3-Heq, 11-H), 1.71–1.73 (m, 2H, 3-Hax, 9-H), 1.95–1.98 (m, 3H, 1-Heq, 11-H, 12-H), 2.05–2.13 (m, 2H, 6-Heq, 12-H), 2.16–2.22 (m, 2H, 5-H, 15-H), 2.52 (t, *J* = 14 Hz, 1H, 6-Hax), 3.67 (s, 3H, 21-H), 5.26 (s, 1H, 14-H) ppm; ^13^C-NMR: δ = 15.4 (C-20), 17.0 (C-19), 17.7(C-2), 17.6 C-11), 21.2 (C-16 or C-17), 21.5 (C-17 or C-16), 24.4 (C-12), 35.5 (C-15), 36.8 (C-10), 38.6 (C-3), 38.7(C-6), 40.0 (C-1), 47.4 (C-4), 47.9 (C-5), 52.5 (c-21), 55.3 (C-9), 75.2 (C-13), 119.1 (C-14), 151.5 (C-8), 178.1 (C-18), 213.2 (C-7) ppm; IR (neat, cm^−1^): ν = 2926, 2859, 1734, 1718, 1538, 1253; EIMS (18 eV): *m/z* (%) = 348 (5) [M]^+^, 330 (81) [M^+^−H_2_O], 270 (45) [330–CH_3_COOH], 255(100) [270–CH_3_].

#### 3.2.2. Oxidation of Farnesyl Acetate (**2**)

The reaction mixture was kept under stirring for 30 min. HPLC separation with hexane/AcOEt (60:40 v/v, ɸ = 3.5 mL/min) afforded pure sample of the diepoxide **13** (t_R_ = 8.14 min) and a mixture of two compounds. HPLC separation of this mixture with *n*-hexane/EtOAc (90:10 v/v, ɸ = 3.5 mL/min) resulted in the isolation of the monoepoxide **11** (t_R_ = 13.26) and the monoepoxide **12** (t_R_ = 14.31).

*2-Penten-1-ol,3-methyl-5-[3-methyl-3-(4-methyl-3-penten-1-yl)-2-oxiranyl]-,1-acetate* (**11**). The structure of this compound was determined by ^1^H-NMR [35,36], and gCOSY and NOESY data. ^1^H-NMR: δ = 1.25 (s, 3H, 14-H), 1.43 (m, 1H, 8-H) 1.55 (s, 3H, 12-H or 13-H), 1.68 (s, 3H, 12-H or 13-H), 1.63–1.72 (m, 3H, 5,5',8'-H), 1.72 (s, 3H, 15-H), 2.05–2.21 (m, 4H, 4,4', 9,9'-H), 2.05 (s, 3H, CH_3_CO), 2.69 (t, *J* = 6.0 Hz, 1H, 6-H), 4.60 (d, *J* = 6.0 Hz, 2H, 1,1'-H), 5.07 (m, 1H, 10-H), 5.39 (m, 1H, 2-H) ppm.

*2,6-Nonadien-1-ol, 9-(3,3-dimethyloxiranyl)-3,7-dimethyl-acetate (2E,6E)* (**12**). The structure of this compound was determined by ^1^H-NMR, as reported in the literature [35,36,37] and supported by gCOSY and NOESY experiments.

*2-Dodecen-1-ol, 6,7,:10,11-diepoxy-3,7,11-trimethyl-acetate (mixture of diasteroisomers)* (**13**). The identification of this diepoxides mixture was determined by ^1^H-NMR, gCOSY and NOESY data. In the literature [35,36] the ^1^H-NMR spectrum of one of the possible diastereoisomers is reported. ^1^H-NMR: δ = 1.26 (s, 3H, CH_3_), 1.27 (s, 3H, CH_3_), 1.28 (s, 3H, CH_3_), 1.31 (s, 3H, CH_3_), 1.60 (m, 2H, 9,9'-H), 1.67 (m, 1H, 8-H), 1.69 (m, 2H, 5,5'-H), 1.72 (s, 3H, 15-H), 1.79 (m, 1H, 8'-H), 2.05 (s, 3H, CH_3_CO), 2.22 (m, 2H, 4,4'-H), 2,69 (m, 1H, 10-H), 2.75 (m, 1H, 6-H), 4.59 (d, *J* = 7.5 Hz, 2H, 1,1'-H), 5.38 (m, 1H, 2-H) ppm.

#### 3.2.3. Oxidation of α-Ionone (**3**)

The reaction mixture was kept under stirring for 18 h. Separation of the reaction mixture by HPLC, eluting with *n*-hexane/EtOAc (80:20 v/v, ɸ = 3.5 mL/min) leads to the formation of a racemic mixture of *cis*-4,5-epoxy-*α*-ionone (**14**, t_R_ = 12.0 min) and of a racemic mixture of *trans*-4,5-epoxy-*α*-ionone (**15**, t_R_ = 13.30 min). The two epoxides were identified on the basis of their spectroscopic data (^1^H-NMR and IR) as reported in the literature [39] and supported by gCOSY and NOESY experiments.

#### 3.2.4. Oxidation of β-Ionone (**4**)

The reaction mixture was kept under stirring for 20 h. Separation of the reaction mixture by HPLC, eluting with *n*-hexane/EtOAc (80:20 v/v, ɸ = 3.5 mL/min) afforded pure sample of the (±)-5,6-epoxy-β-ionone (**16**, t_R_ = 7.3 min). The (±)-5,6-epoxy-β-ionone has been identified on the basis of its spectroscopic data (^1^H-NMR and IR) which are identical to those reported in the literature [40] and supported by gCOSY and NOESY experiments.

#### 3.2.5. Oxidation of Methyl Linolelaidate (**5**)

The reaction mixture was kept under stirring for 8 h. HPLC separation with *n*-hexane/EtOAc (75:25 v/v, ɸ = 3.5 mL/min) afforded a mixture of two compounds (t_R_ = 4.36 min) and an inseparable mixture of diastereomeric bisepoxides **19** (t_R_ = 6.53 min). The mixture of two compounds was separate by HPLC (*n*-hexane/EtOAc 90:10 v/v, ɸ = 3.5 mL/min) to yield the monoepoxide **17** (t_R_ = 7.03 min) and the monoepoxide **18** (t_R_ = 7.84 min).

*9-Undecenoic acid, 11-(3-pentyloxiranyl) methyl ester* (**17**). This compound has been identified on the basis of its ^1^H- and ^13^C-NMR data, supported by gCOSY and NOESY experiments and EIMS. ^1^H-NMR: δ = 0.89 (3H, m, CH_3_), 1.31 (12H, m, 4,5,6,7,16,17-H ), 1.41 (2H, m, 15-H), 1.52 (2H, m, 14-H), 1.62 (2H, m, 3-H), 1.99 (2H, q, *J* = 7.0 Hz, 8-H), 2.19 (1H, m, 11-H), 2.25 (1H, m, 11-CH), 2.30 (2H, t, *J* = 7.5 Hz, 2-H), 2.69 (2H, m, 12 and 13-H), 3.67 (3H, s, OCH_3_), 5.40 (1H, m, 10-H), 5.52 (1H, m, 9-H); ^13^C-NMR: δ 14.0, 22.6, 24.9, 25.7, 28.9, 29.1, 29.3, 31.6, 31.9, 32.6, 34.1, 35.2, 51.4, 58.2, 58.5, 124.4, 133.5, 174.3; EIMS (20 eV): *m/z* (%) = 310 (2) [M]^+^, 292 (5) [M−18]^+^, 279 (4) [M−31]^+^, 207 (17) [O≡CCH_2_CH=CH(CH_2_)_7_COOCH_3_-18]^+^, 164 (51) [M–CH_3_(CH_2_)_4_CH(O)CHCH_3_–18]^+^, 147 (42) [M–CH_3_(CH_2_)_4_CH(O)CH_2_–31–18]^+^.

*Oxiraneoctanoic acid, 3-(2-octenyl)-methyl ester* (**18**). This compound has been identified on the basis of its ^1^H- and ^13^C-NMR data, supported by gCOSY and NOESY experiments and EIMS. ^1^H-NMR: δ = 0.88 (3H, t, *J* = 7.5 Hz, CH_3_), 1.31 (12H, m, 4,5,6,7,16,17-H), 1.42 (2H, m, 15-H), 2.0 (2H, m, 14-H), 1.62 (2H, m, 3-H), 1.51 (2H, m, 8-H), 2.19 (1H, m, 11-H), 2.25 (1H, m, 11'-H), 2.30 (2H, t, *J* = 8 Hz, 2-H), 2.69 (2H, m, 9 and 10-H), 3.67 (3H, s, OCH_3_), 5.40 (1H, m, 12-H), 5.53 (1H, m, 13-H); ^13^C-NMR: δ 14.0, 22.5, 24.9, 25.9, 29.02, 29.04, 29.1, 29.2, 31.4, 31.9, 32.6, 34.1, 35.2, 51.4, 58.2, 58.4, 124.3, 133.7, 174.3; EIMS (20 eV): *m/z* (%) = 292 (1) [M−18]^+^, 279 (2) [M−31]^+^, 200 (3) [CH_2_(O)CH(CH_2_)_7_COOCH_3_]^+^, 125 (11) [M CH_3_(CH_2_)_4_CH=CHCH_2_(O)CH_2_–31]^+^, 109 (19) [CH_3_(CH_2_)_6_COOCH_3_-49]^+^.

*Methyl 9,10-12,13-diepoxyoctadecenoate* (**19**). The mixture of diastereomeric bisepoxides has been identified by ^1^H data supported by gCOSY and NOESY experiments and EIMS. ^1^H-NMR: δ = 0.88 (3H, t, *J* = 7 Hz, CH_3_), 1.24–1.38 (14H, m, 4,5,6,7,15,16,17-H), 1.52 (4H, m, 8 and 14-H), 1.61 (2H, m, 3-H), 1.72–1.78 (2H, m, 11-H), 1.90–1.96 (2H, m, 11-H), 2.28 (2H, t, *J* = 7.5 Hz, 2-H), 2.68–2.76 (2H, m, 9 and 13-H), 2.75–2.82 (2H, m, 10 and 12-H), 3.65 (3H, s, OCH_3_); EIMS (20 eV): *m/z* = 308 [M-18]^+^, 295[M−31]^+^, 277[M−49]^+^, 251 [CH_2_CH(O)CHCH_2_CH(O)CH(CH_2_)_7_COOCH_3_–18]^+^, 237 [CH_2_CH(O)CH CH_2_CH(O)CH(CH_2_)_7_COOCH_3_–32]^+^, 223 [O≡CCHCH_2_CH(O)CH(CH_2_)_7_COOCH_3_–31]^+^, 211 [O=CHCH_2_ CH(O)CH(CH_2_)_7_COOCH_3_–31]^+^, 165 [CH_3_CH(O)CH(CH_2_)_7_COOCH_3_–31–18]^+^, 137 [O=CH(CH_2_)_7_COO CH_3_-31-18]^+^, 109 [CH_3_(CH_2_)_6_COOCH_3_–49]^+^.

#### 3.2.6. Oxidation of Methyl Linolenate (**6**)

The reaction mixture was kept under stirring for 8 h. HPLC separation by *n*-hexane/EtOAc 75:25 (v/v, ɸ = 3.5 mL/min) afforded two mixtures of products. The first mixture was separated by *n*-hexane/EtOAc 95:5 (v/v, ɸ = 3.5 mL/min) to yield the monoepoxides **20** (methyl *cis*-12,13-epoxy-9*Z*,15*Z*-octadecadienoate) (t_R_ = 17.86 min), **21** (methyl *cis*-15,16-epoxy-9*Z*,12*Z*-octadecadienoate) (t_R_ = 23.08 min) and **22** (methyl *cis*-9,10-epoxy-12*Z*,15*Z*-octadecadienoate) (t_R_ = 25.38 min). The three epoxides were identified on the basis of their spectroscopic data (^1^H-, and ^13^C-NMR, EIMS) [43,44] and supported by gCOSY and NOESY experiments. The second mixture was separated with hexane/AcOEt (80: 20 v/v, ɸ = 3.5 ml/min) affording two fractions (A and B) which are both inseparable mixtures of two bisepoxides (A: t_R_ = 10.59 and t_R_ = 10.84; B: t_R_ = 11.86 and t_R_ = 12.14 min).

#### 3.2.7. Oxidation of Bergamottin (**7**)

The reaction mixture was kept under stirring at room temperature for 4 h. Then was dried over molecular sieves (3 Å) for three hours and the solvent was evaporated under reduced pressure. The residue was purified by silica gel chromatography (*n*-hexane/EtOAc 85:15) to afford the racemic mixture **23** (17-epoxybergamottin) (t_R_ = 17.54 min and t_R_ = 20.77 min as determined by HPLC analysis on a chiral stationary phase column, Chiralcel OD-H *n*-hexane/EtOAc 9:1). The racemic mixture of epoxides has been identified on the basis of their spectroscopic data (^1^H- and ^13^C-NMR) [47].

#### 3.2.8. Representative Procedures for the HKR of Terminal Epoxides

The (*S*,*S*)salen Co(II) (see ESI, S2) (4.6 × 10^−4^ mmol in 20 µL of THF) was treated with racemic 17,18-epoxy bergamottin (9.9 × 10^−2^ mmol) in 200 µL of THF and 2.1 × 10^−3^ mmol of AcOH. To this solution, cooled to 0 °C, 5.5 × 10^−2^ mmol of H_2_O were added. The solution was allowed to warm to room temperature and stirred for 28 h. The reaction mixture was dried over molecular sieves (3 Å) and the solvent was evaporated under reduced pressure. PLC separation with *n*-hexane/EtOAc 1:1 afforded a pure sample of 17(*R*)-18 DHB (yield 5%) identified on the basis of its ^1^H-NMR data [47]. The presence of a doublet at δ = 3.31 ppm is attributed to the H-17 of the (*R*)-enantiomer, while the same proton for the (*S*)-enantiomer is at 3.22 ppm.

## 4. Conclusions

We have oxidized some natural compounds, containing conjugate double bonds with the versatile oxidant system MTO/H_2_O_2_/pyridine. Some reactions have shown very good regio- and stereoselectivity. Stereoelectronic and steric effects and nucleophilicity of double bonds control the selectivity. Under the experimental conditions adopted in this work, the oxidation of methyl abietate leads to the oxidation of the double bond of the ring B with formation of the ketone **8**, while the remaining oxidation products **9** and **10** are secondary oxidation products derived from the further oxidation of **8**. α- and β-ionones are very selectively converted into the corresponding epoxides, whereas oxidation of methyl linolelaidate and methyl linolenate yields mixtures of mono- and diepoxides. Furthermore, we have developed a synthetic strategy leading to the 17(*R*),18-dihydroxybergamottin by HKR of the racemic epoxide obtained by a very regioselective epoxidation of bergamottin with MTO/H_2_O_2_/pyridine. Some of the oxidation products obtained are relevant as synthons in the biomimetic synthesis of cyclic terpenoids (farnesyl derivatives), in perfumery and fragrance industry (α- and β-ionone epoxides) or in medical implications (17(*R*),18-dihydroxybergamottin).

## Supplementary Materials

Supplementary materials can be accessed at: http://www.mdpi.com/1420-3049/18/11/13754/s1.
