# Supplementary material for: Selective Oxidation Reactions of Natural Compounds with Hydrogen Peroxide Mediated by Methyltrioxorhenium
Source: Molecules. 2013 Nov 7;18(11):13754–68. doi: 10.3390/molecules181113754 (PMC6270049; doi:10.3390/molecules181113754)

Supplementary Materials

Preparation of catalyst [(S,S)-(+)N,N'-bis(3,5-di-tert-butylsalicylidene)-1,2-cyclohexanediaminato (2-)] cobalt(II)

The catalyst was prepared from the commercially available ligand [(*S,S*)-(+)N,N'-bis(3,5-di-*tert*-butylsalicylidene)-1,2-cyclohexanediamine: a solution of cobalt(II) acetate (98.78 mg, 0.56 mmol) in EtOH (4.5 mL) was added to a solution of ligand (301.9 mg, 0.55 mmol) in toluene (4.5 mL). A brick-red solid began to precipitate before addition was complete. The mixture was refluxed for 1.5 h. Precipitated solid was isolated by vacuum filtration and recrystallized from $CHCl_3$/n-hexane. This compound was identified by ESI-MS.

The Co(II) complex is catalytically inactive, however, and it must be subjected to one-electron oxidation to produce a (salen)Co(III)X complex (X anionic ligand) prior to the HKR. This may be done conveniently by aerobic oxidation in the presence of a mild Brønsted acid. Water alone was found not to mediate the oxidation reaction, but a screen of additives revealed that acetic acid was effective and that the corresponding Co(III) precatalyst is convenient for use in HKR reactions both in terms of its preparation and reactivity [1–3].

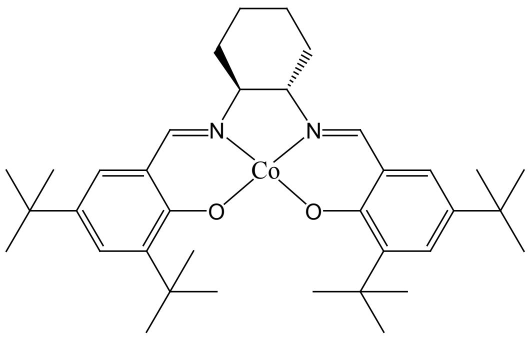

1. Schaus, S.E.; Brandes, B.D.; Larrow, J.F.; Tokunaga, M.; Hansen, K.B.; Gould, A.E.; Furrow, M.E.; Jacobsen, E.N. Highly Selective Hydrolytic Kinetic Resolution of Terminal Epoxides Catalyzed by Chiral (salen)CoIII Complexes. Practical Synthesis of Enantioenriched Terminal Epoxides and 1,2-Diols. *J. Am. Chem. Soc.* **2002**, *124*, 1307–1315.
2. Larrow, J.F.; Jacobsen, E.N. Asymmetric Processes Catalyzed by Chiral (Salen)Metal Complexes. *Top. Organomet. Chem.* **2004**, *6*, 123–152.
3. Nielsen, L.P.C.; Stevenson, C.P.; Blackmond, D.G.; Jacobsen, E.N. Mechanistic Investigation Leads to a Synthetic Improvement in the Hydrolytic Kinetic Resolution of Terminal Epoxides. *J. Am. Chem. Soc.* **2004**, *126*, 1360–1362.

NMR Spectra

Figure S1. ^{1}H-NMR spectrum of **8** in $CDCl_3$.

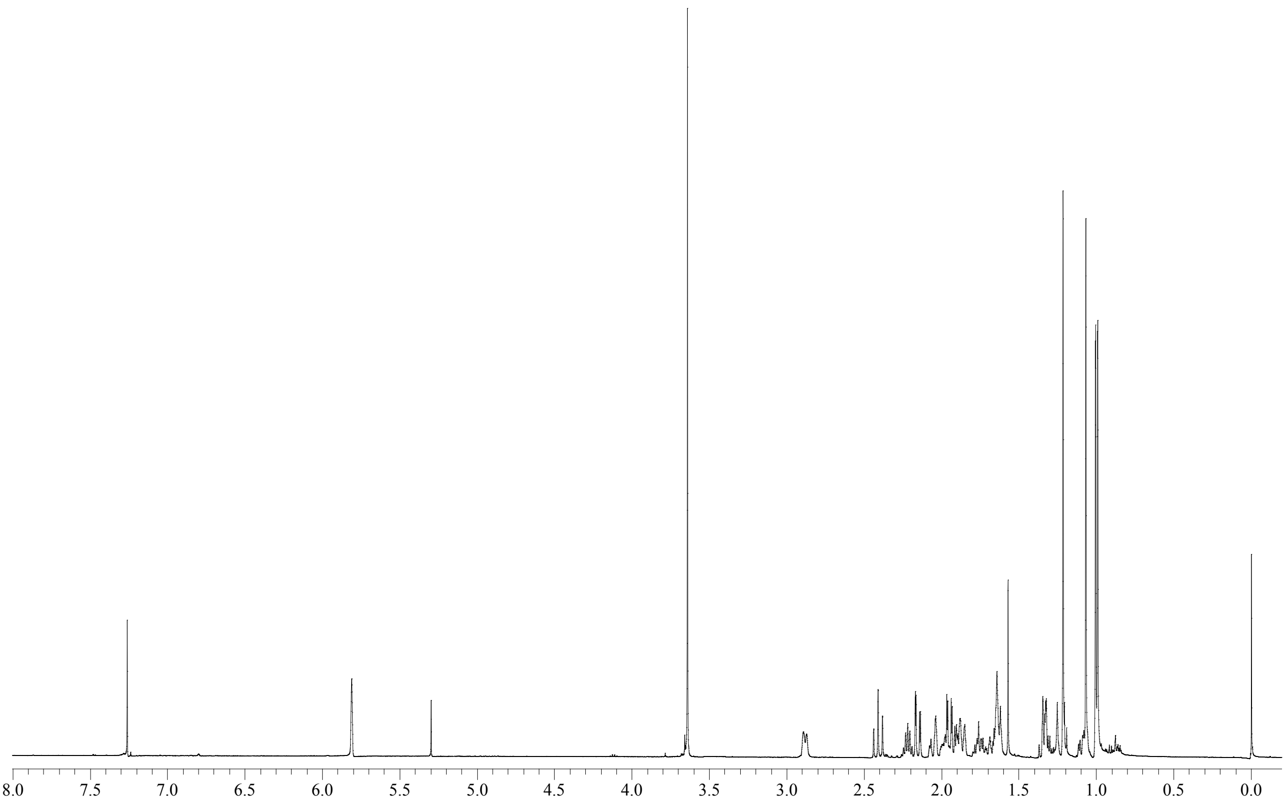

Figure S2. ^{13}C-NMR spectrum of **8** in $CDCl_3$.

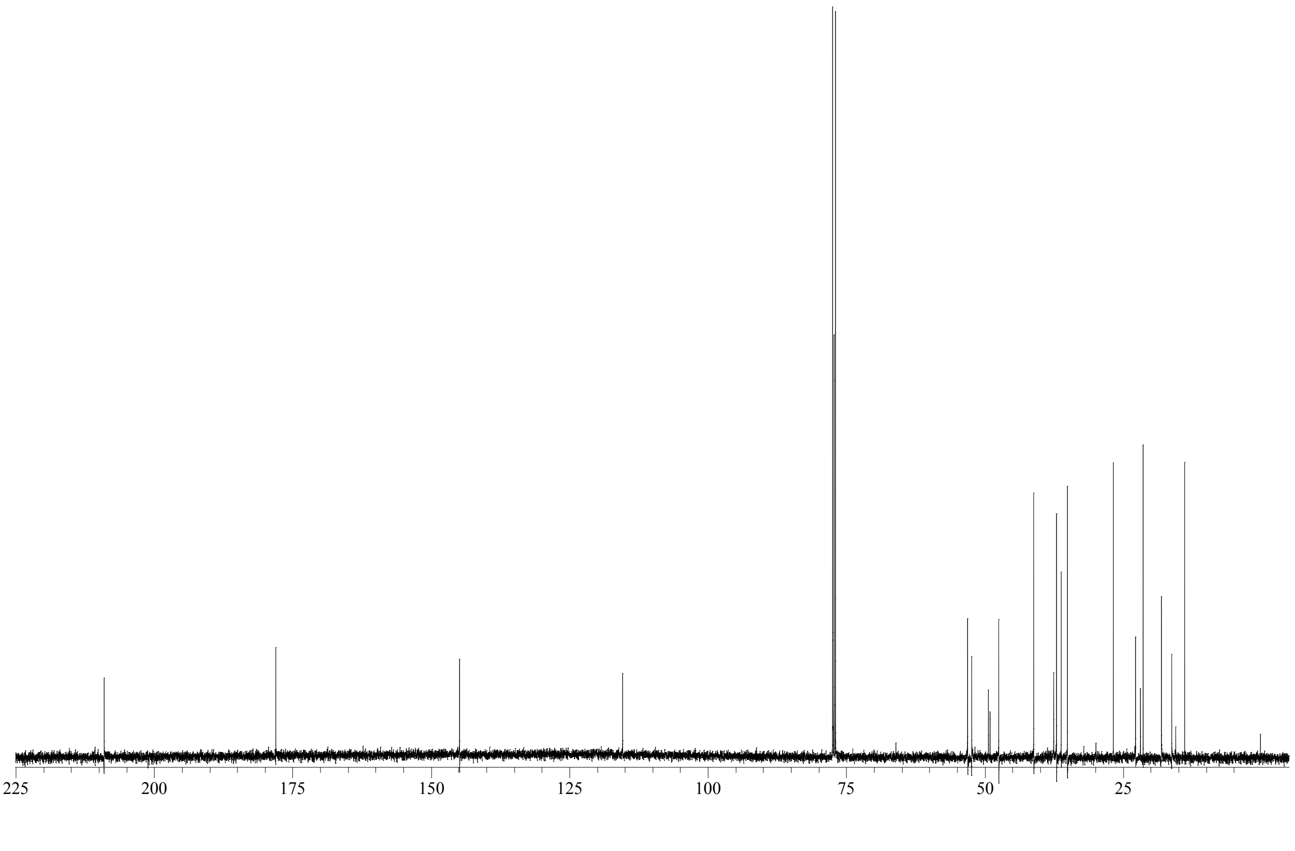

Figure S3. gCOSY spectrum of **8** in $CDCl_3$.

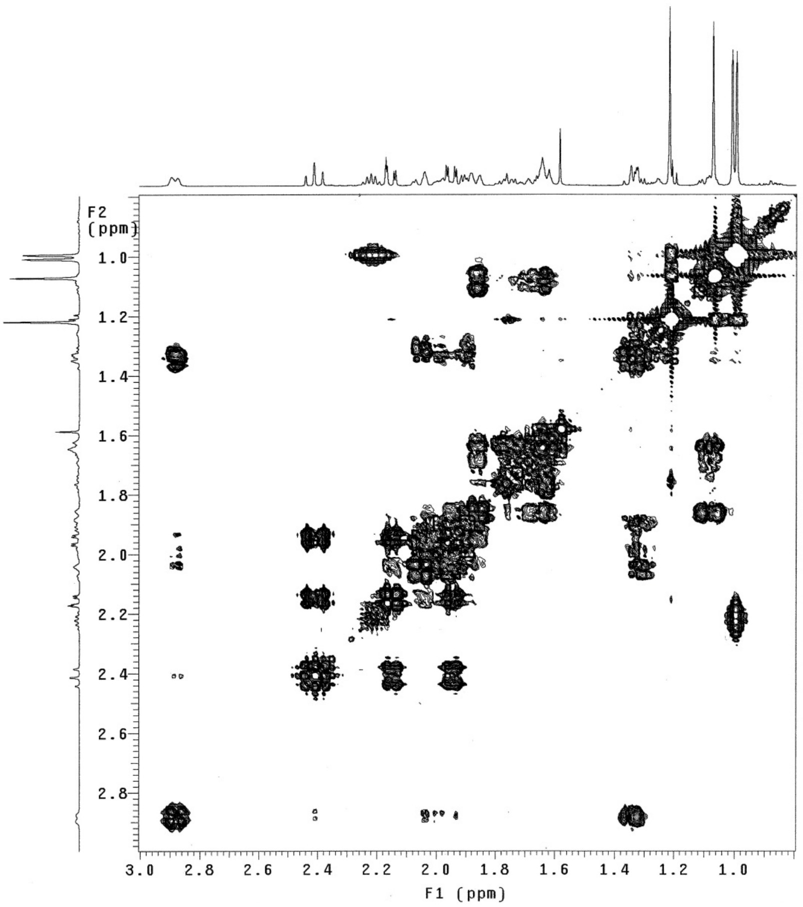

Figure S4. NOESY spectrum of **8** in $CDCl_3$.

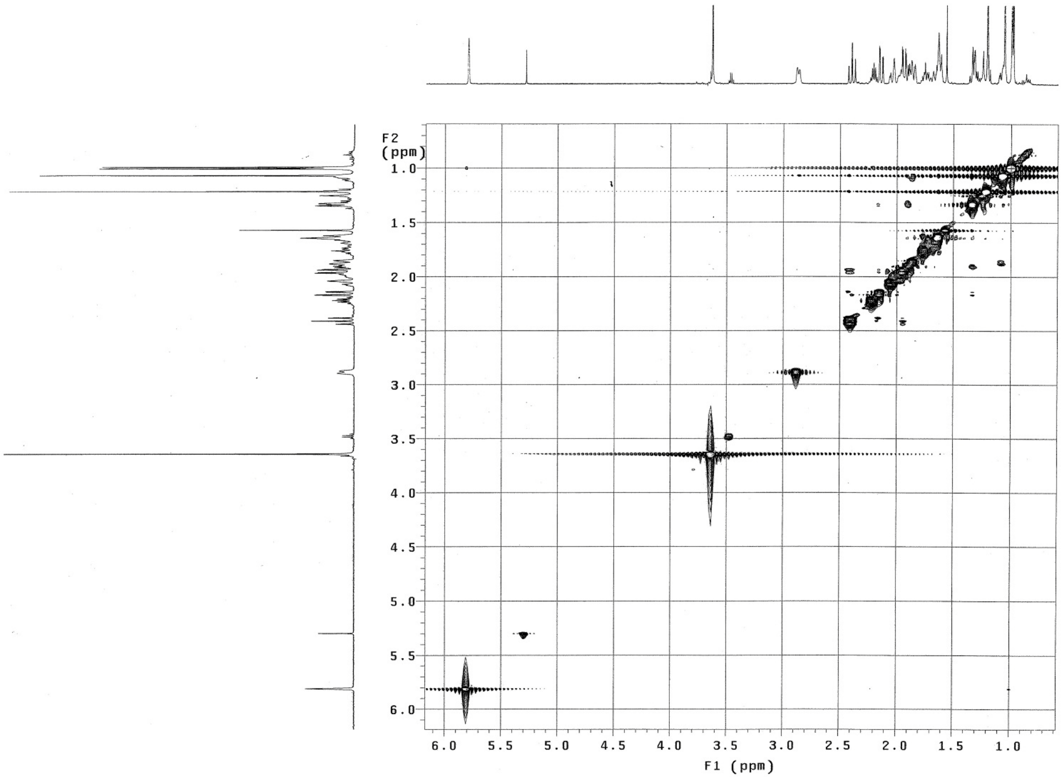

Figure S5. HSQC spectrum of **8** in $CDCl_3$.

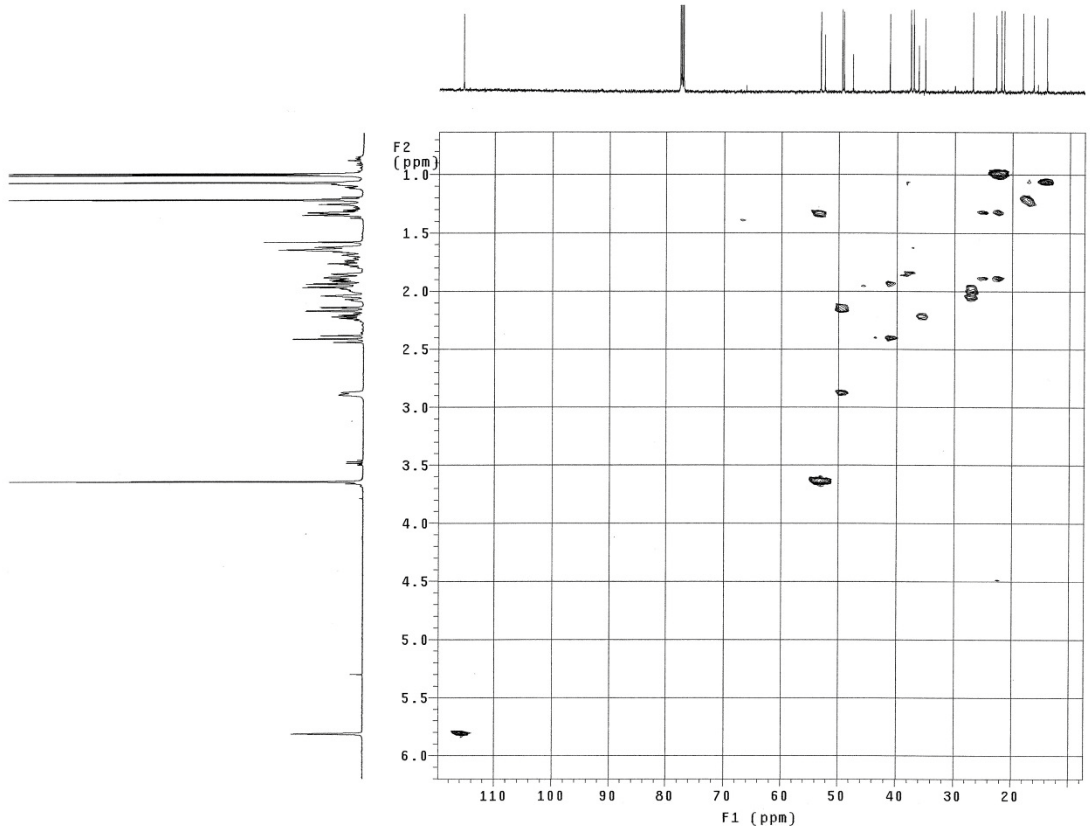

Figure S6. ^1H-NMR spectrum of **9** in $CDCl_3$.

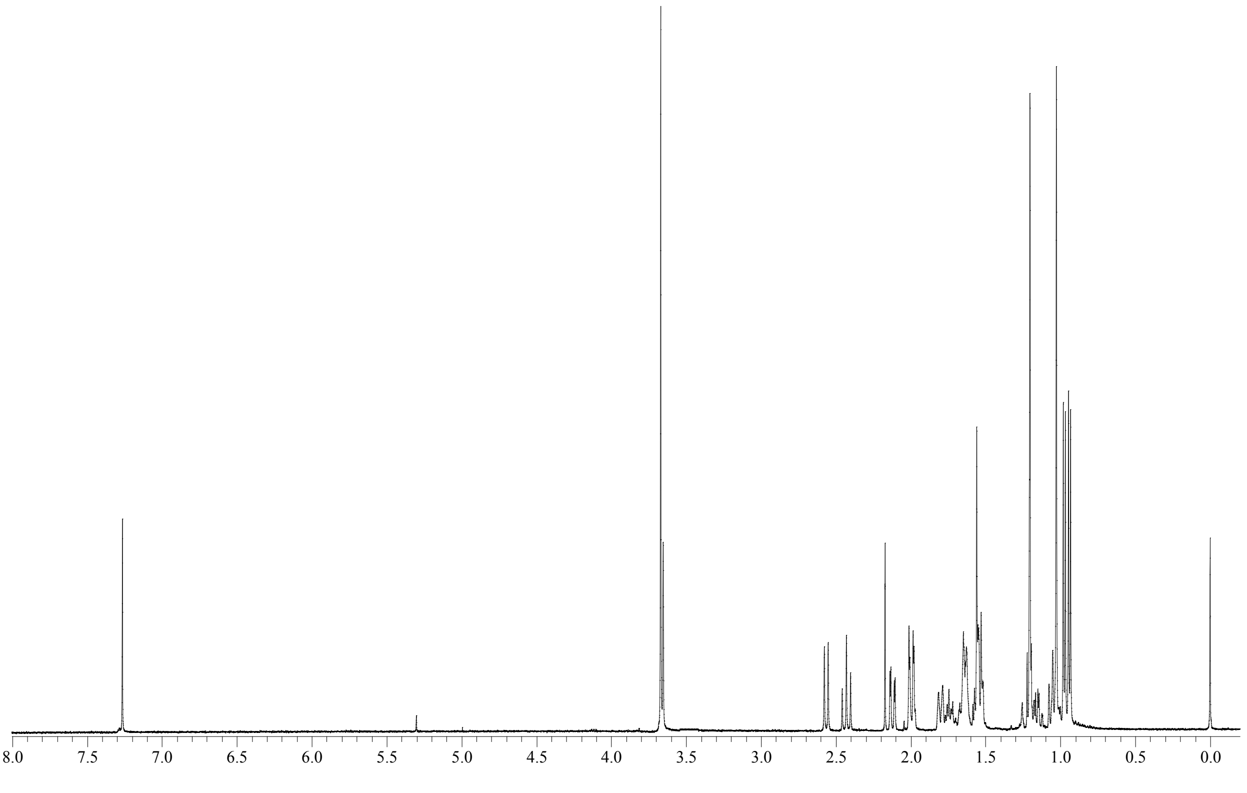

Figure S7. APT spectrum of **9** in $CDCl_3$.

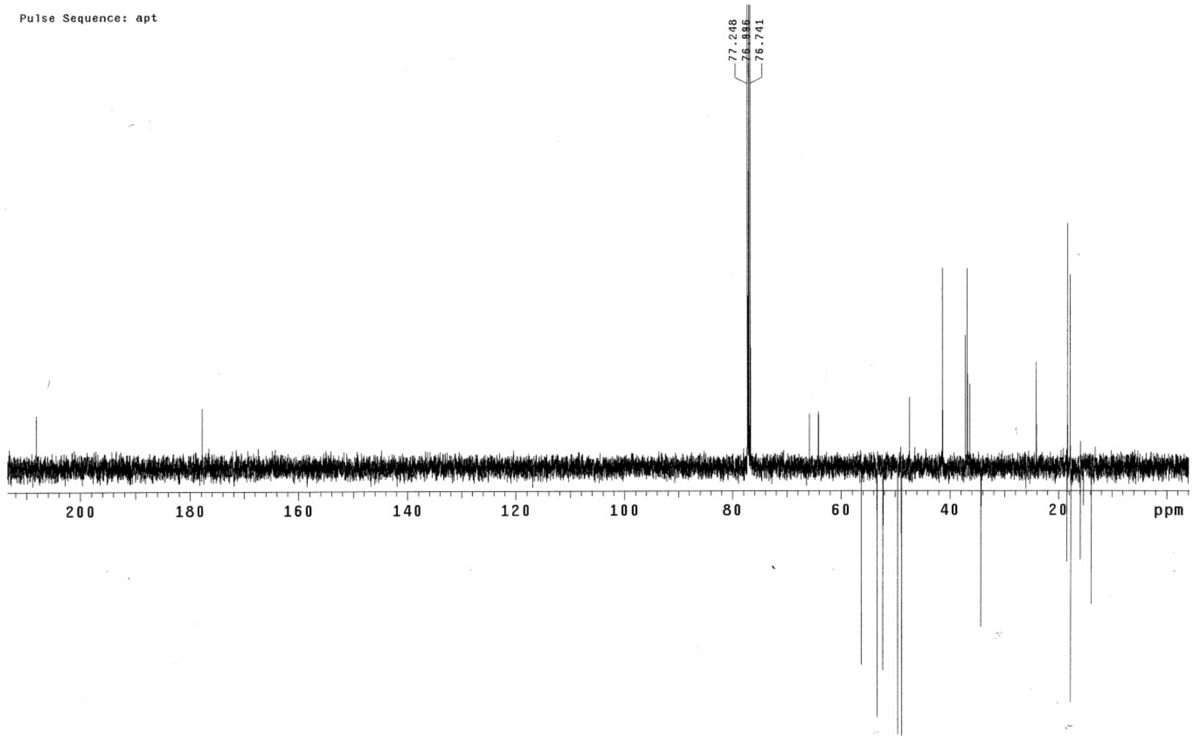

Figure S8. gCOSY spectrum of **9** in $CDCl_3$.

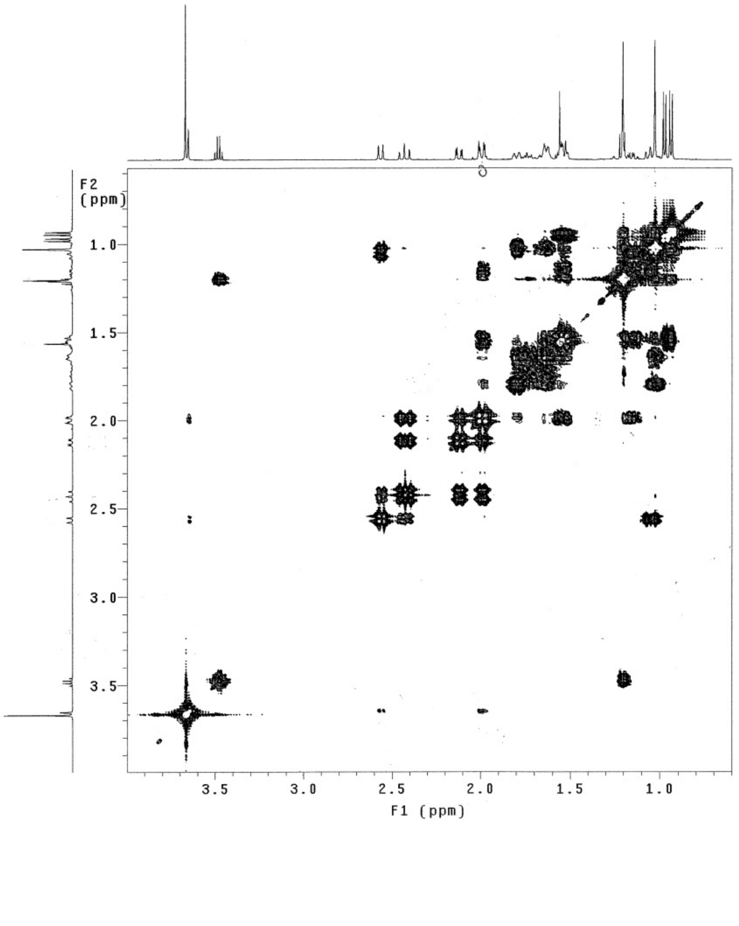

Figure S9. NOESY spectrum of **9** in $CDCl_3$.

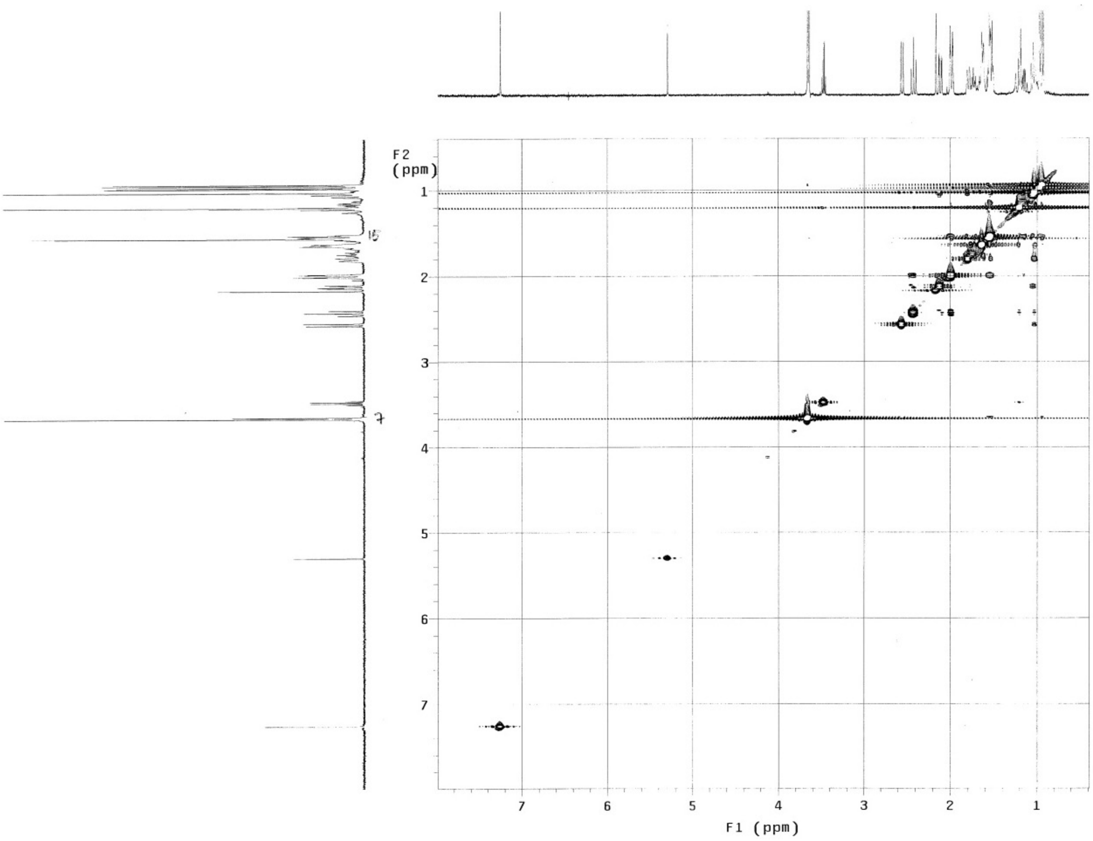

Figure S10. TOCSY spectrum of **9** in $CDCl_3$.

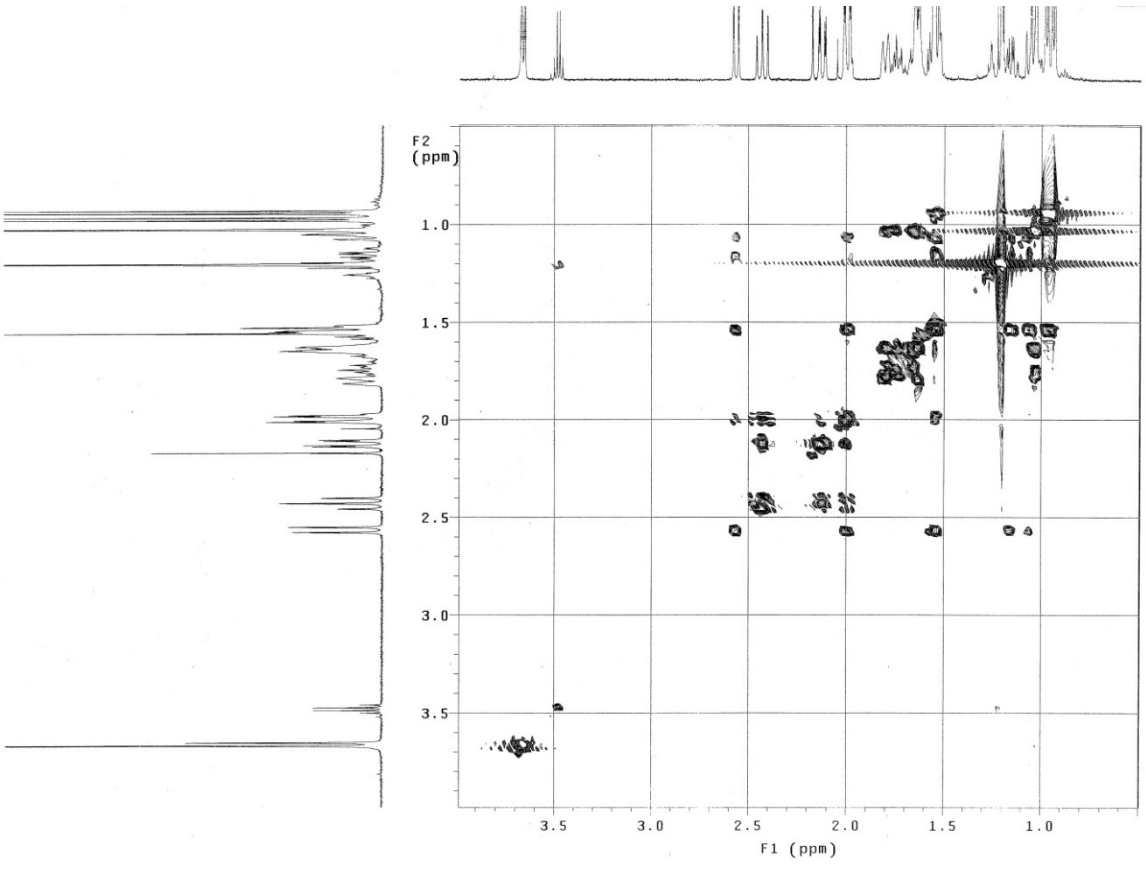

Figure S11. HSQC spectrum of **9** in $CDCl_3$.

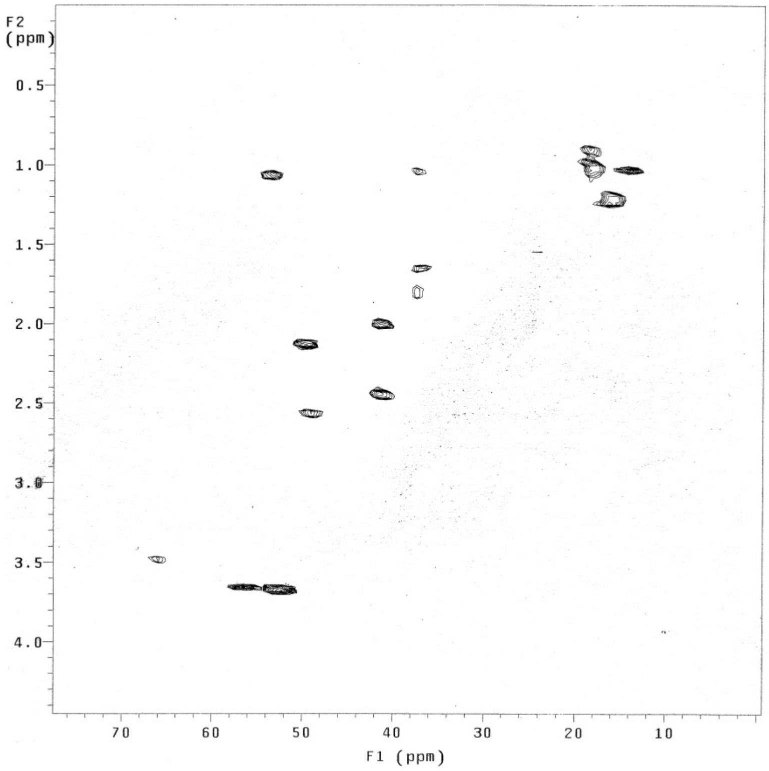

Figure S12. ^{1}H-NMR spectrum of **10** in $CDCl_3$.

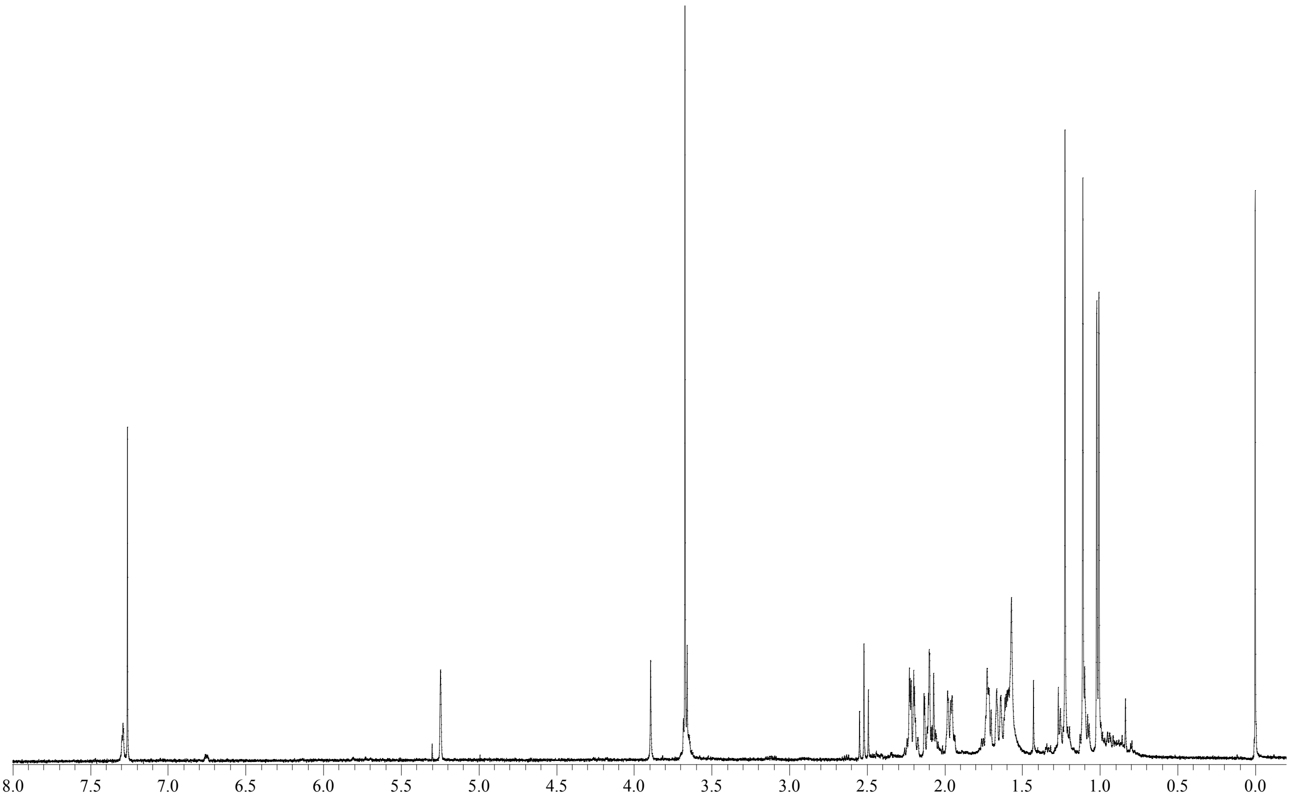

Figure S13. APT spectrum of **10** in $CDCl_3$.

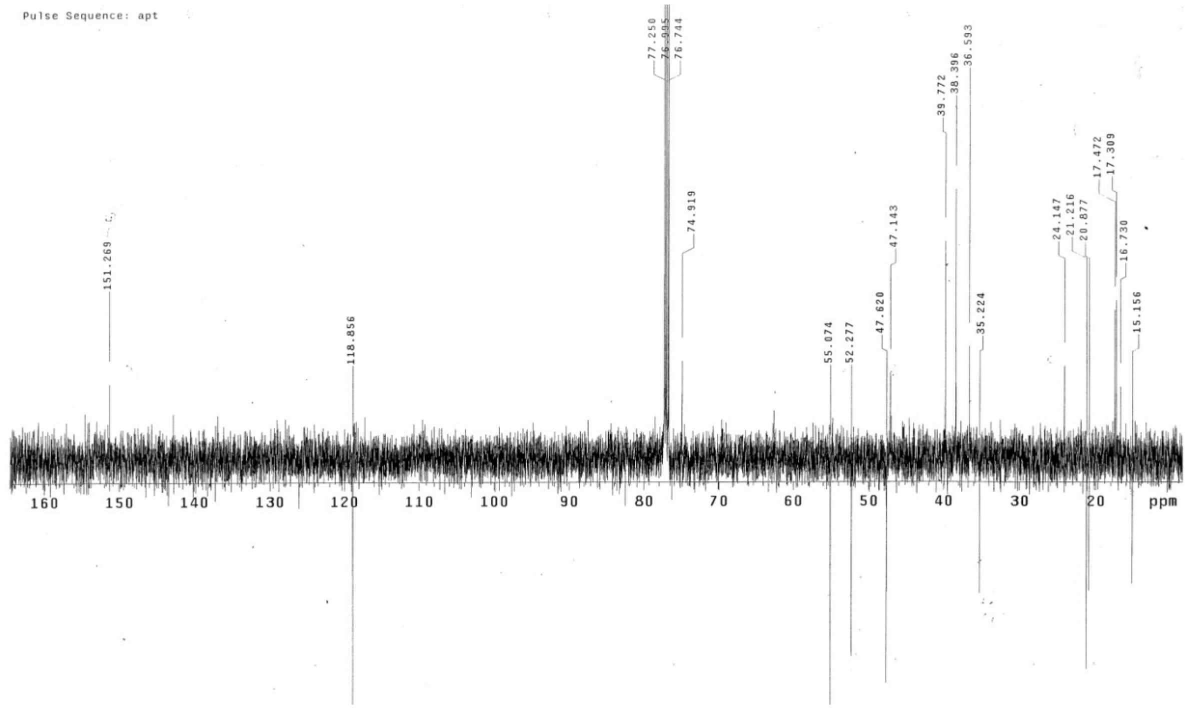

Figure S14. gCOSY spectrum of **10** in $CDCl_3$.

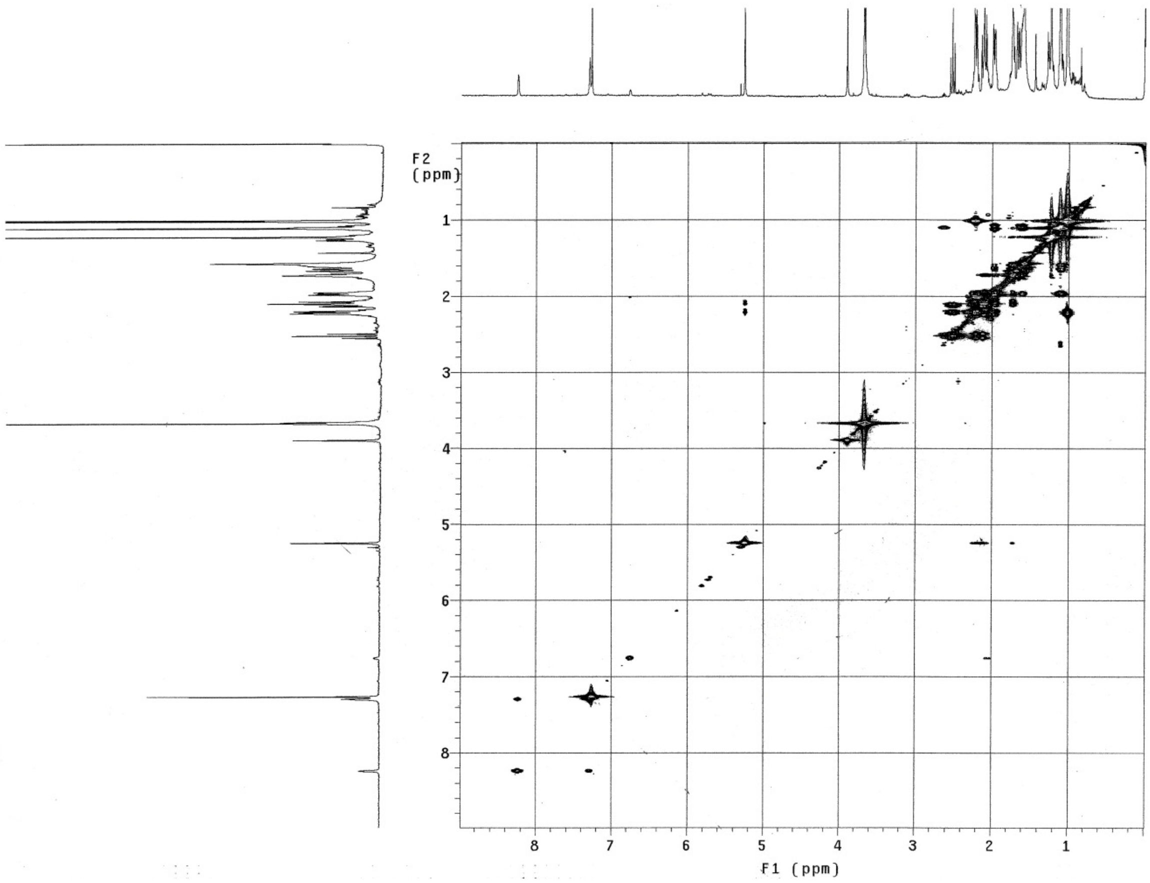

Figure S15. NOESY spectrum of **10** in $CDCl_3$.

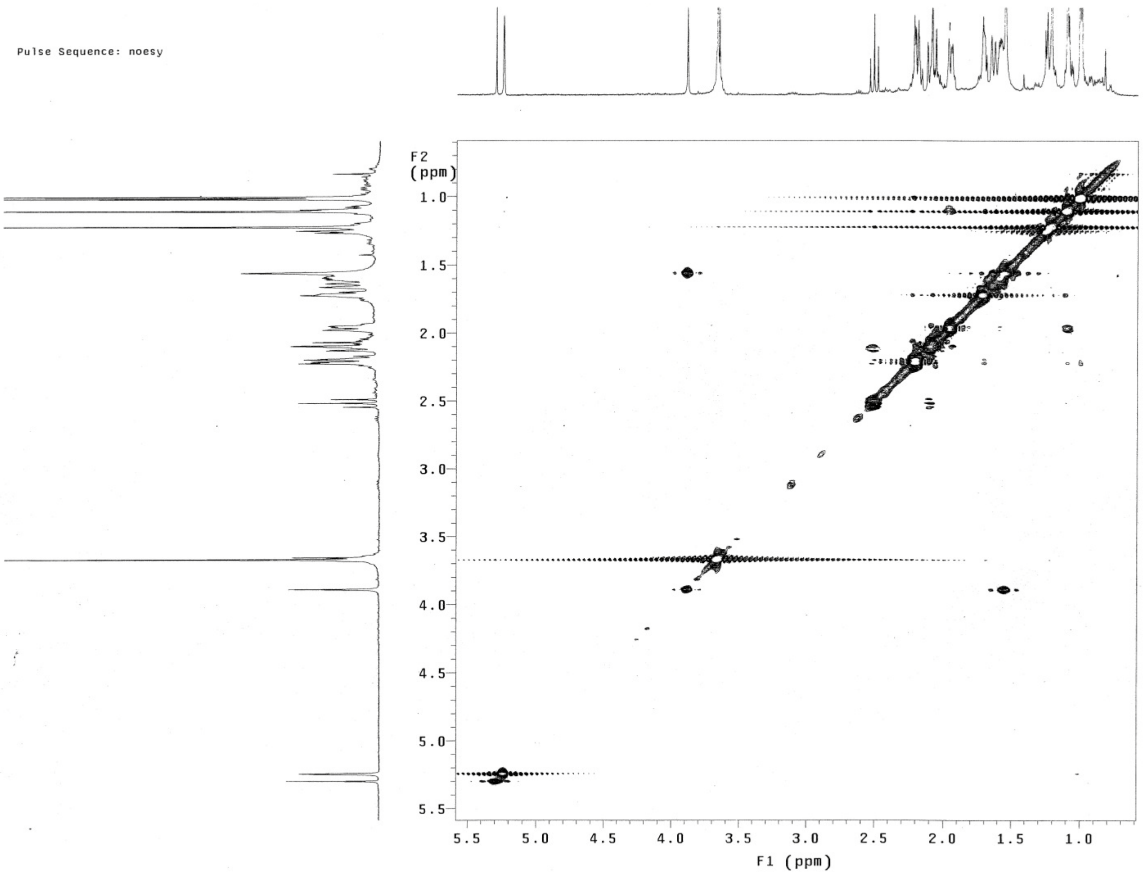

Figure S16. TOCSY spectrum of **10** in $CDCl_3$.

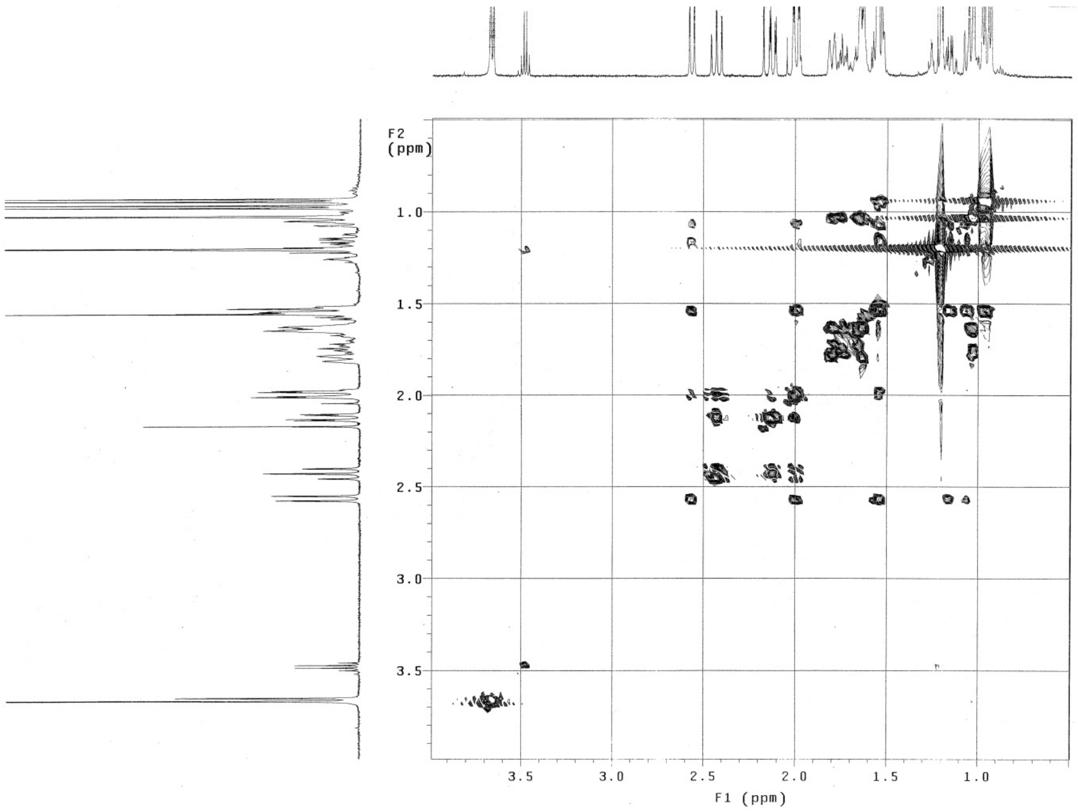

Figure S17. HSQC spectrum of **10** in $CDCl_3$.

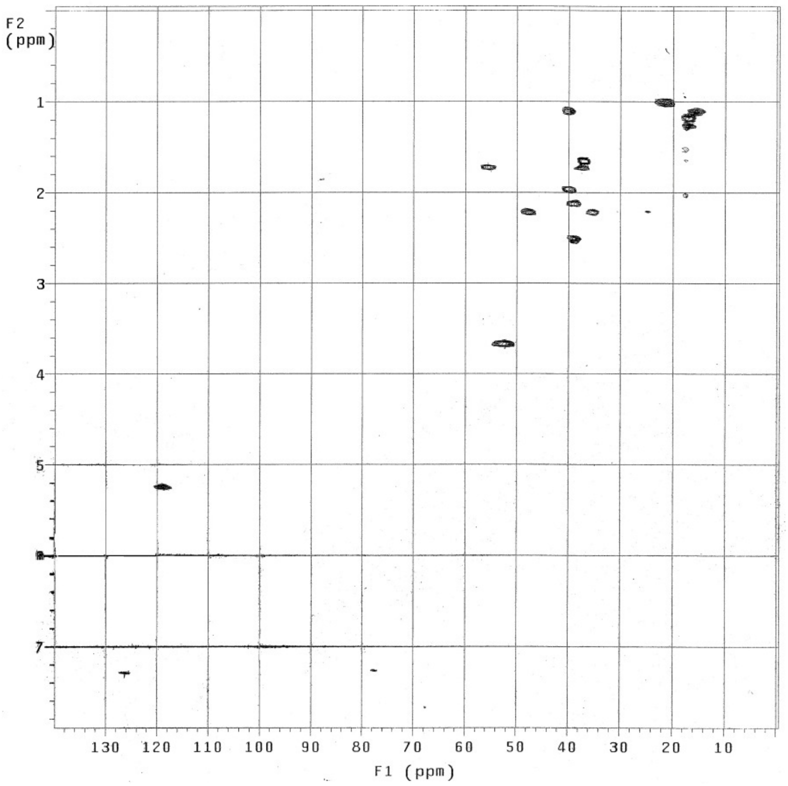

Figure S18. ^{1}H-NMR spectrum of **11** in $CDCl_3$.

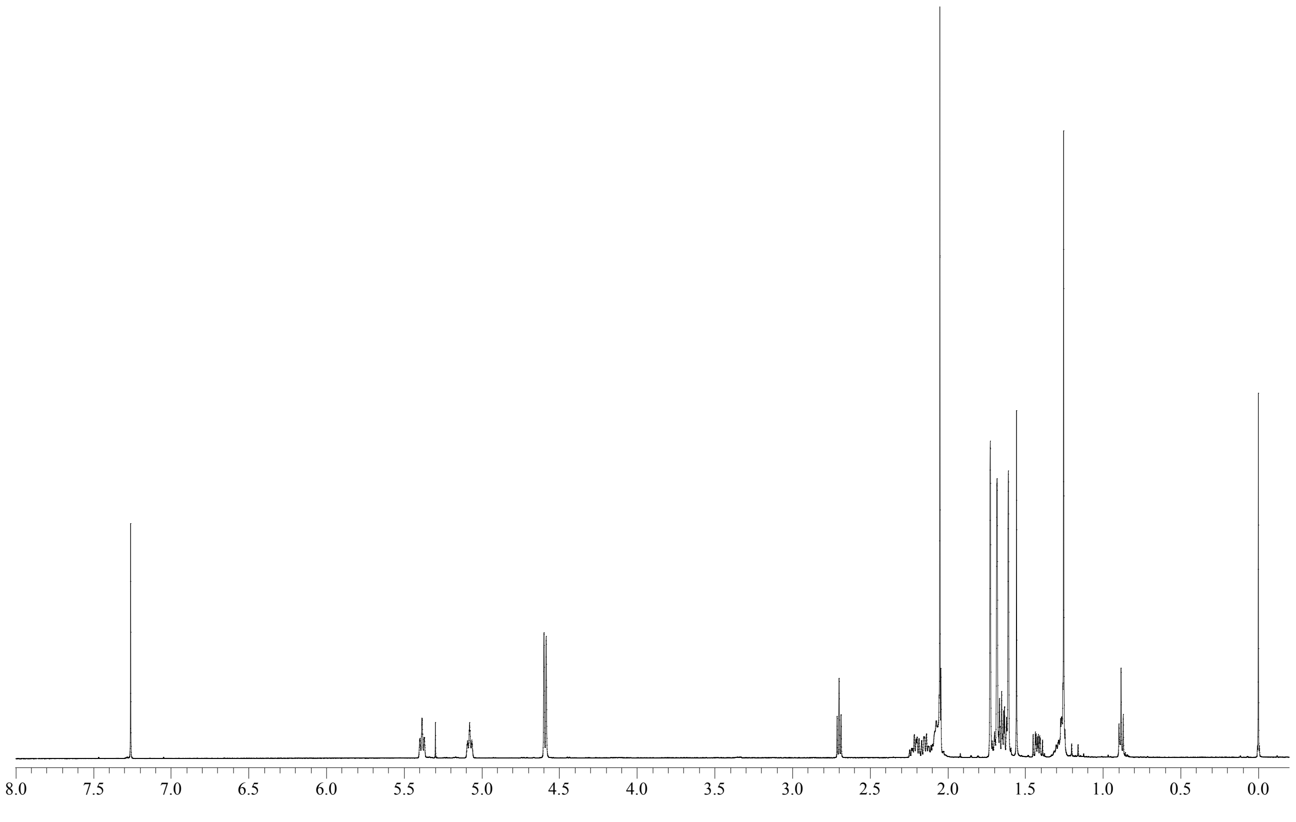

Figure S19. gCOSY spectrum of **11** in $CDCl_3$.

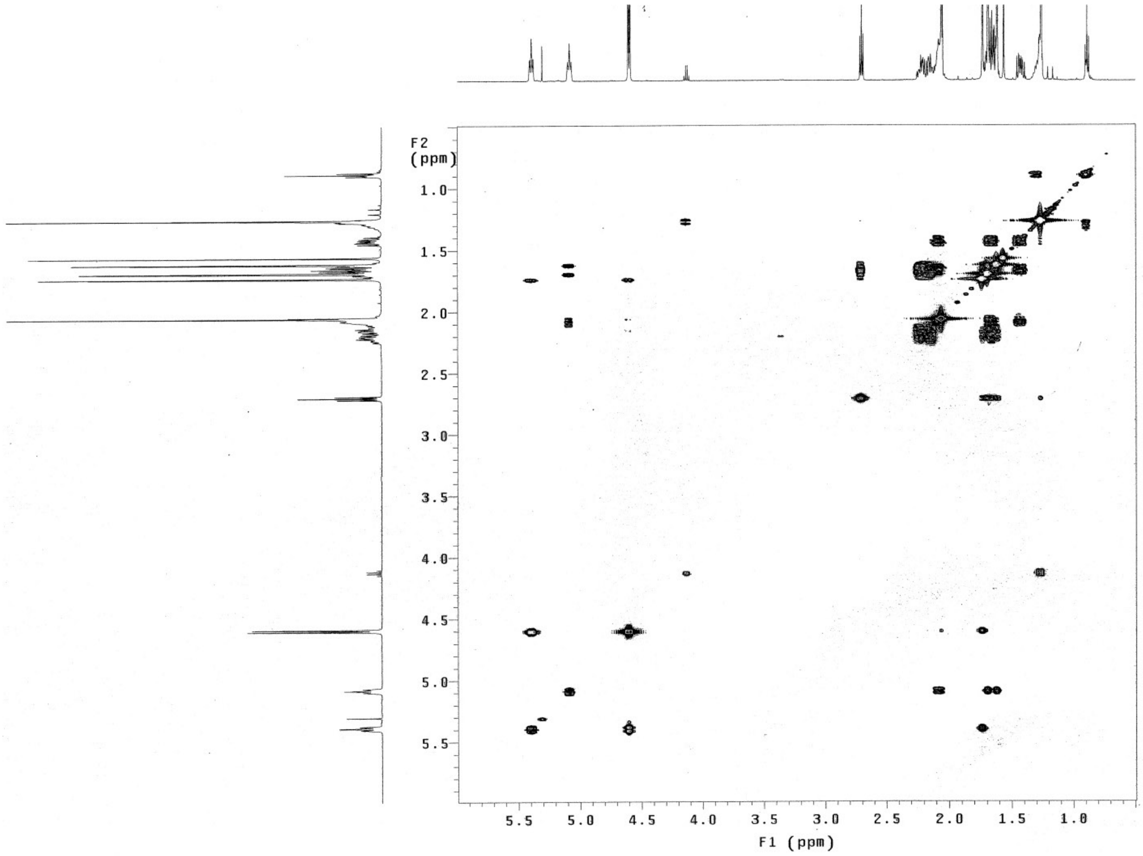

Figure S20. NOESY spectrum of **11** in $CDCl_3$.

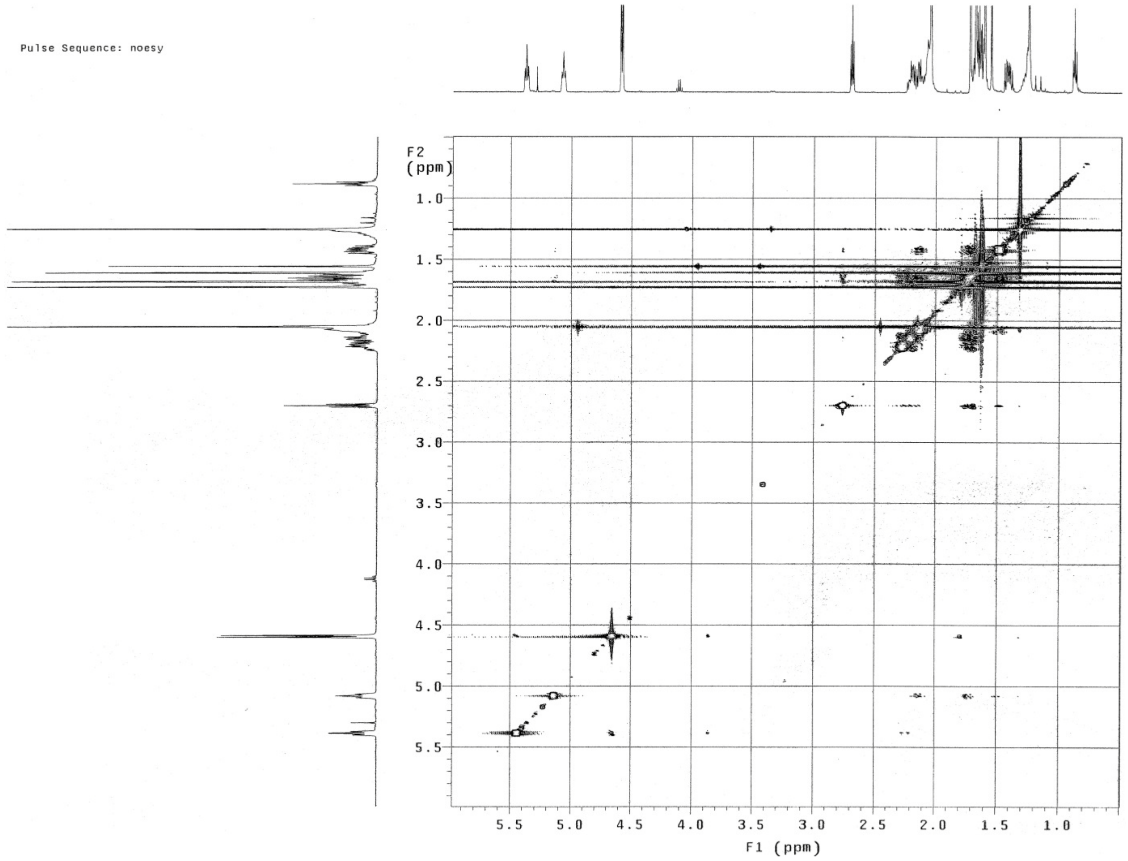

Figure S21. ^{1}H-NMR spectrum of **12** in $CDCl_3$.

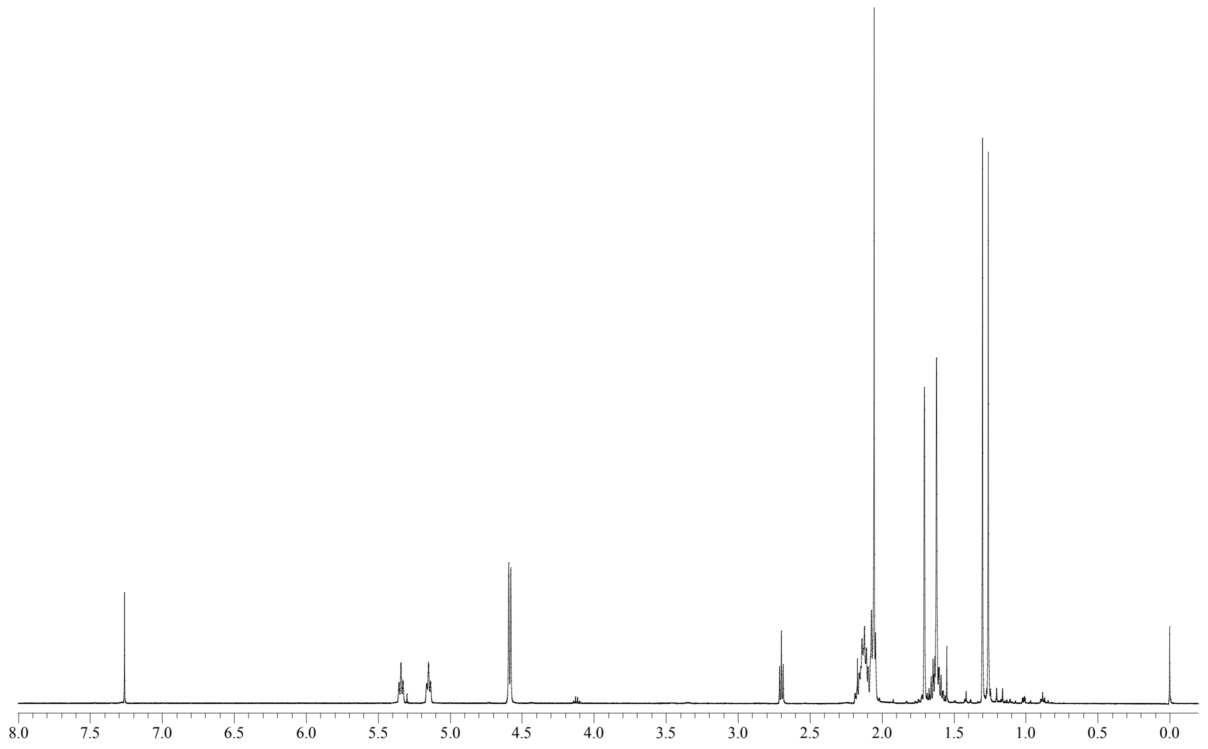

Figure S22. gCOSY spectrum of **12** in $CDCl_3$.

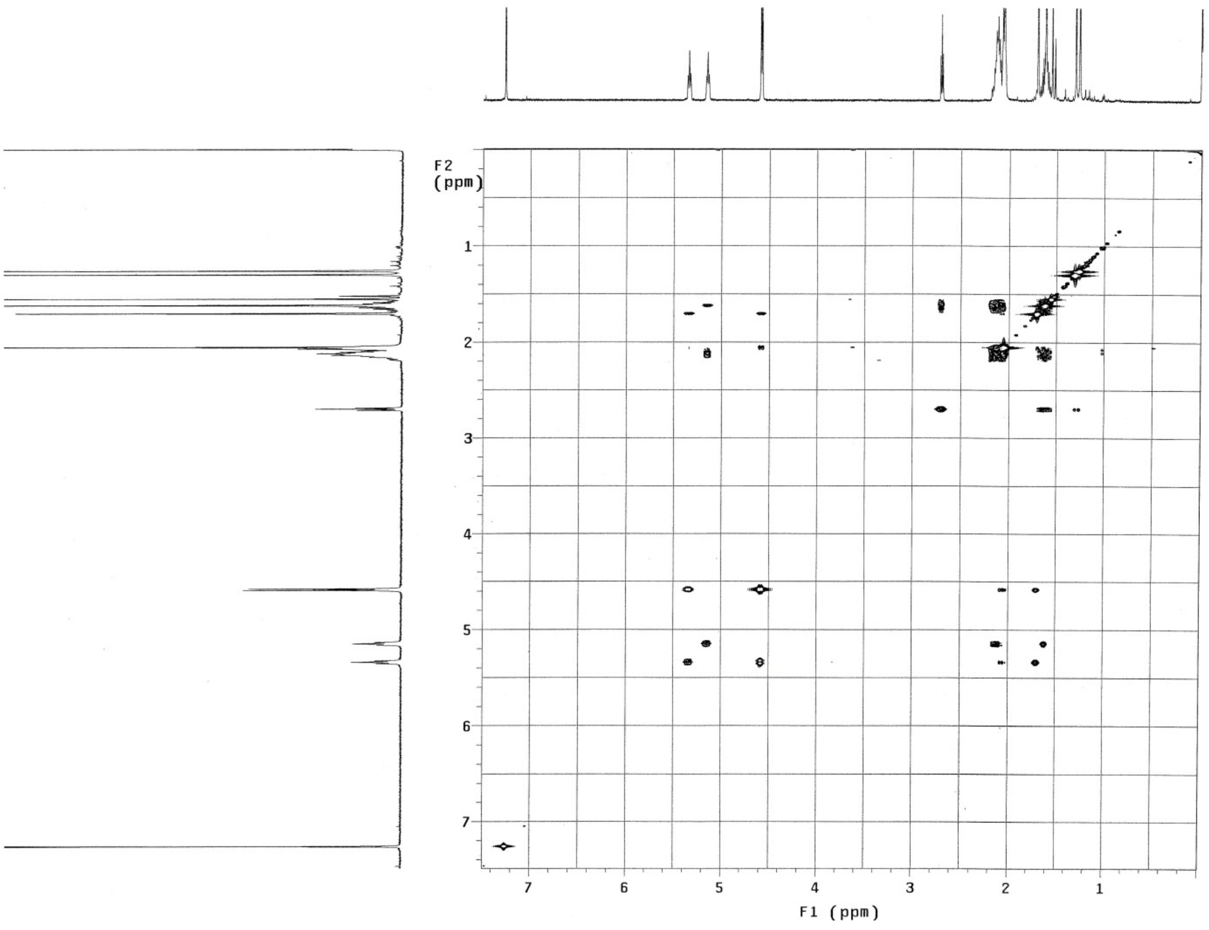

Figure S23. NOESY spectrum of **12** in $CDCl_3$.

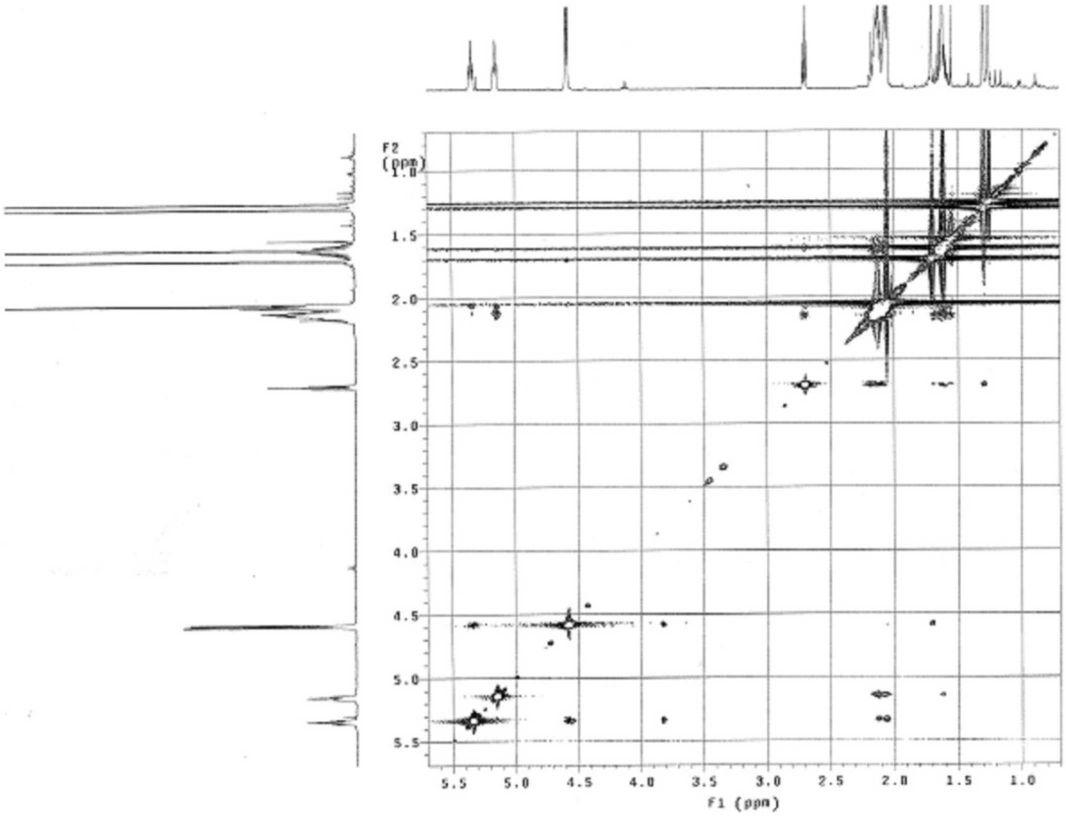

Figure S24. ^{1}H-NMR spectrum of **13** in $CDCl_3$.

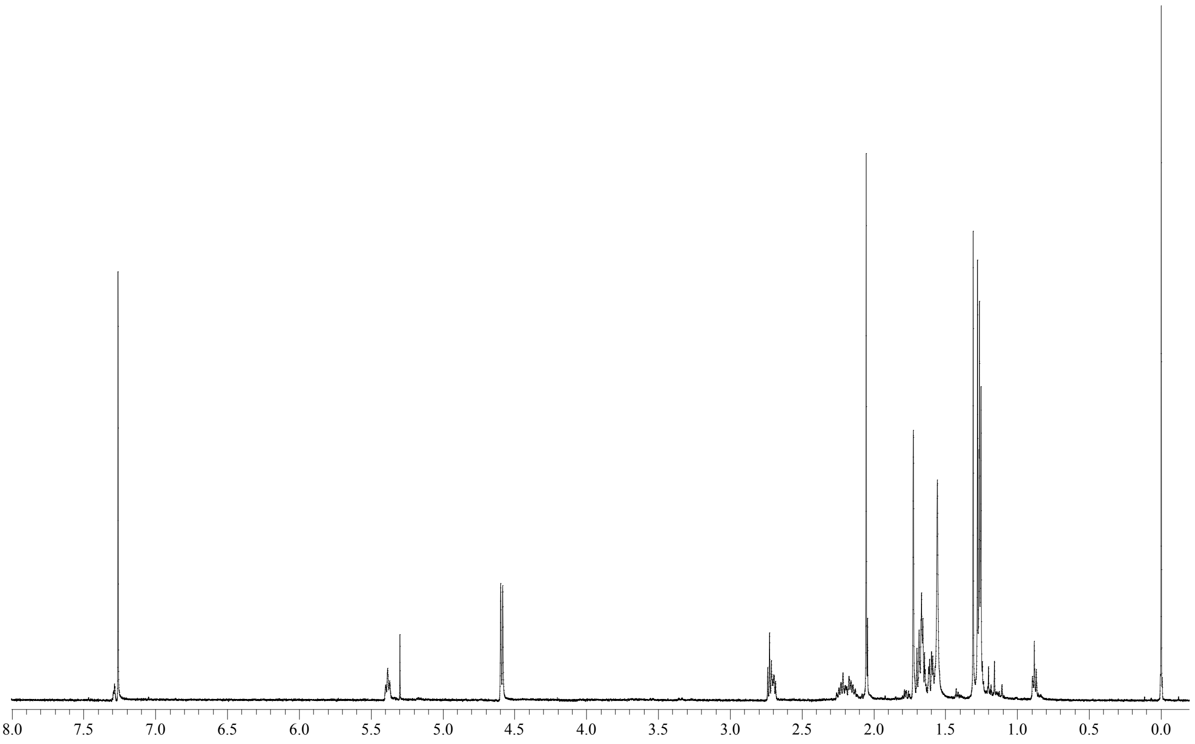

Figure S24b. Detail of ^{1}H-NMR spectrum of **13** in $CDCl_3$.

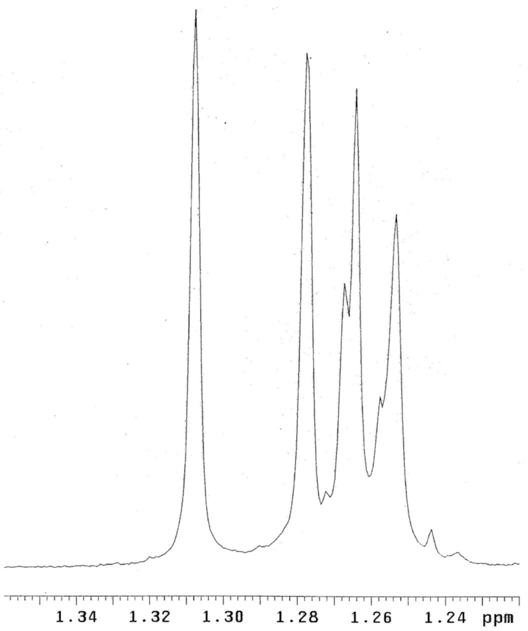

Figure S25. gCOSY spectrum of **13** in $CDCl_3$.

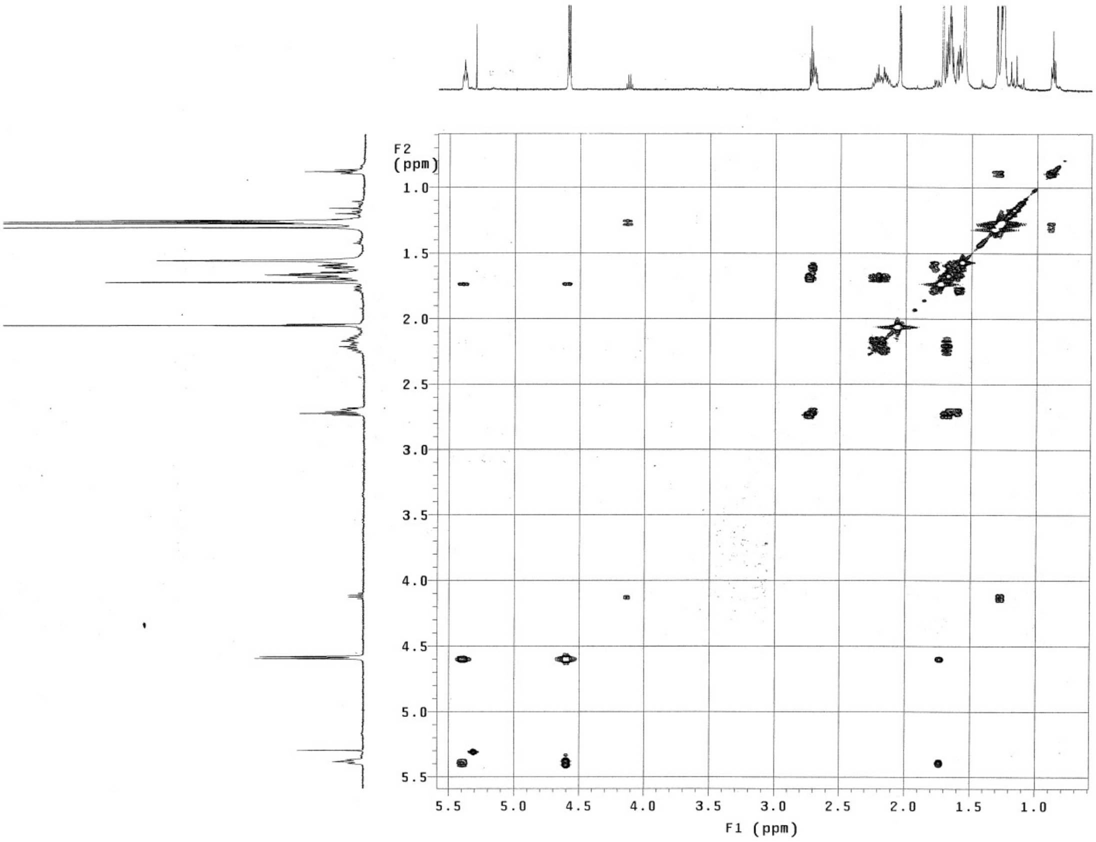

Figure S26. NOESY spectrum of **13** in $CDCl_3$.

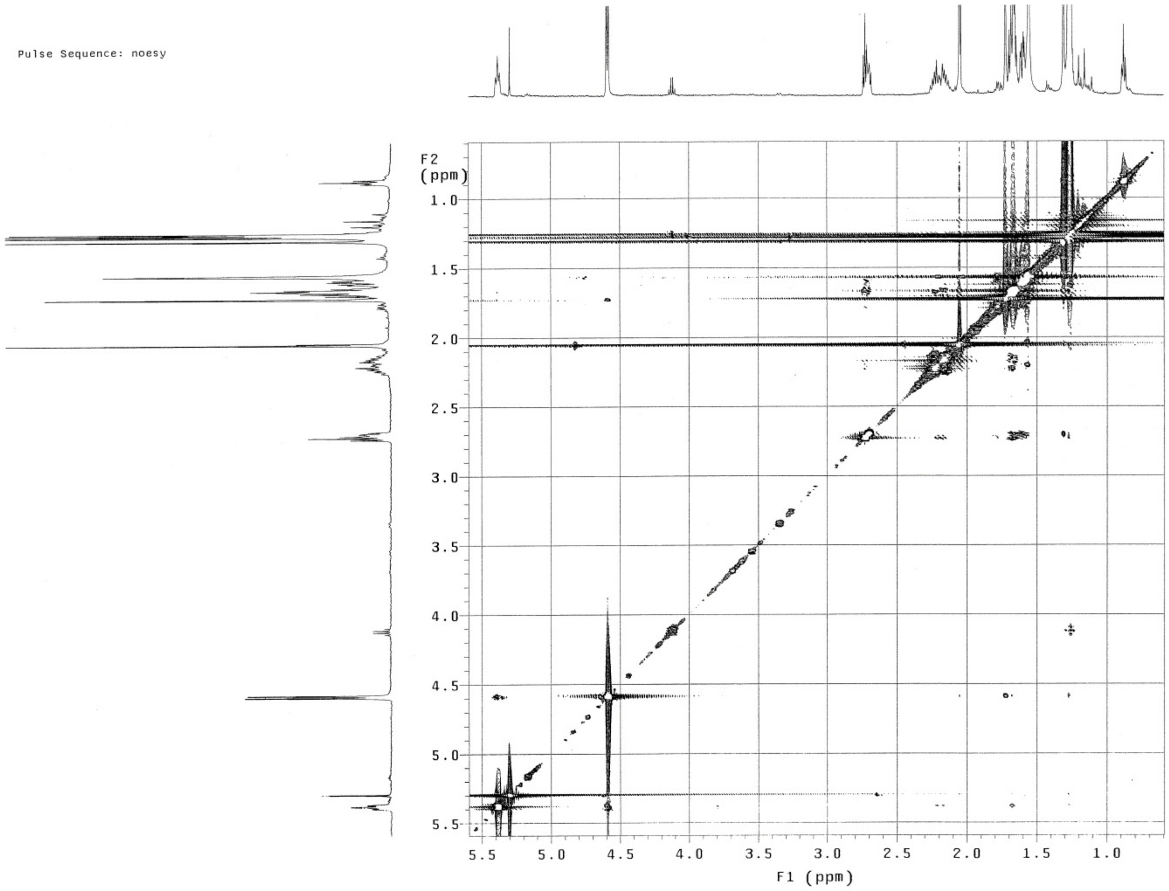

Figure S27. ^{1}H-NMR spectrum of **14** in $CDCl_3$.

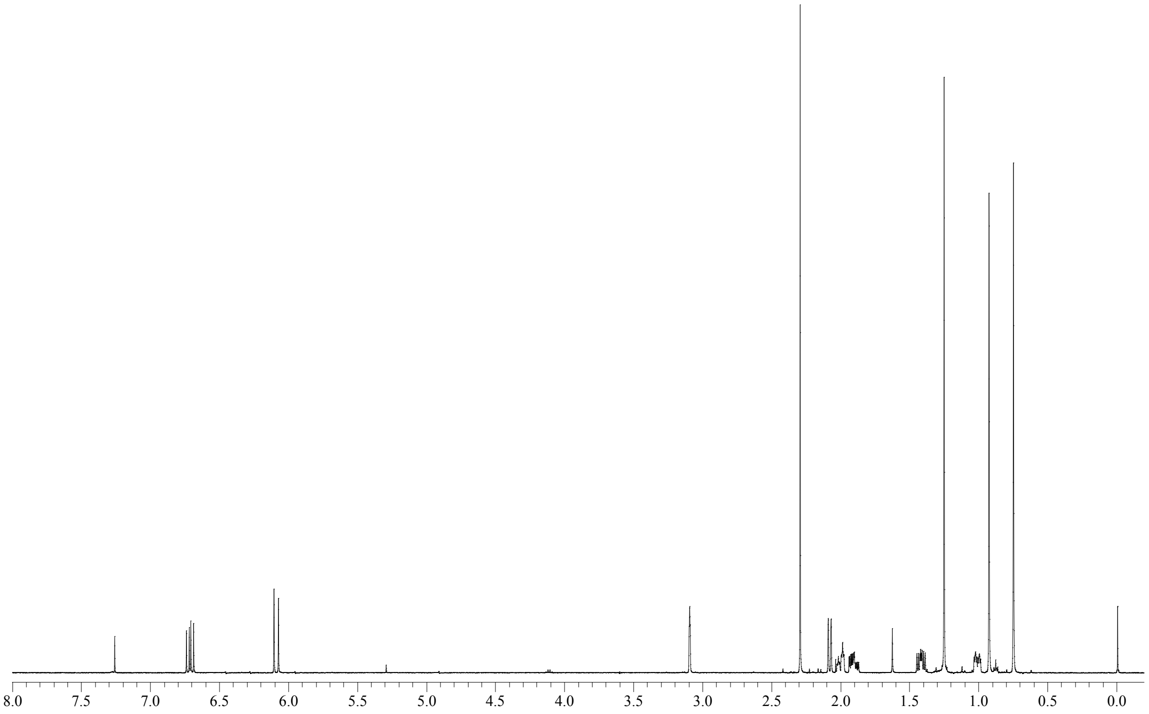

Figure S28. gCOSY spectrum of **14** in $CDCl_3$.

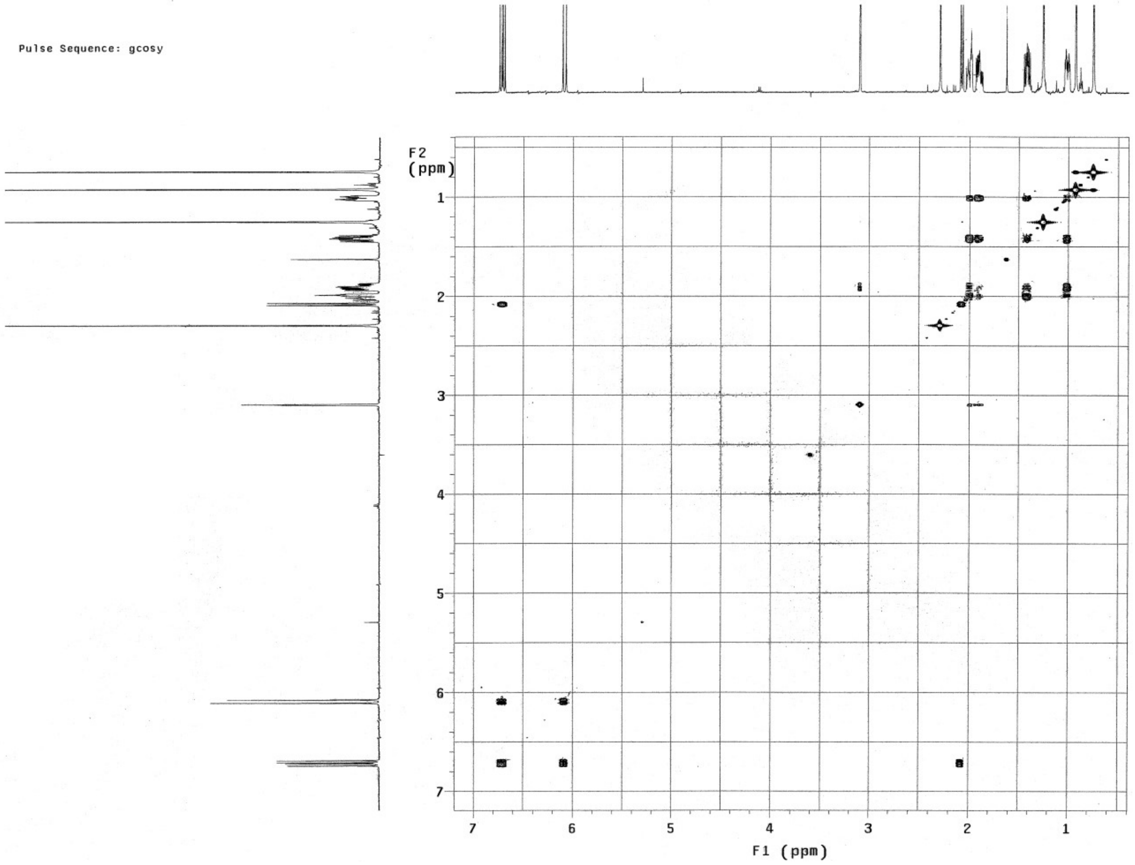

Figure S29. NOESY spectrum of **14** in $CDCl_3$.

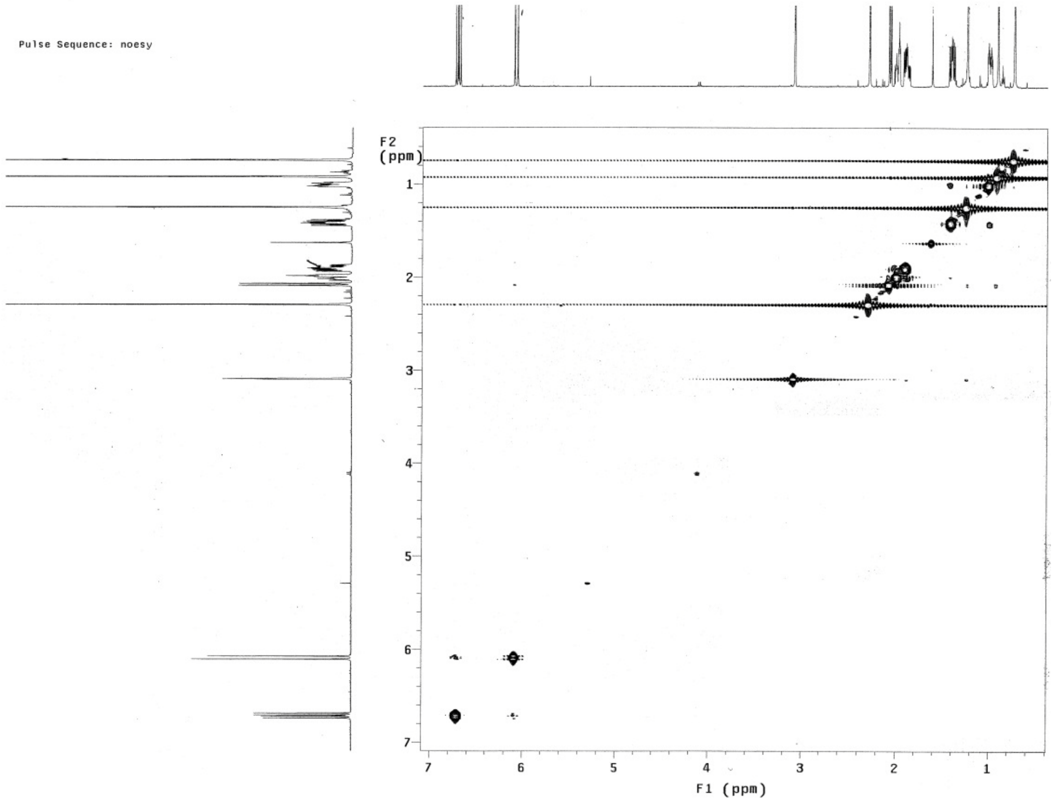

Figure S30. ^{1}H-NMR spectrum of **15** in $CDCl_3$.

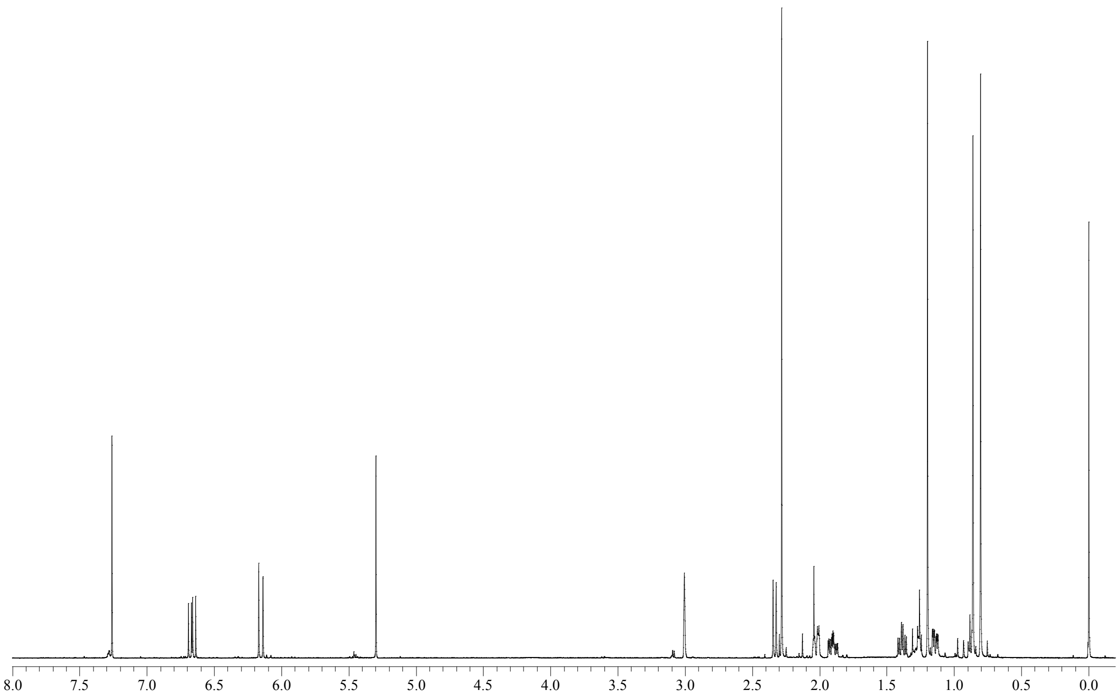

Figure S31. gCOSY spectrum of **15** in $CDCl_3$.

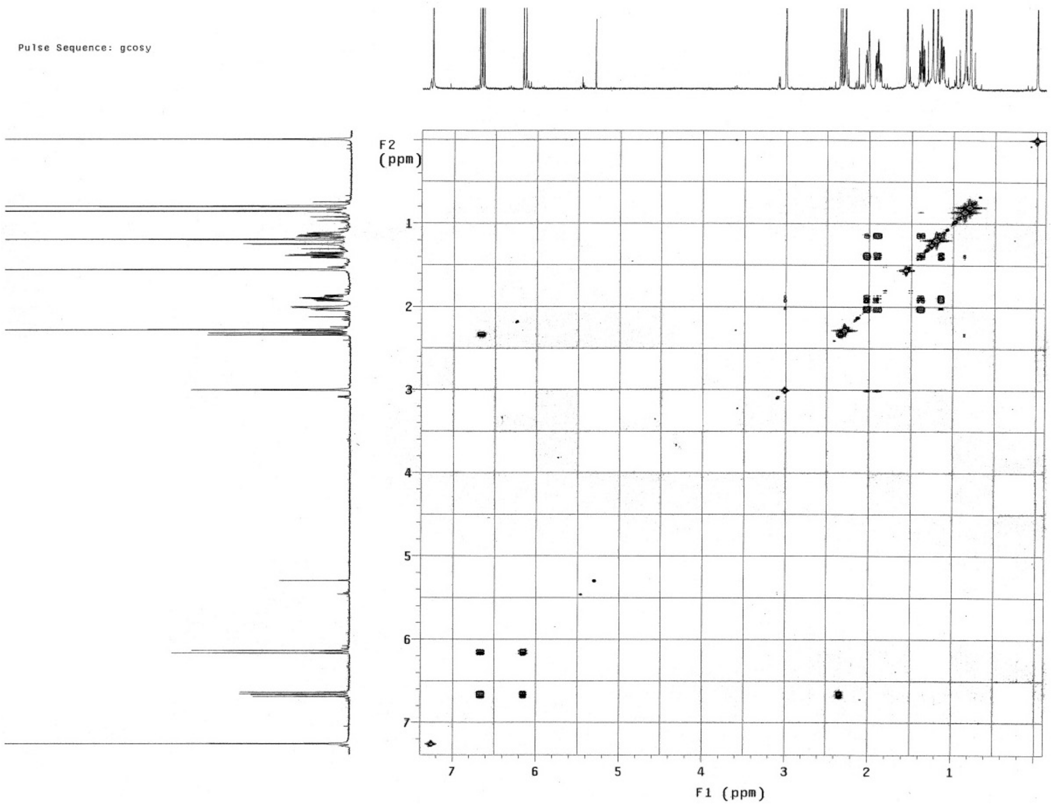

Figure S32. NOESY spectrum of **15** in $CDCl_3$.

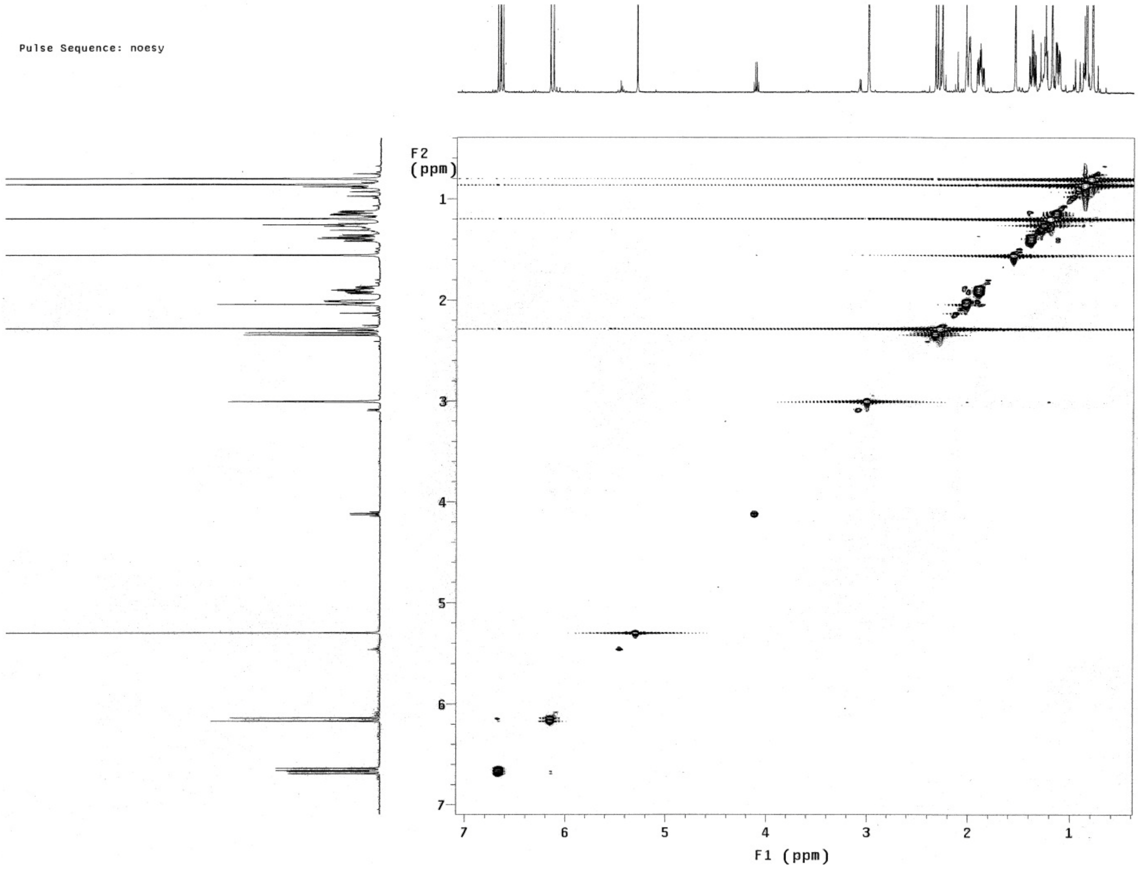

Figure S33. ^{1}H-NMR spectrum of **16** in $CDCl_3$.

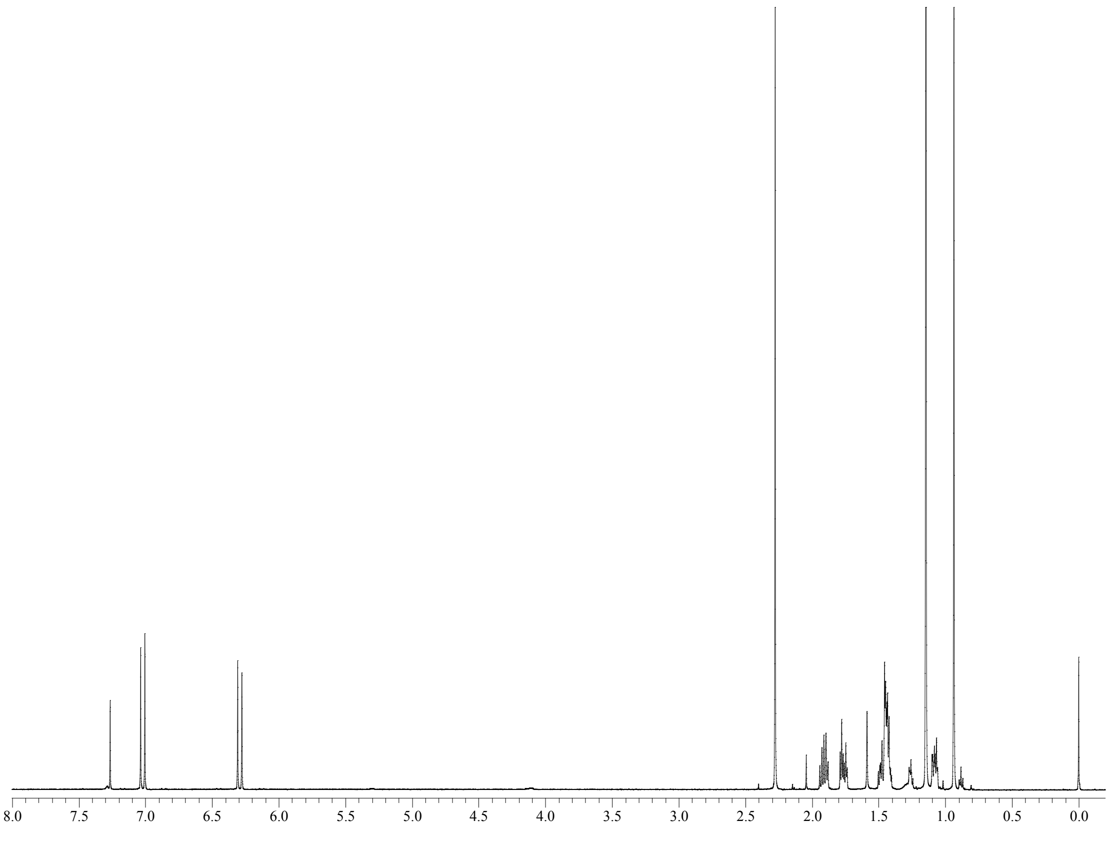

Figure S34. gCOSY spectrum of **16** in $CDCl_3$.

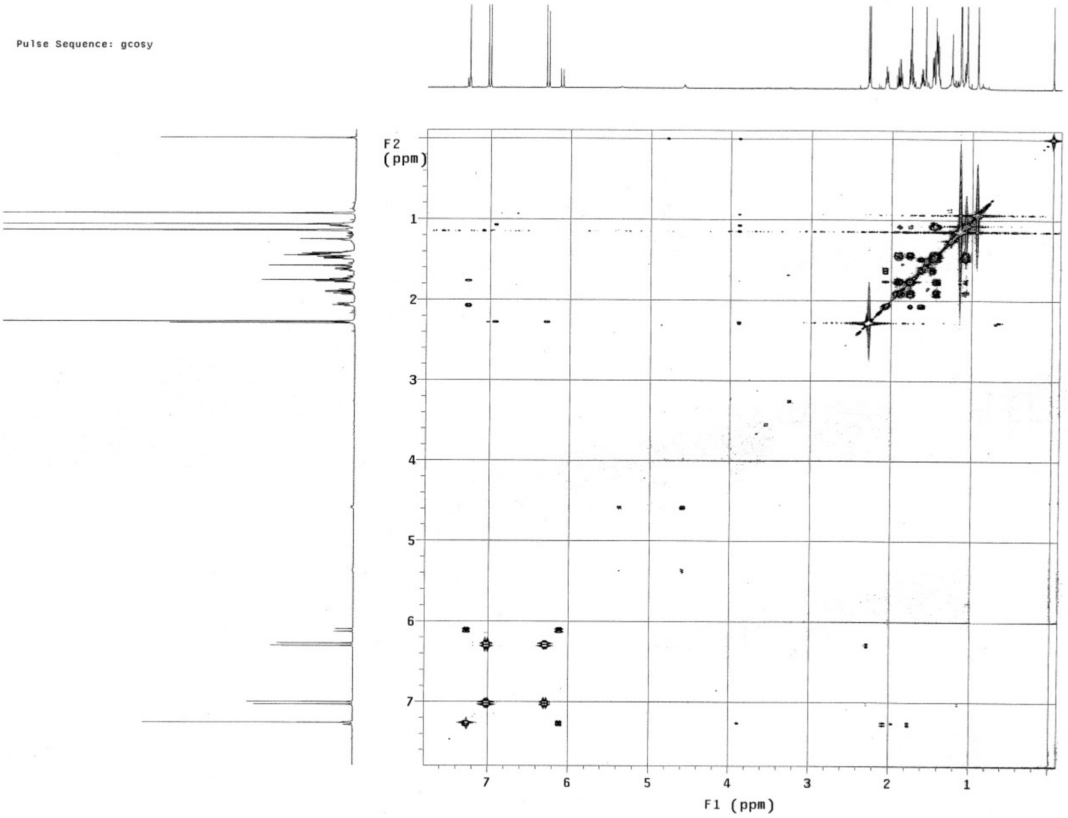

Figure S35. NOESY spectrum of **16** in $CDCl_3$.

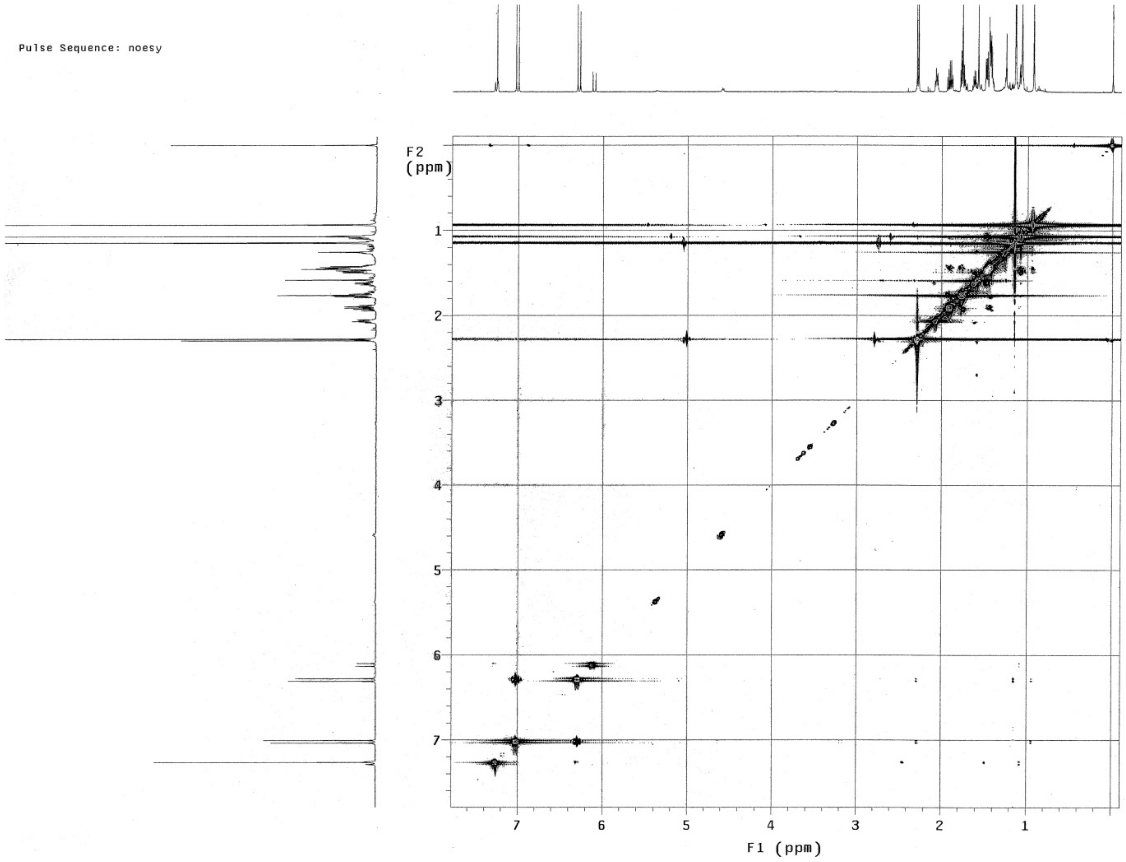

Figure S36. ^{1}H-NMR spectrum of **17** in $CDCl_3$.

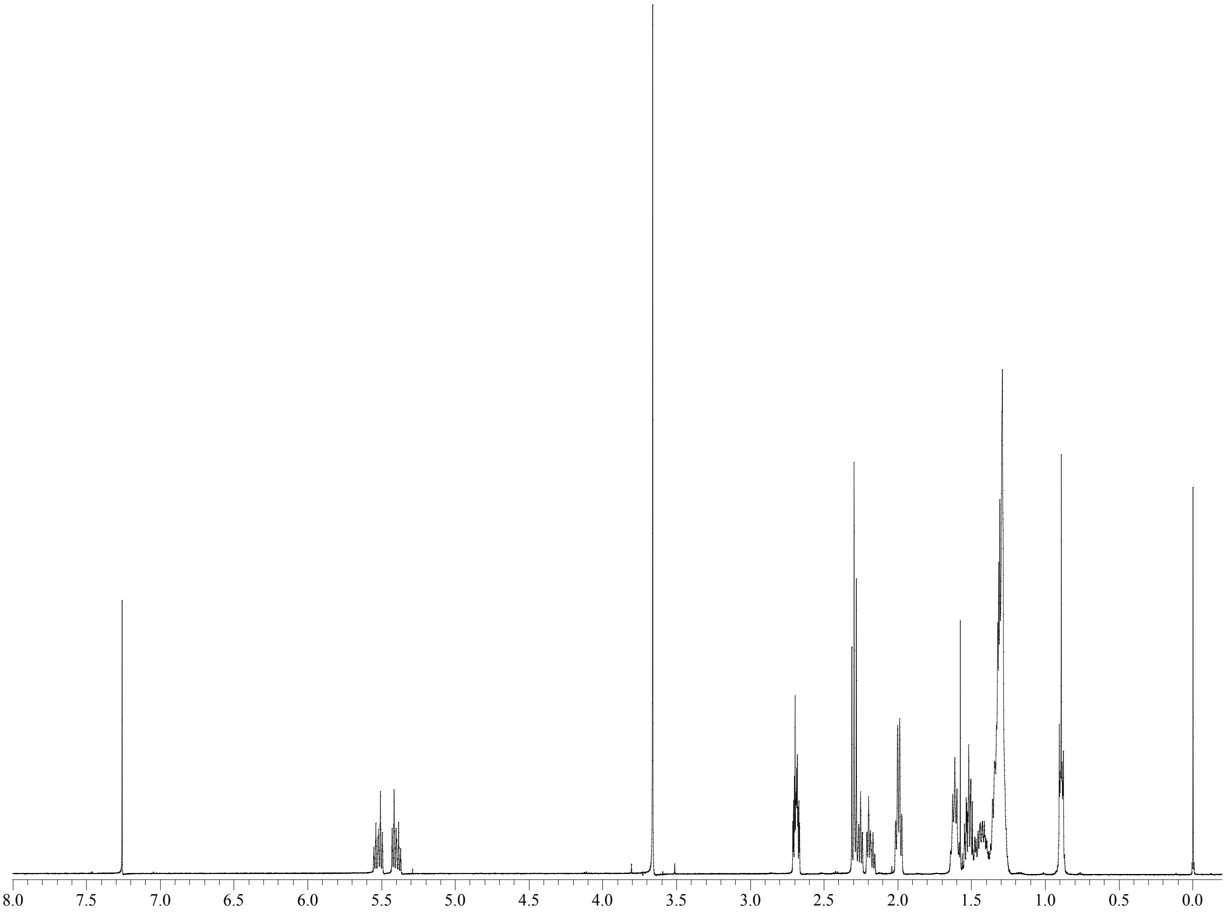

Figure S37. ^{13}C-NMR spectrum of **17** in $CDCl_3$.

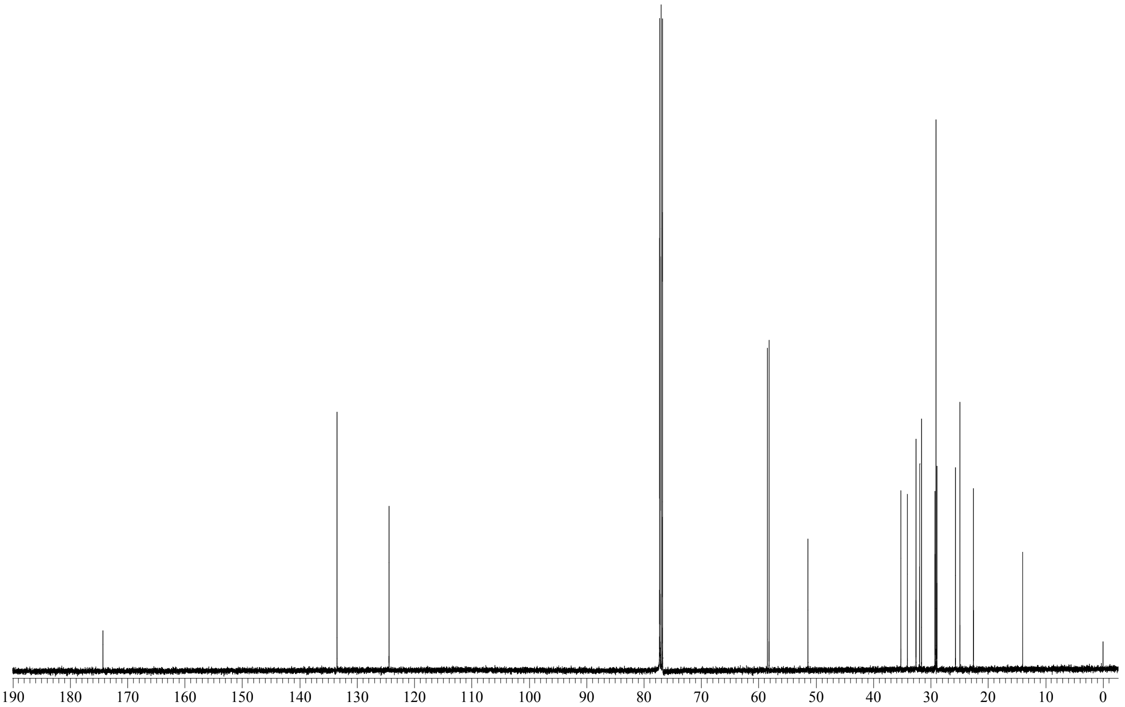

Figure S38. gCOSY spectrum of **17** in $CDCl_3$.

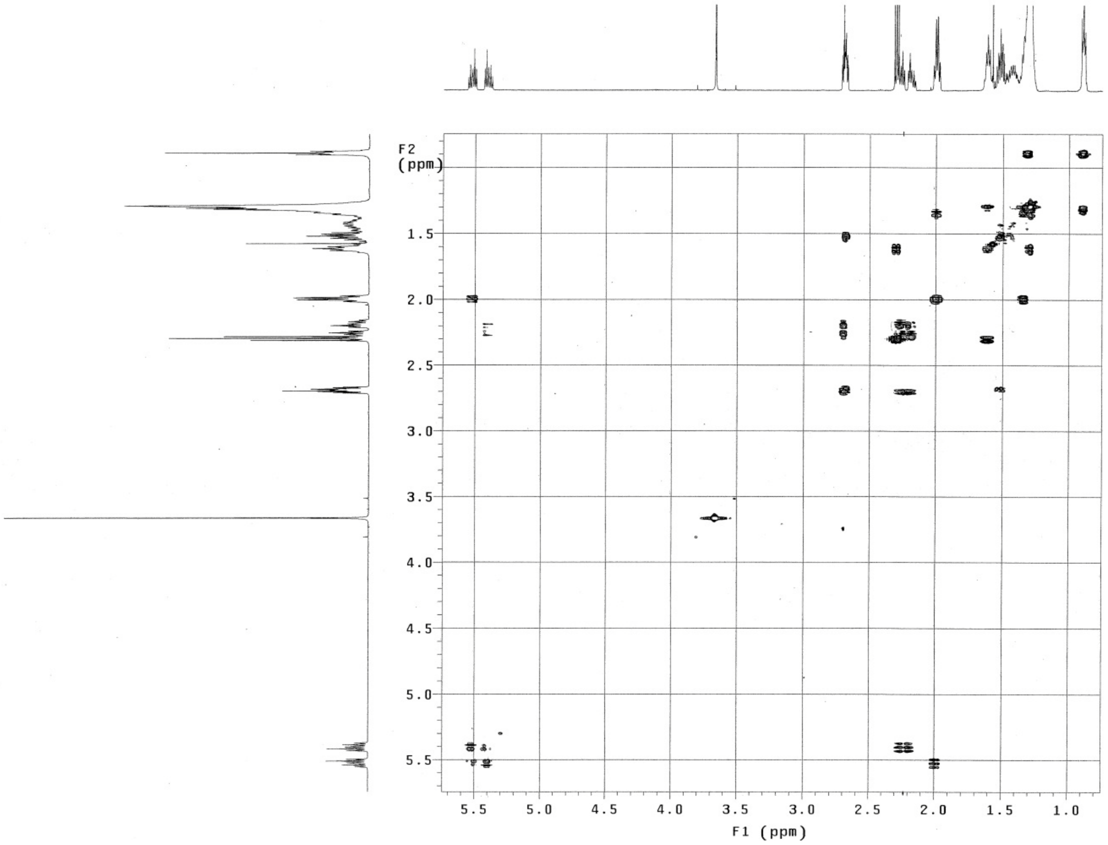

Figure S39. NOESY spectrum of **17** in $CDCl_3$.

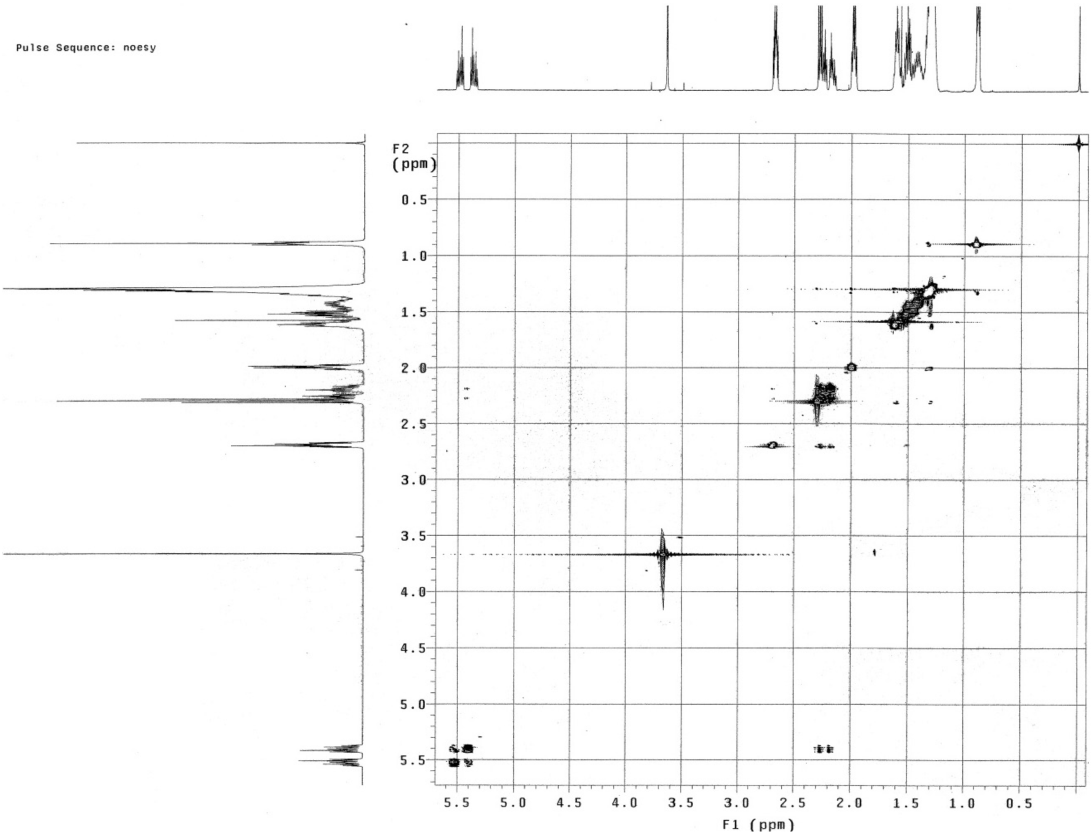

Figure S40. ^{1}H-NMR spectrum of **18** in $CDCl_3$.

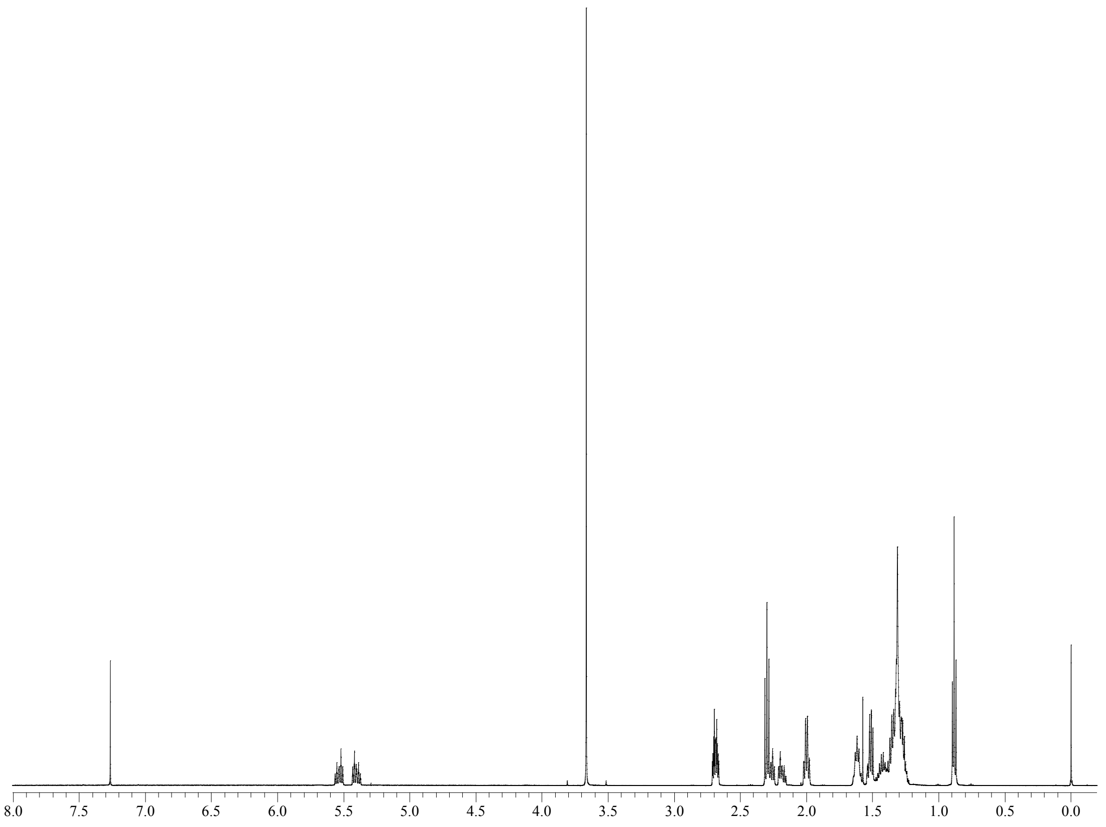

Figure S41. ^{13}C-NMR spectrum of **18** in $CDCl_3$.

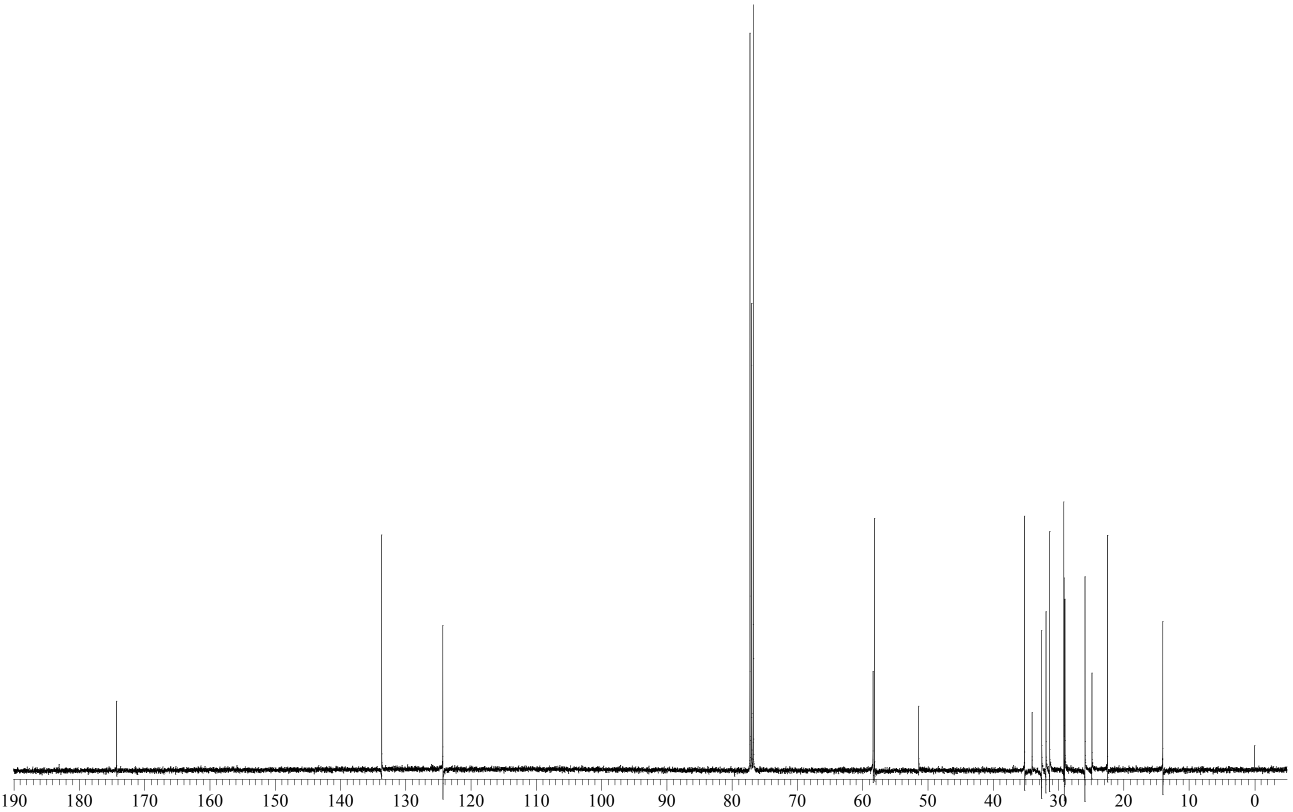

Figure S42. gCOSY spectrum of **18** in $CDCl_3$.

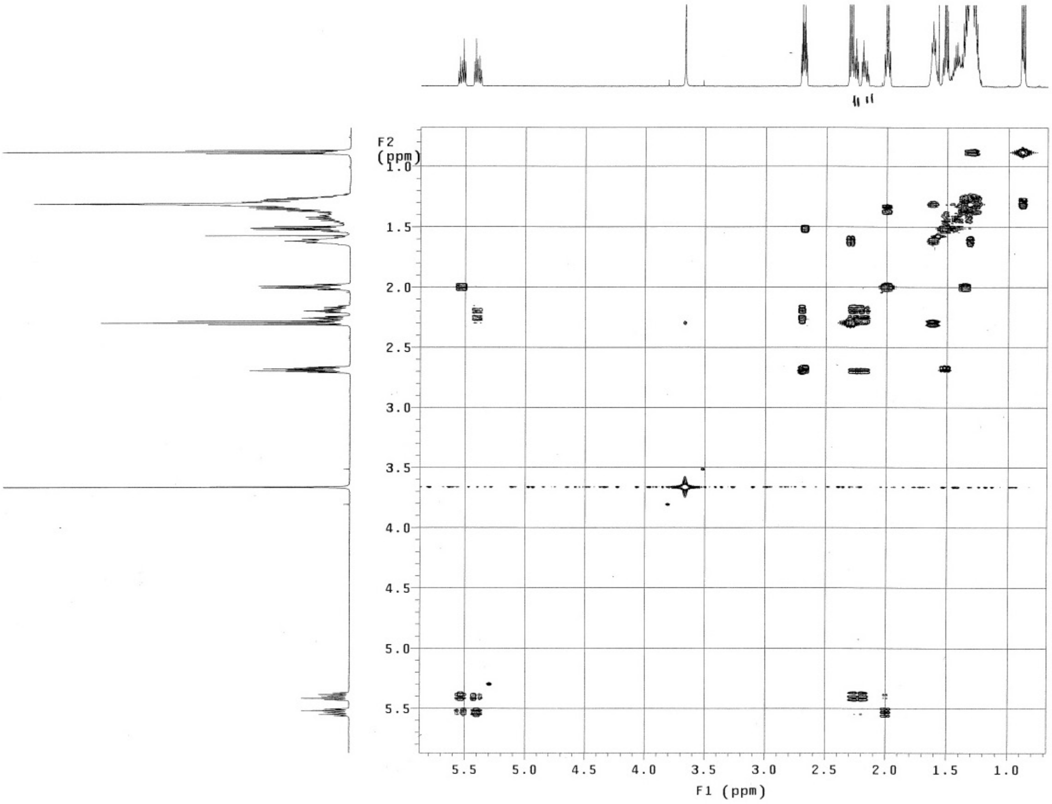

Figure S43. NOESY spectrum of **18** in $CDCl_3$.

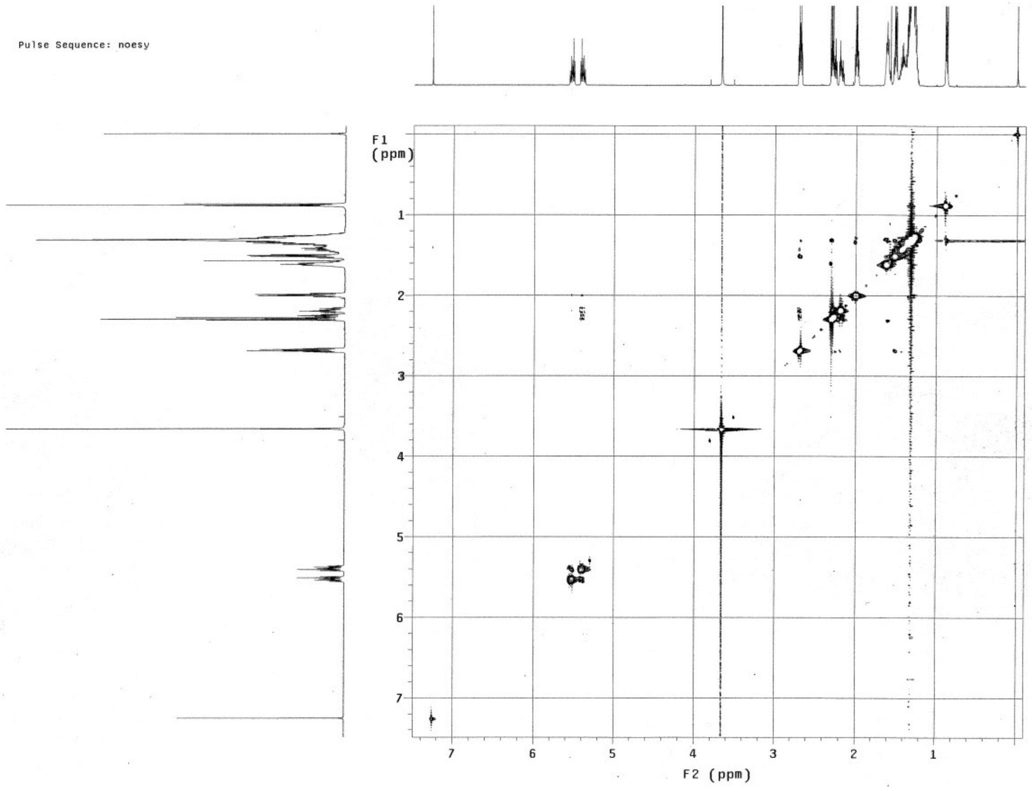

Figure S44. ^{1}H-NMR spectrum of **19** in $CDCl_3$.

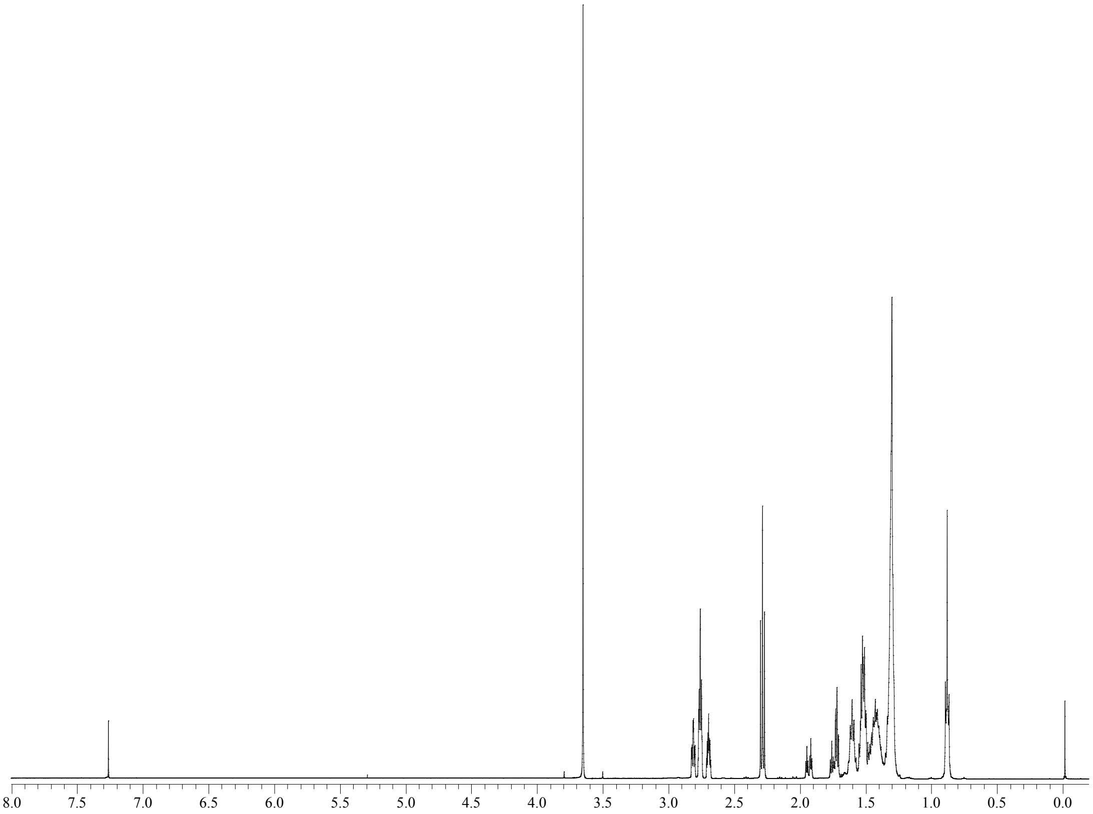

Figure S45. gCOSY spectrum of **19** in $CDCl_3$.

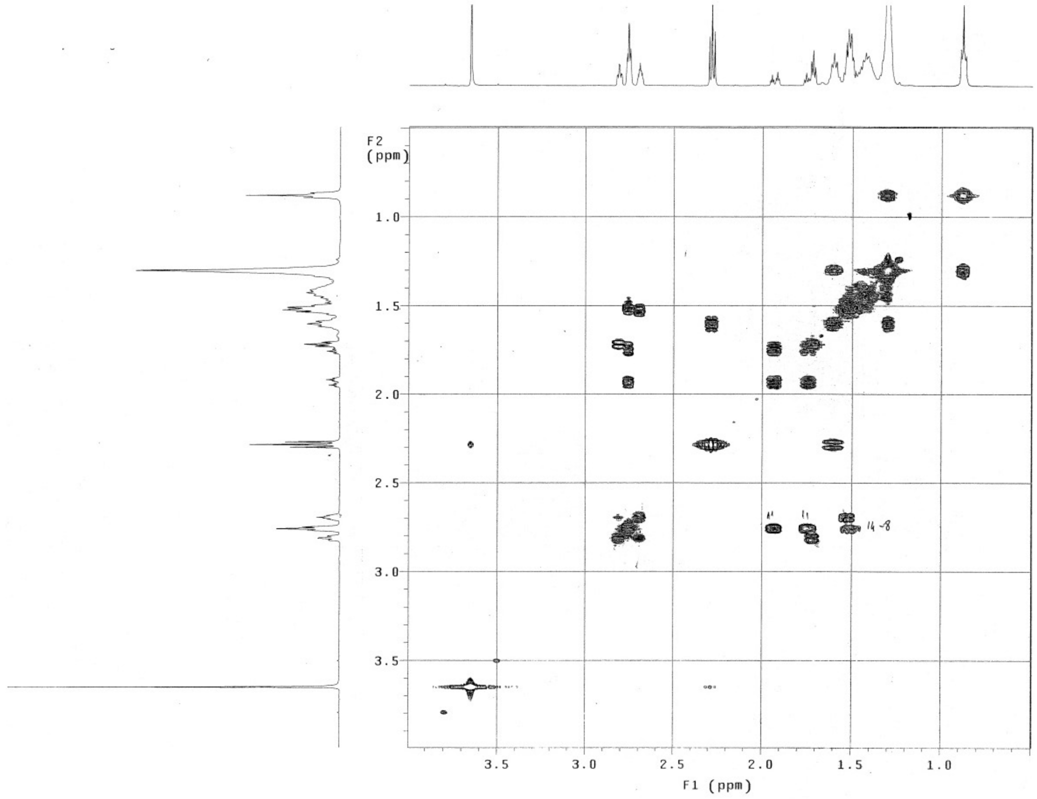

Figure S46. NOESY spectrum of **19** in $CDCl_3$.

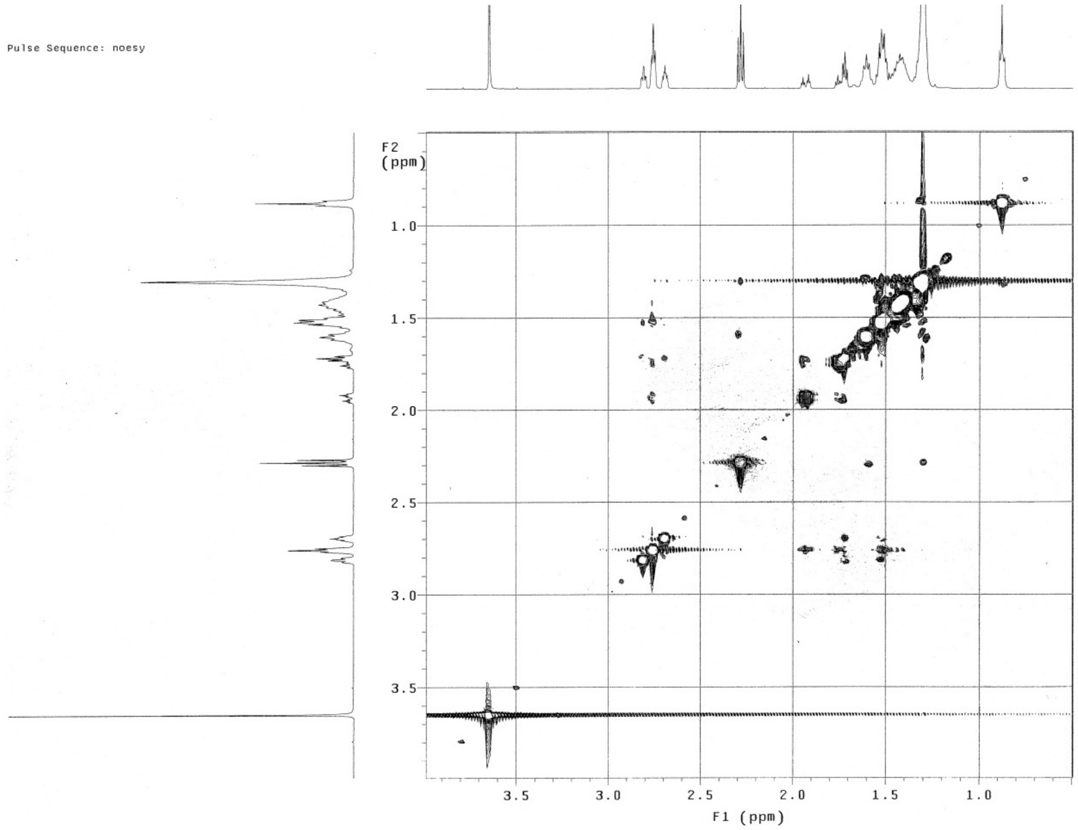

Figure S47. ^{1}H-NMR spectrum of **20** in $CDCl_3$.

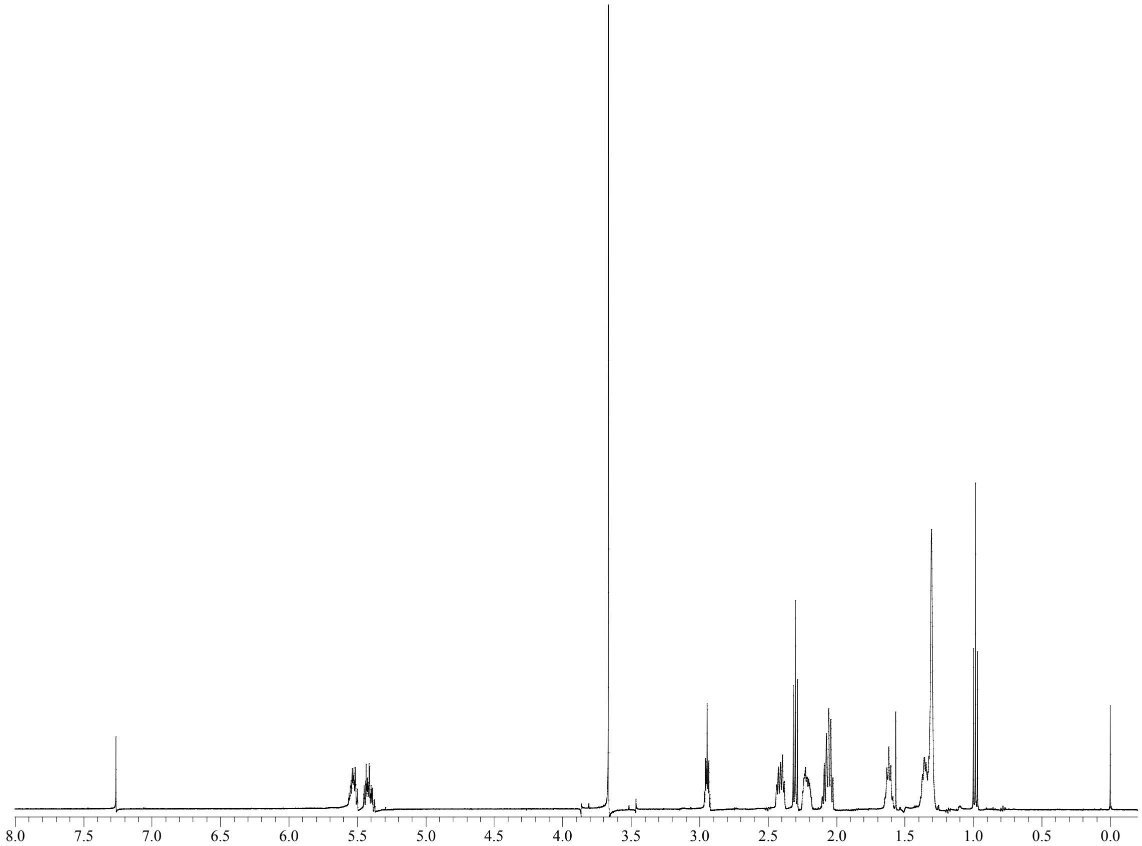

Figure S48. ^{13}C-NMR spectrum of **20** in $CDCl_3$.

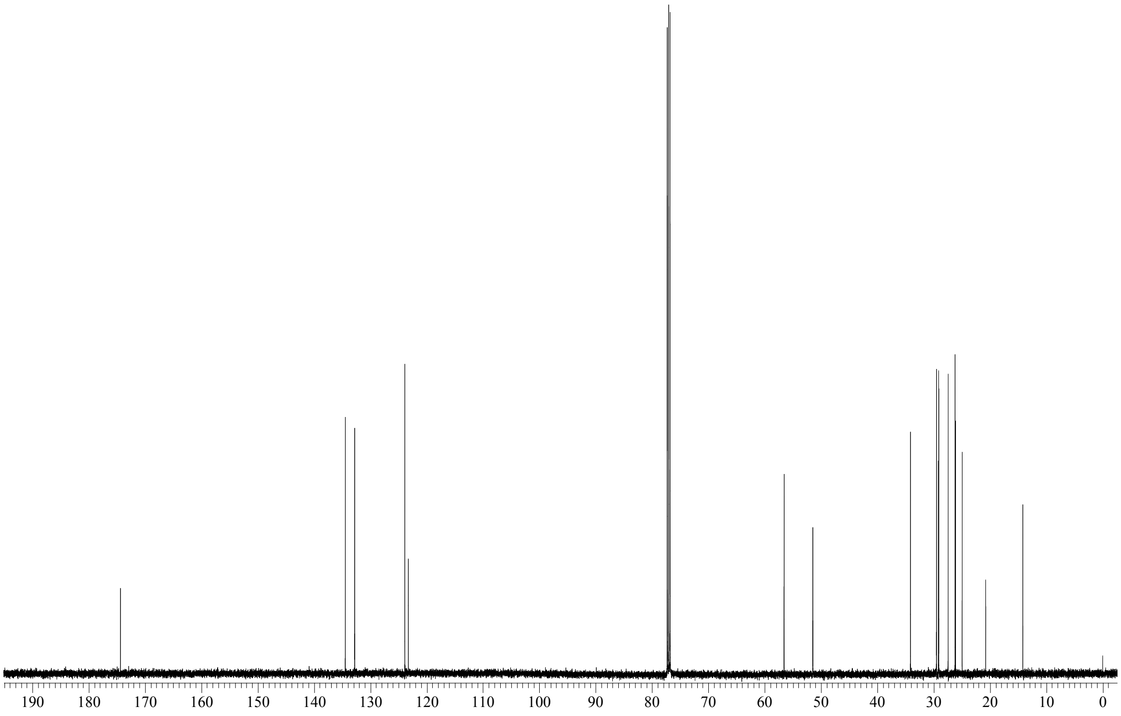

Figure S49. gCOSY spectrum of **20** in $CDCl_3$.

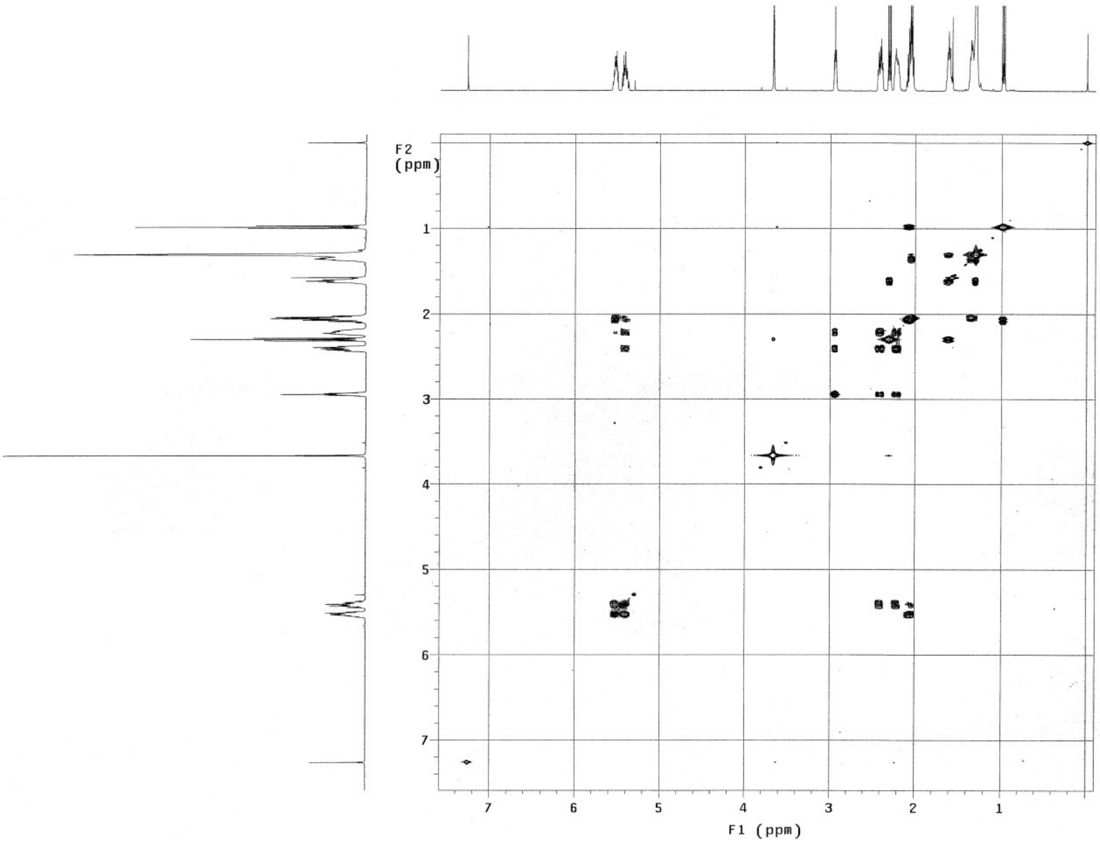

Figure S50. NOESY spectrum of **20** in $CDCl_3$.

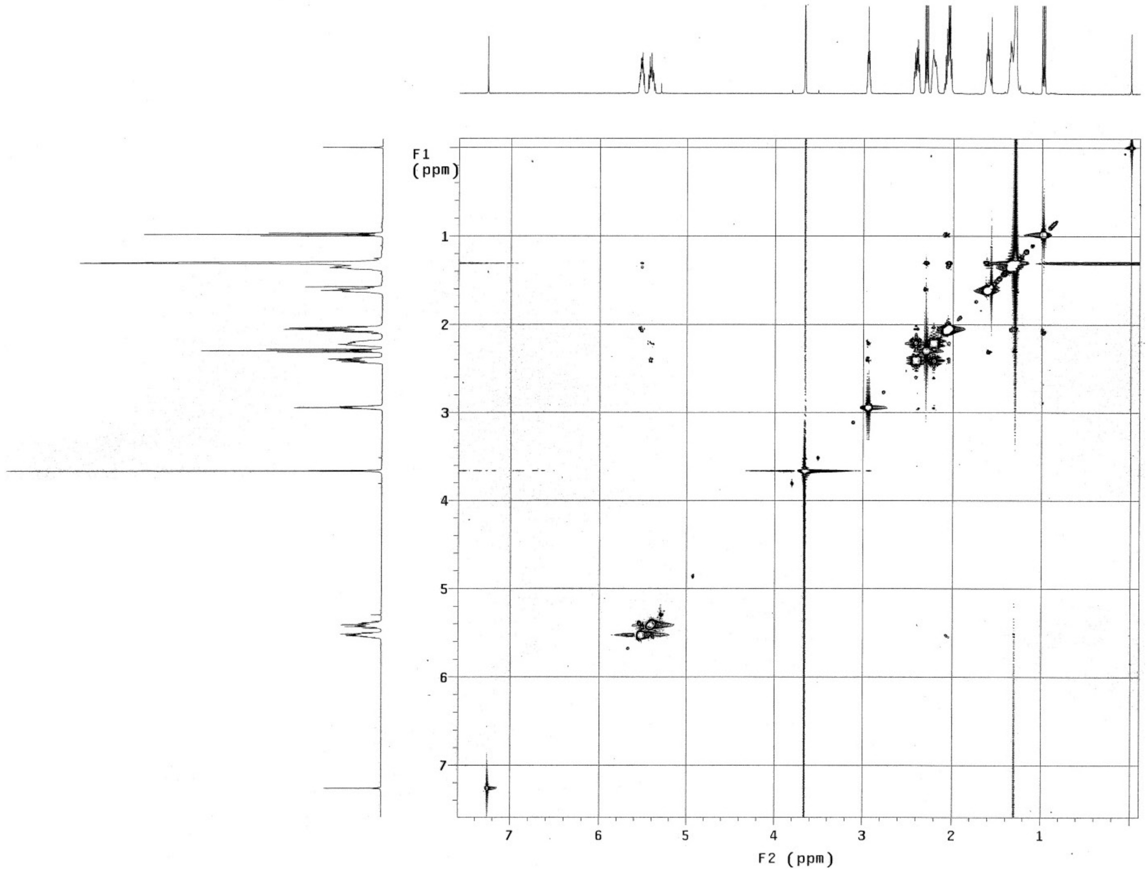

Figure S51. ^{1}H-NMR spectrum of **21** in $CDCl_3$.

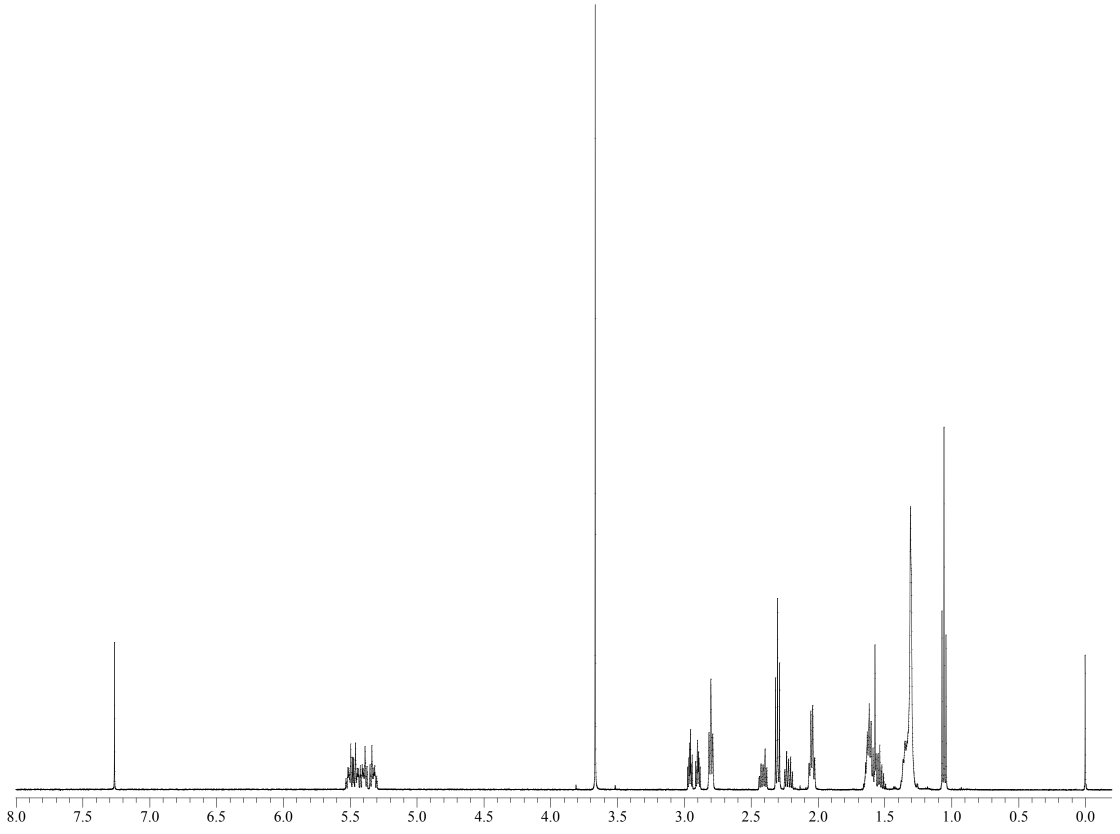

Figure S52. ^{13}C-NMR spectrum of **21** in $CDCl_3$.

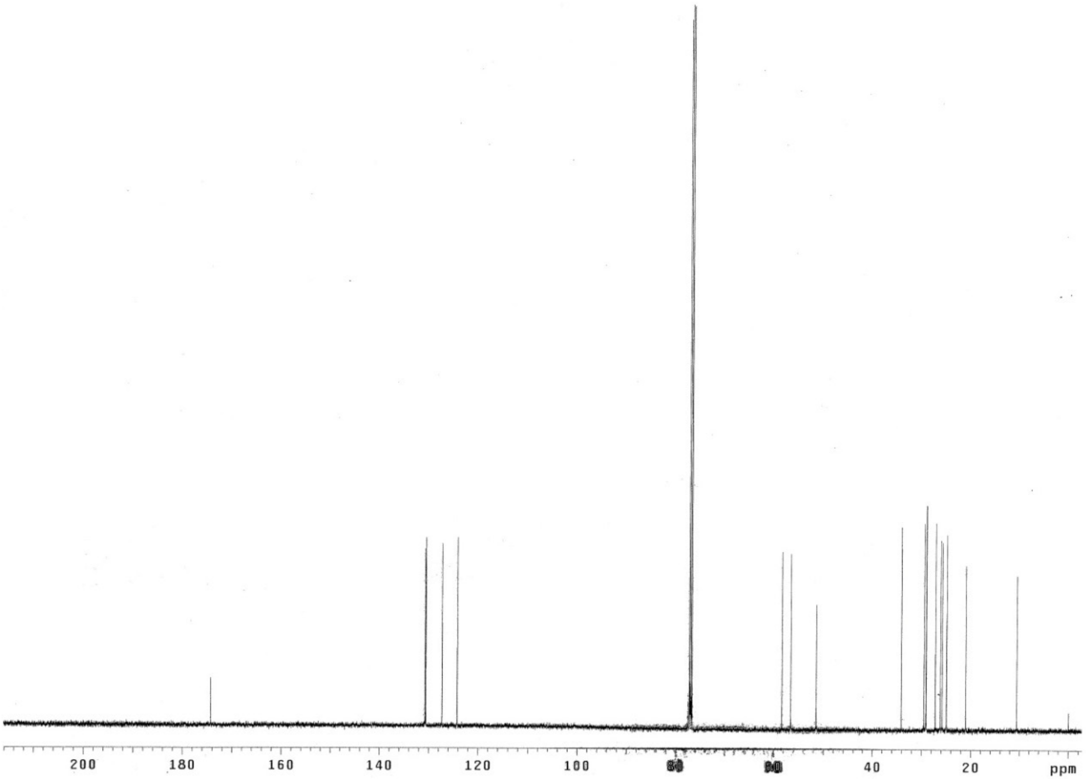

Figure S53. gCOSY spectrum of **21** in $CDCl_3$.

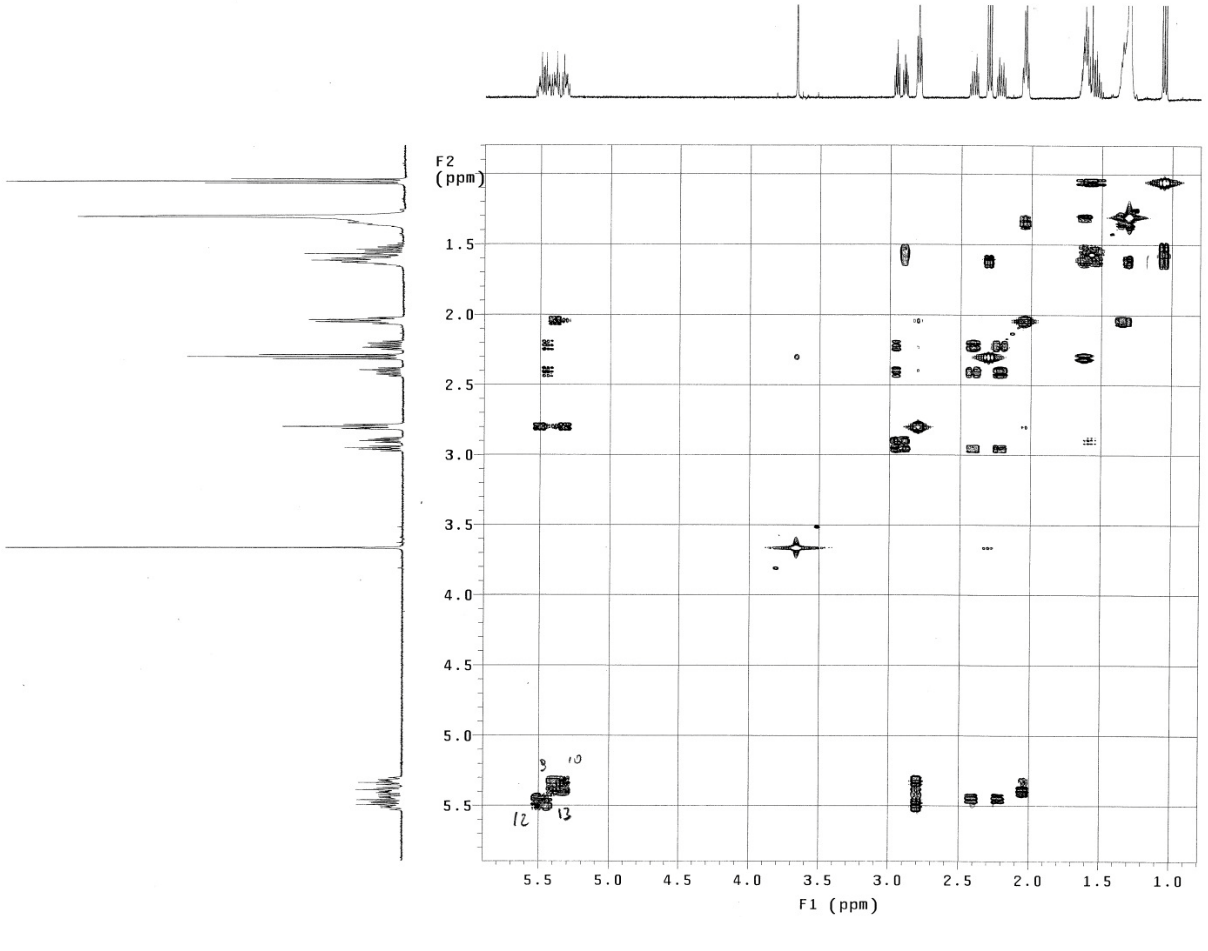

Figure S54. NOESY spectrum of **21** in $CDCl_3$.

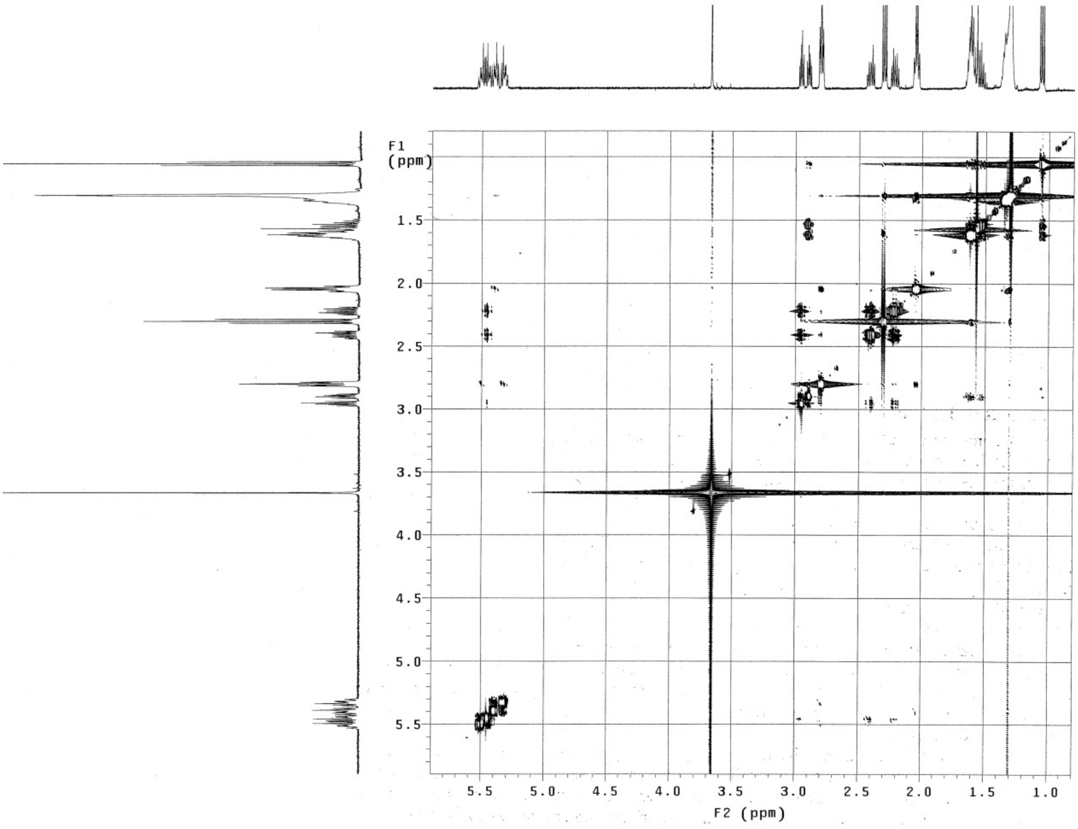

Figure S55. ^{1}H-NMR spectrum of **22** in $CDCl_3$.

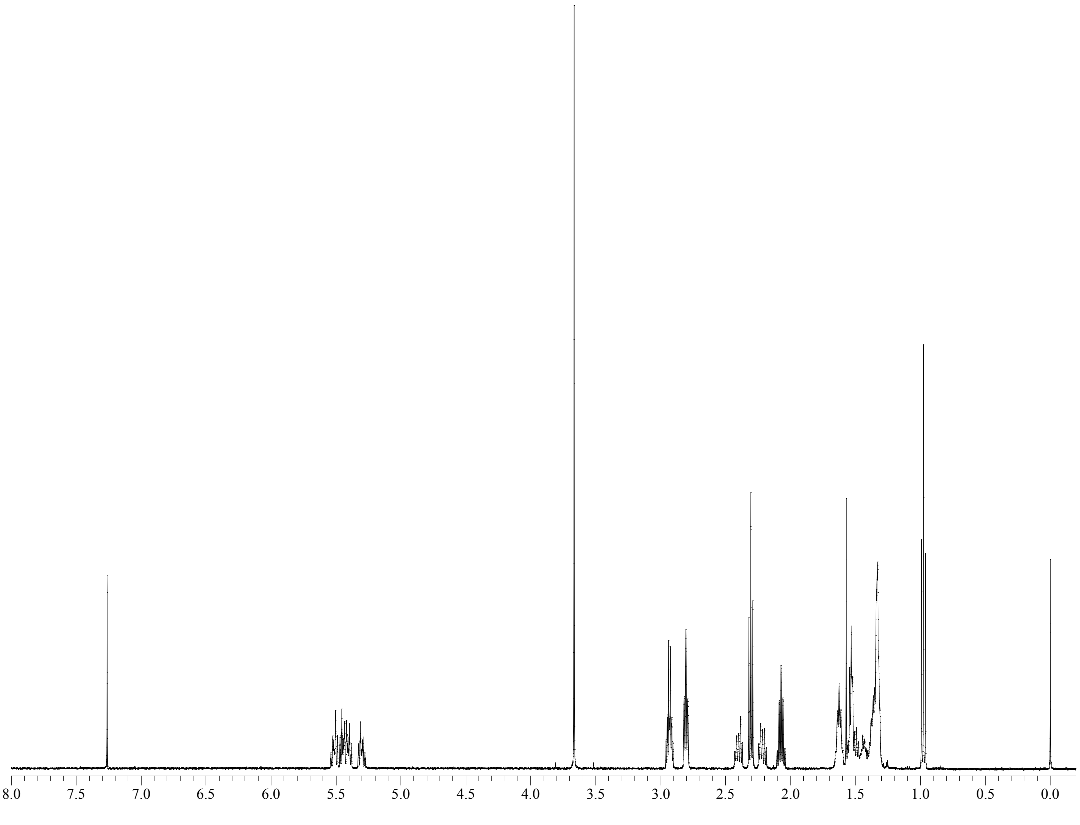

Figure S56. ^{13}C-NMR spectrum of 22 in $CDCl_3$.

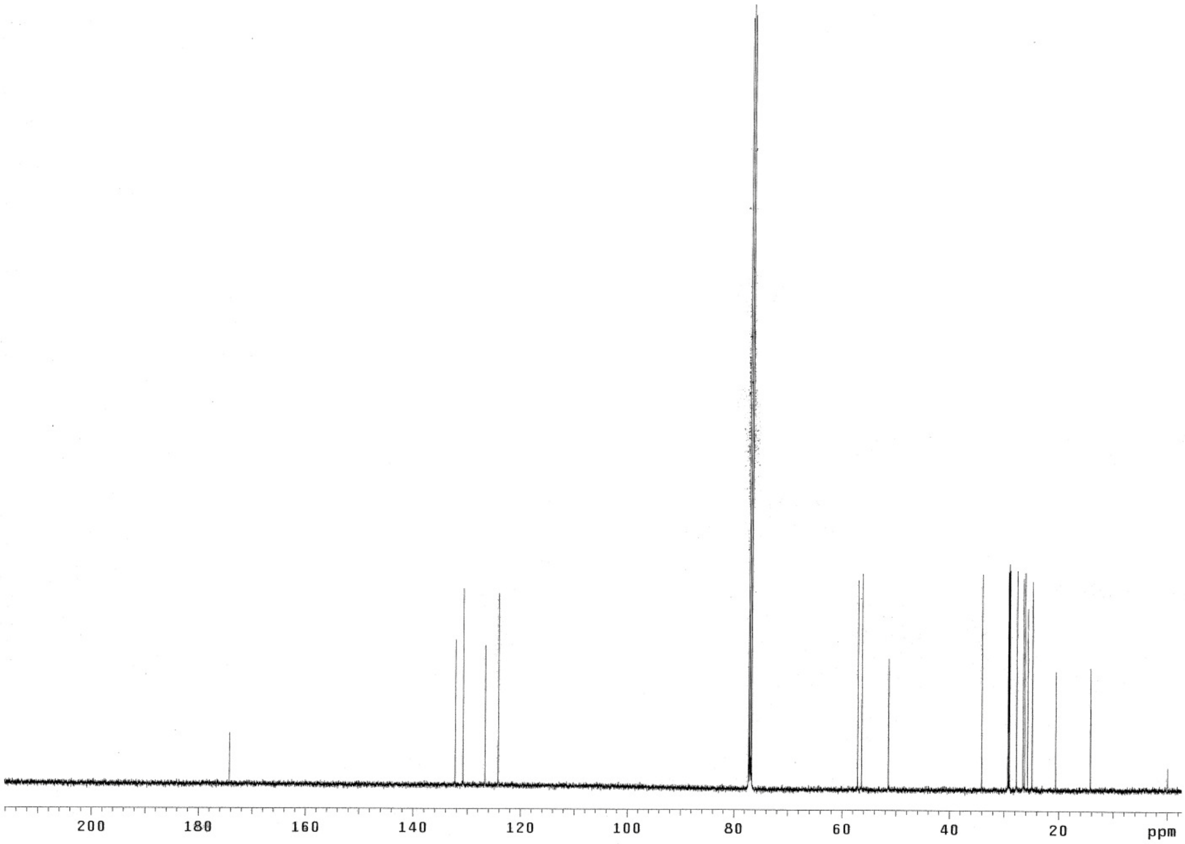

Figure S57. gCOSY spectrum of **22** in $CDCl_3$.

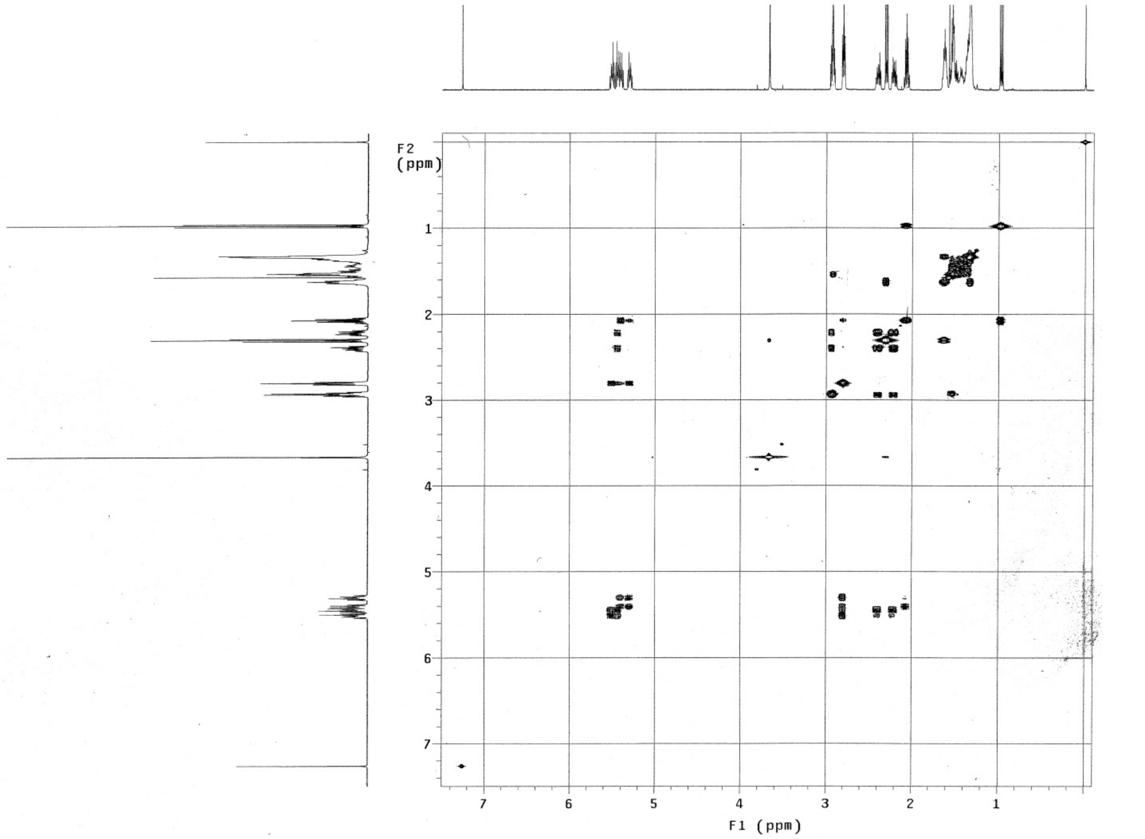

Figure S58. NOESY spectrum of **22** in $CDCl_3$.

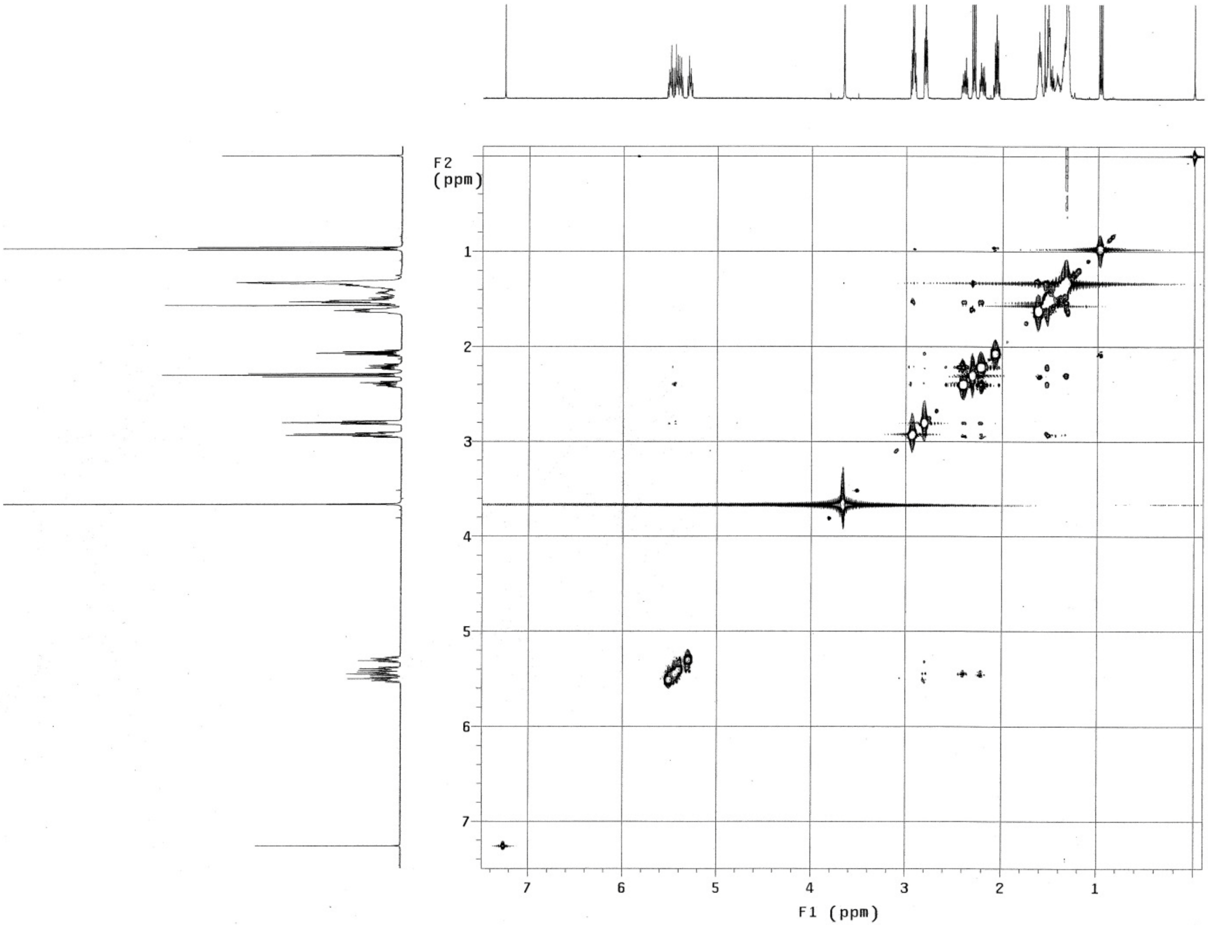

Figure S59. ^{1}H-NMR spectrum of **23** in $CDCl_3$.

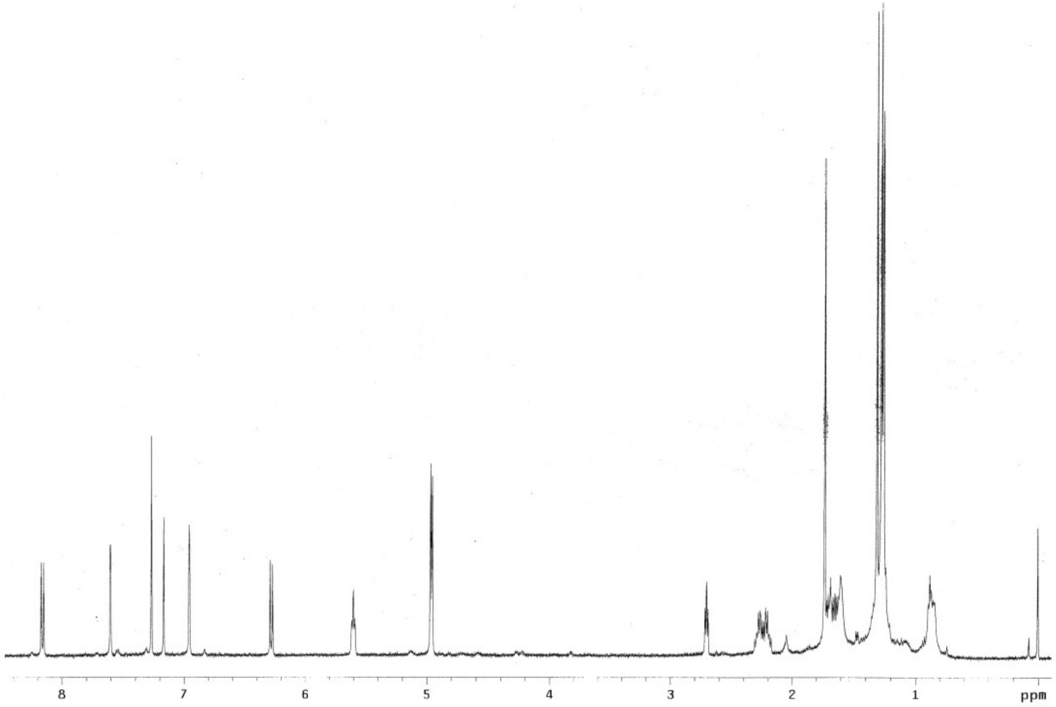

Figure S60. ^{13}C-NMR spectrum of **23** in $CDCl_3$.

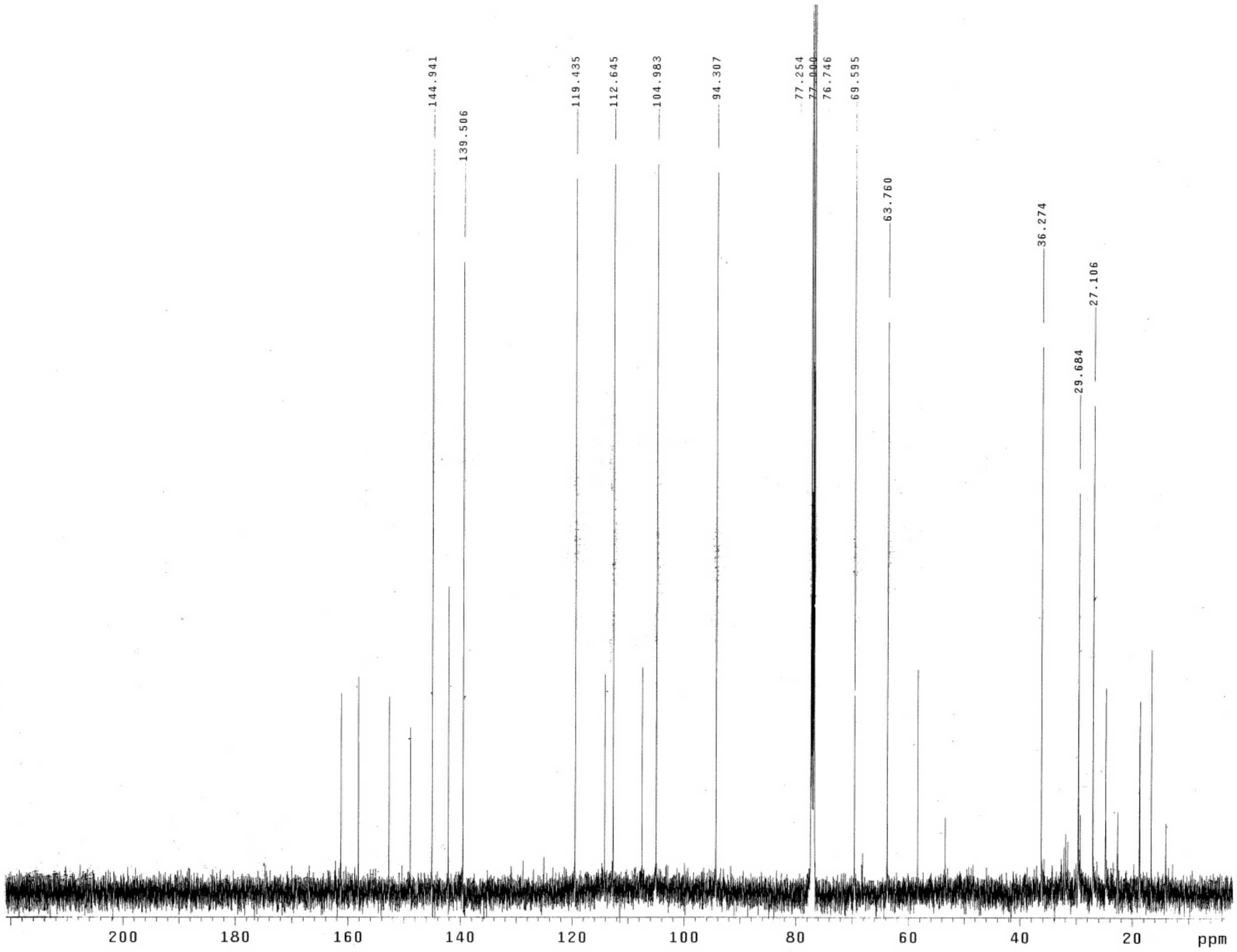

Figure S61. ^{1}H-NMR spectrum of **17** (R), **18** DHB in $CDCl_3$.

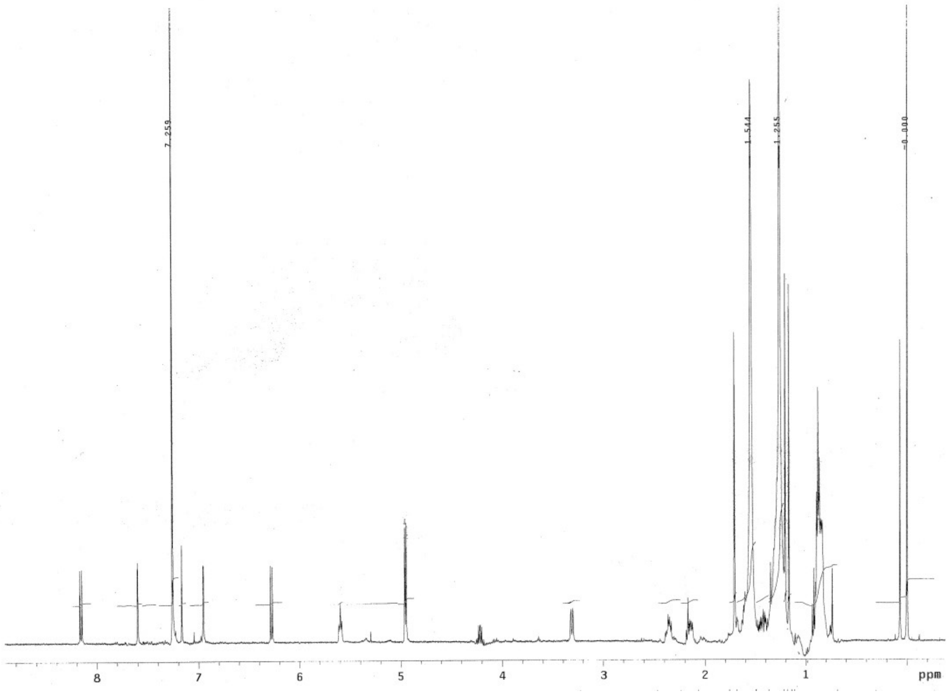

Mass Spectra

Figure S62. Mass spectrum of **9**.

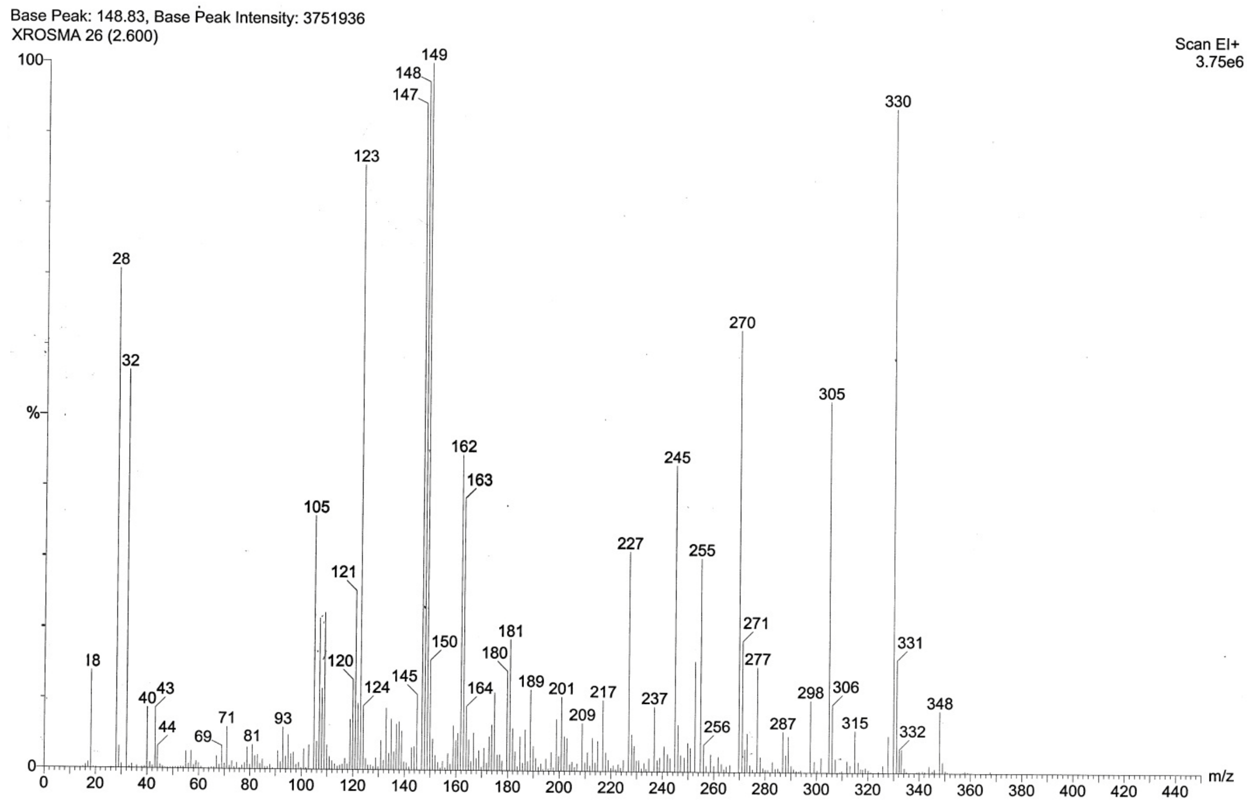

Figure S63. Mass spectrum of **10**.

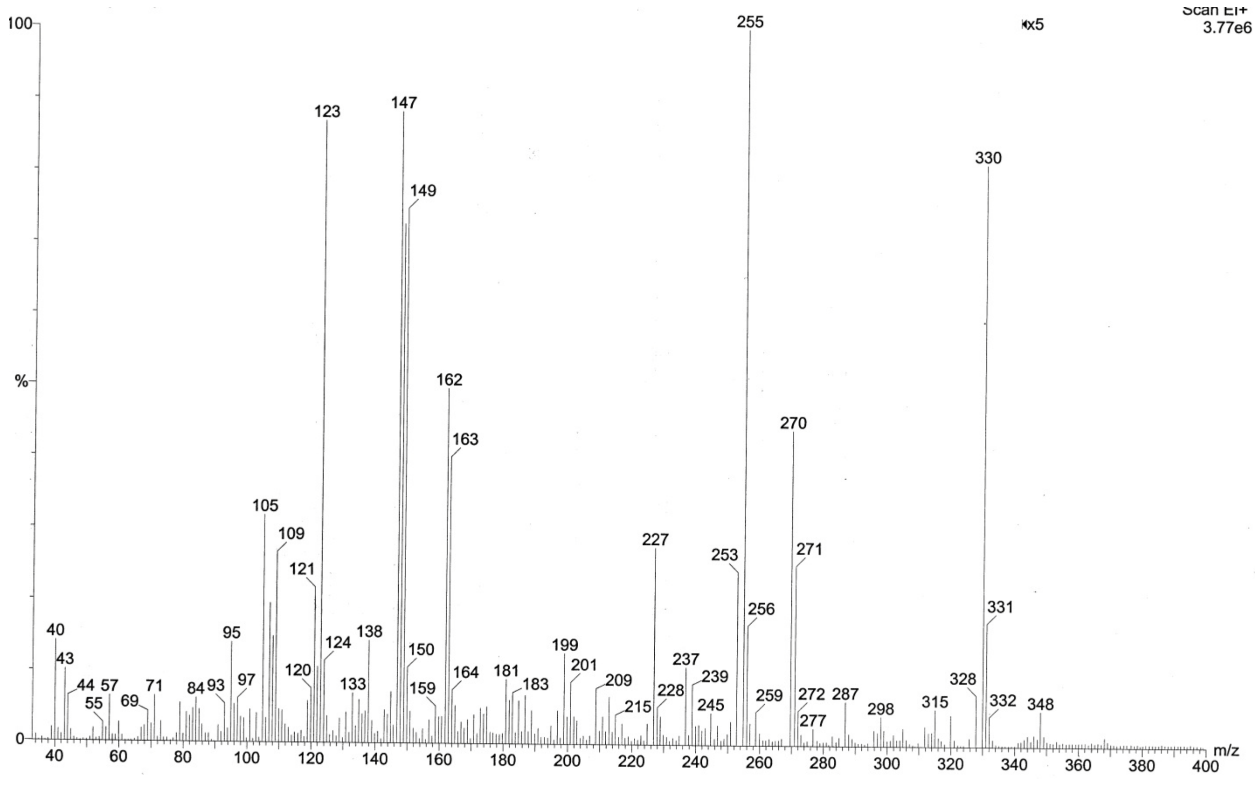

Figure S64. Mass spectrum of **17**.

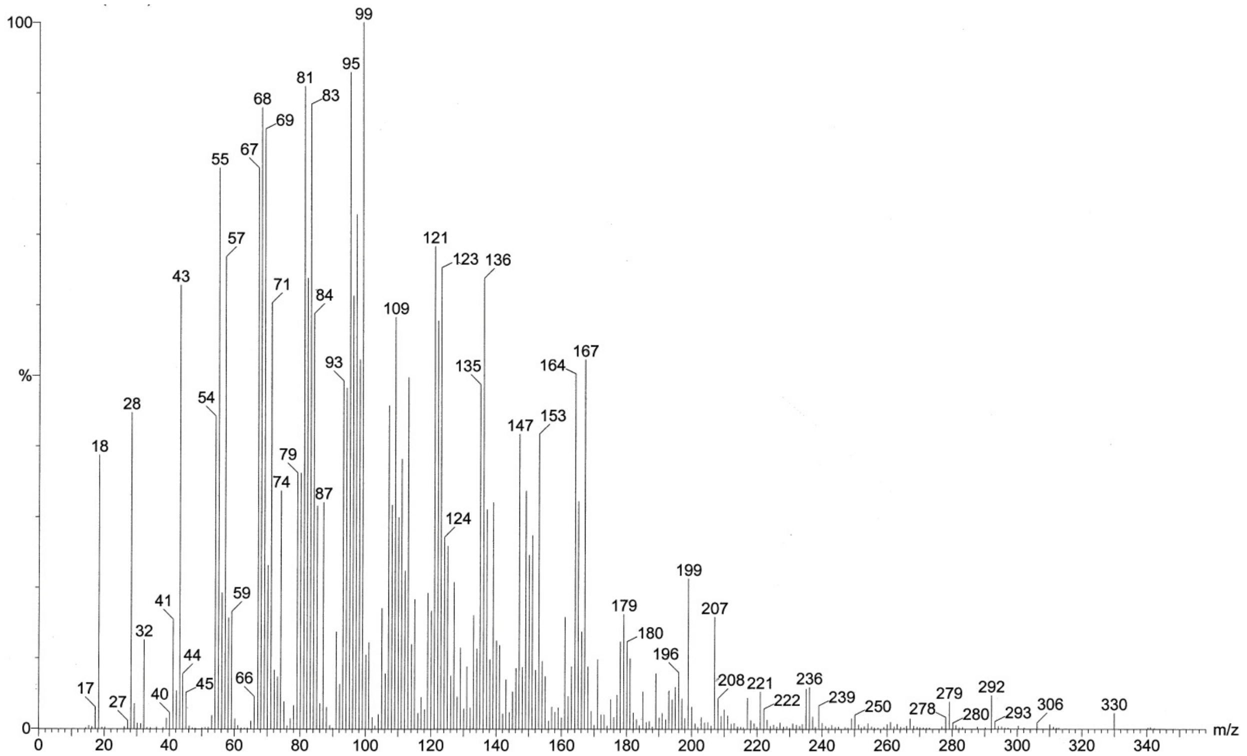

Figure S65. Mass spectrum of **18**.

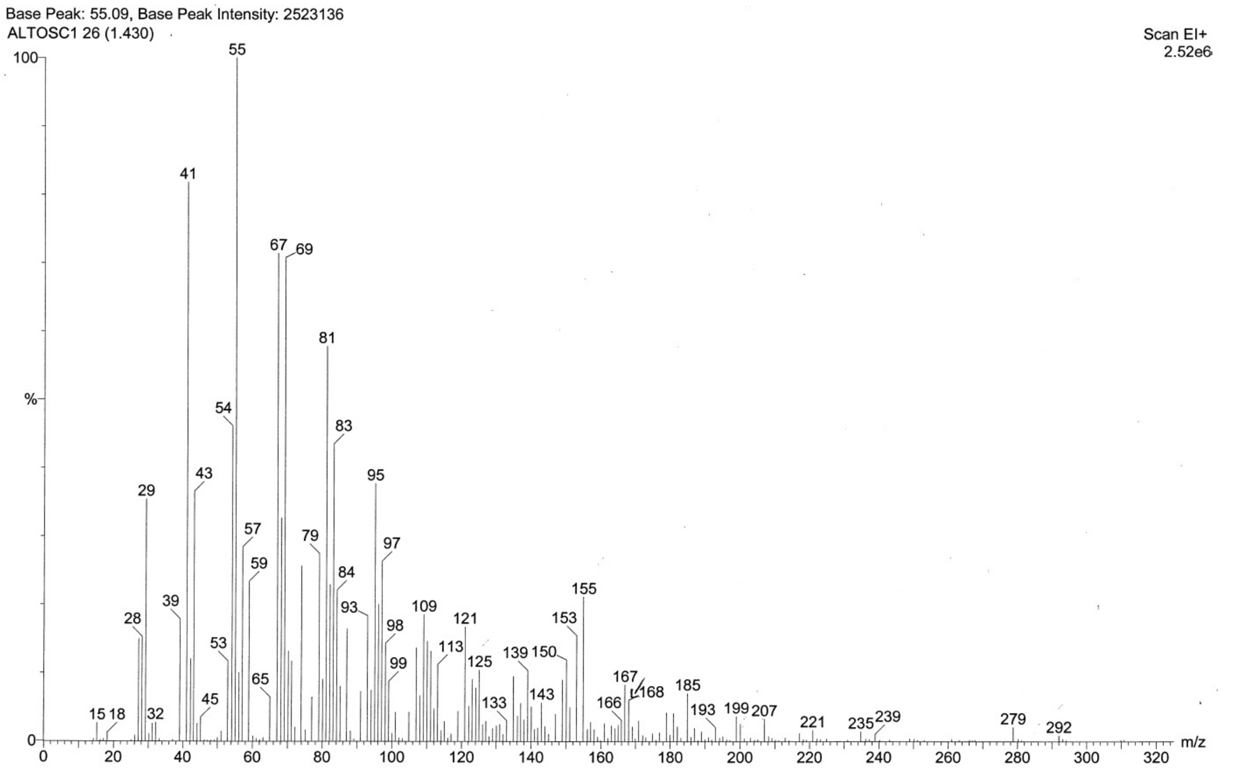

Figure S66. Mass spectrum of **19**.

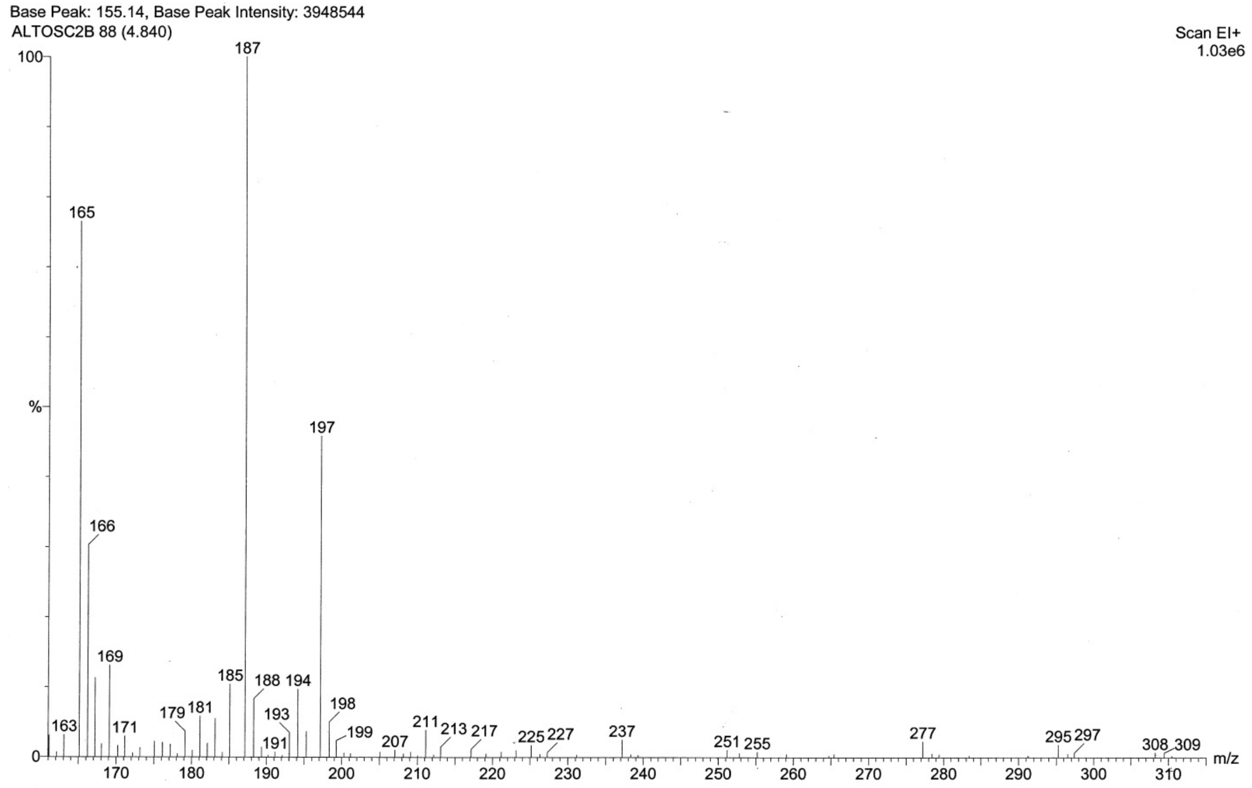

Figure S67. Mass spectrum of **20**.

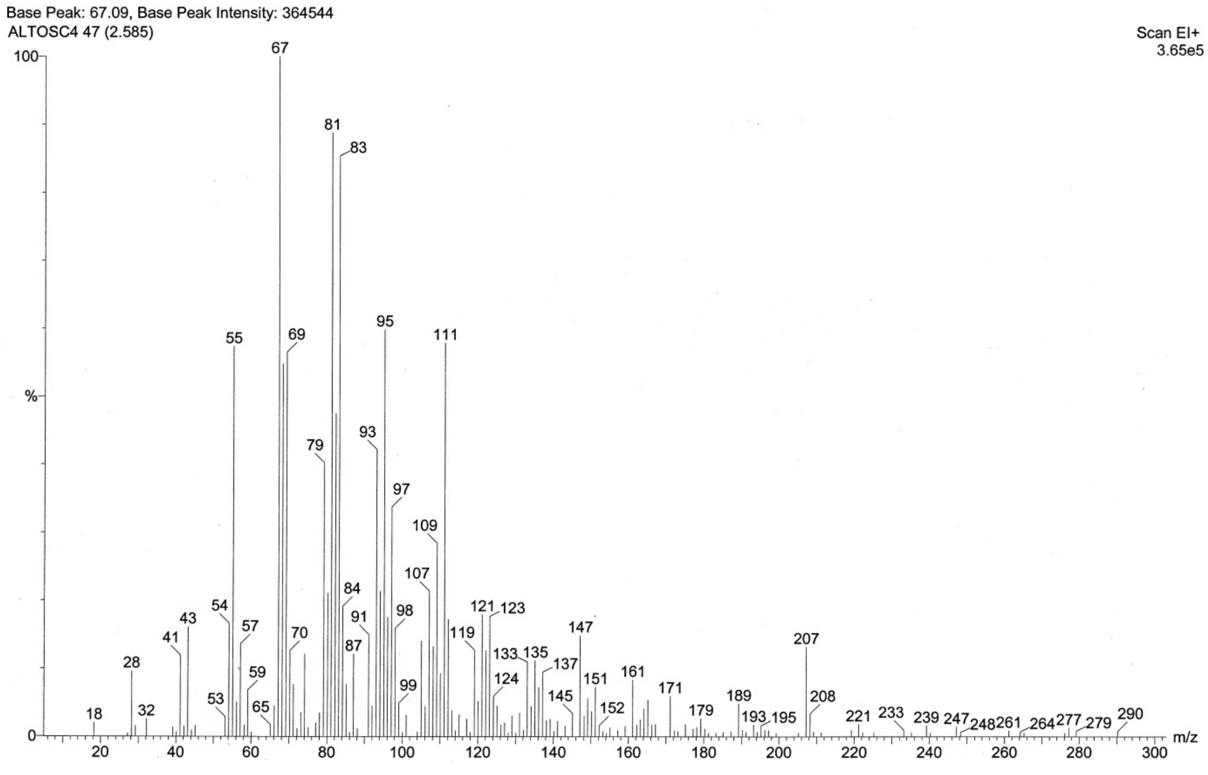

Figure S68. Mass spectrum of **21**.

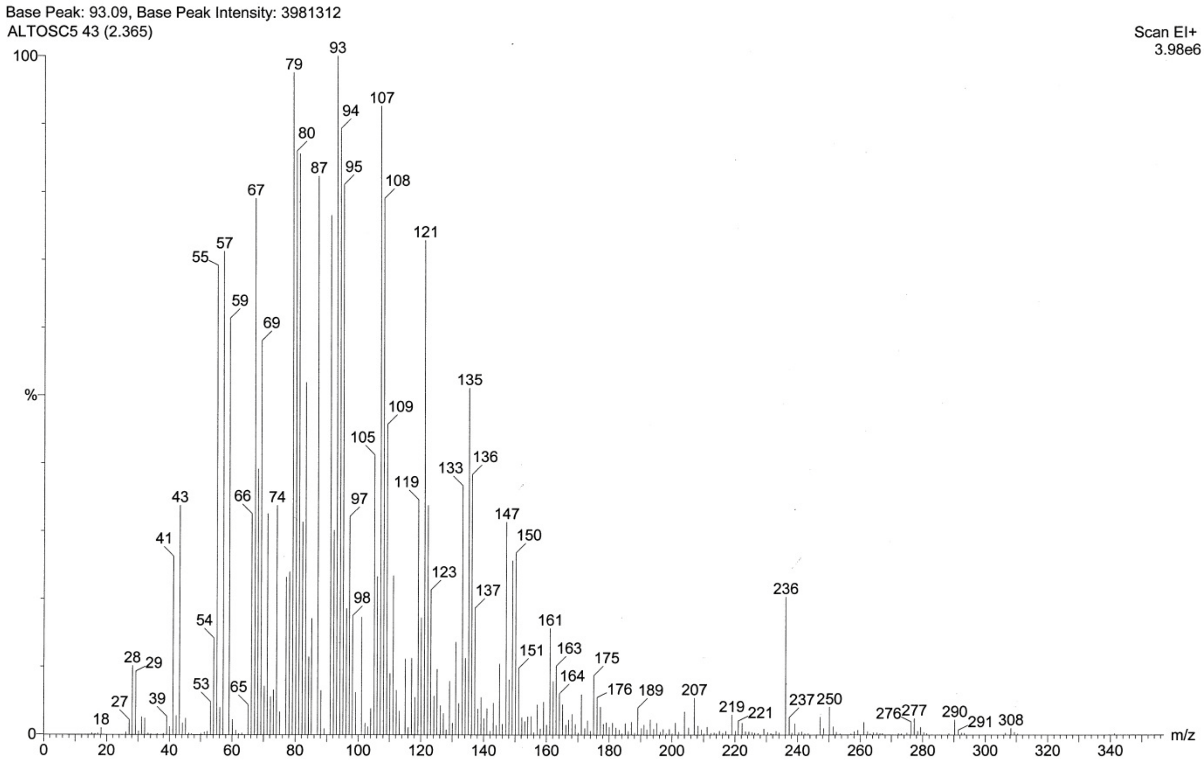

Figure S69. Mass spectrum of **22**.

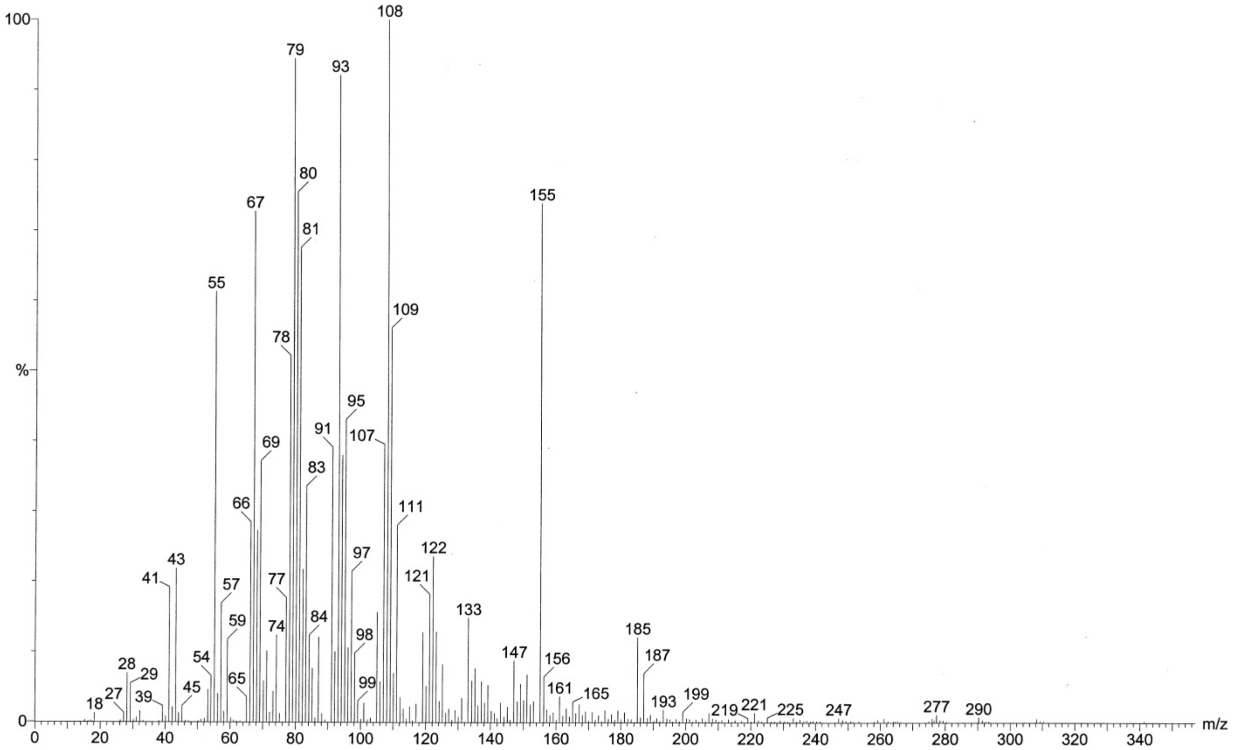

Supplement: Supplementary file 1 [file molecules-18-13754-s001.pdf]
